# Targeted inhibition of RBPJ transcription complex alleviates the exhaustion of CD8^+^ T cells in hepatocellular carcinoma

**DOI:** 10.1038/s42003-023-04521-x

**Published:** 2023-01-30

**Authors:** Banglun Pan, Zengbin Wang, Xiaoxia Zhang, Shuling Shen, Xiaoling Ke, Jiacheng Qiu, Yuxin Yao, Xiaoxuan Wu, Xiaoqian Wang, Nanhong Tang

**Affiliations:** 1 Department of Hepatobiliary Surgery and Fujian Institute of Hepatobiliary Surgery, Fujian Medical University Union Hospital, Fuzhou, China; 2 Cancer Center of Fujian Medical University, Fujian Medical University Union Hospital, Fuzhou, China; 3 Key Laboratory of Ministry of Education for Gastrointestinal Cancer, Fujian Medical University, Fuzhou, China

**Keywords:** Hepatocellular carcinoma, Immunosuppression, Cancer microenvironment

## Abstract

Impaired function of CD8^+^ T cells in hepatocellular carcinoma (HCC) is an important reason for acquired resistance. Compared with single-target inhibitors, small-molecule compounds that could both inhibit tumor cells and alleviate T cell exhaustion are more promising to reduce resistance. In this study, we screened immunosuppressive targets in HCC by combining cancer–immunity cycle score with weighted gene co-expression network and system analysis. Through in vitro and in vivo validation experiments, we found that one of the screened molecules, recombination signal binding protein for immunoglobulin kappa J region (RBPJ), was negatively correlated with CD8^+^ T cell mediated killing function. More importantly, its transcription complex inhibitor RIN1 not only inhibited the malignant biological behaviors of HCC cells by inhibiting mTOR pathway, but also reduced the expression of PD-L1 and L-kynurenine synthesis in HCC cells, thus alleviating T cell exhaustion. Meanwhile, the combination of RIN1 and anti-PD-1/PD-L1 antibodies could further activate CD8^+^ T cells. In short, RBPJ is an important factor regulating the function of T cells. Target inhibition of RBPJ transcription complex by small molecule compound may be a new strategy for immunotherapy of HCC.

## Abstract (web summary)

In vitro and in vivo validation of Cancer Genome Atlas screening results demonstrate that RBPJ is an important factor regulating the function of T cells in hepatocellular carcinoma.

## Introduction

Resistance to tumor immunotherapy is a complex and multi-mechanism interdependent dynamic process, including impaired immune cell infiltration, T cell exhaustion, recruitment of immunosuppressive cells, and epigenetic alternation, etc^1^. Among them, the exhaustion characteristics of T cell, especially CD8^+^ T cells, have received continuous attention and exploration. CD8^+^ T cells are the main tumor-infiltrating lymphocytes in hepatocellular carcinoma (HCC) and are prone to exhaustion upon stimulation by tumor-specific antigens. The anti-tumor immune response mediated by CD8^+^ T cells is limited by various mechanisms: high expression of immune regulatory molecules (such as PD-1, PD-L1, IDO1, etc.) in leukocytes or tumor cells, physicochemical imbalances of microenvironment (such as low pH, hypoxia, and amino acid metabolism disorder), metabolic competition with tumor cells, and lack of CD4^+^ T cell help^2^. Immunotherapies targeting PD-1/PD-L1 can reactivate CD8^+^ T cells at the tumor site. However, the objective response rate of HCC treated with Nivolumab (anti-PD-1 antibody) or Atezolizumab (anti-PD-L1 antibody) is not >20%^3^. Therefore, finding new and more effective targets to alleviate CD8^+^ T cell exhaustion is the focus of current immunotherapy research.

The clonal expansion disorder and functional impairment of tumor antigen-specific T cells are important reasons for the acquired resistance of patients to the inhibitors. Preventing or alleviating the exhaustion of CD8^+^ T cells could make the inhibitors exert a satisfactory effect^4^. Compared with single-target inhibitors, small-molecule compounds that both inhibit PD-L1 expression in tumor cells and disrupt PD-1 activity on CD8^+^ T cells are more promising to enhance the recognition and killing of tumor cells by CD8^+^ T cells. In this study, based on a cancer-immune cycle weighted gene co-expression network analysis and in vivo/in vitro experiments, we found that recombination signal binding protein for immunoglobulin kappa J region (RBPJ, an inhibitory transcription factor of Notch pathway^5^) could be used as a biomarker to evaluate “the killing of CD8^+^ T cells targeting tumor cells” and “the biological function of HCC cells”. Its transcription complex inhibitor RIN1 inhibited the occurrence and development of HCC by enhancing the activity of infiltrating leukocytes (especially CD8^+^ T cells) and inhibiting tumor cell proliferation and epithelial-mesenchymal transition. This study shed light on a better understand the relationship between HCC cells and microenvironment, and identified RIN1 as a potential immunotherapy compound.

## Results

### RBPJ was an immunosuppressive target in HCC

**Fig. 1:**
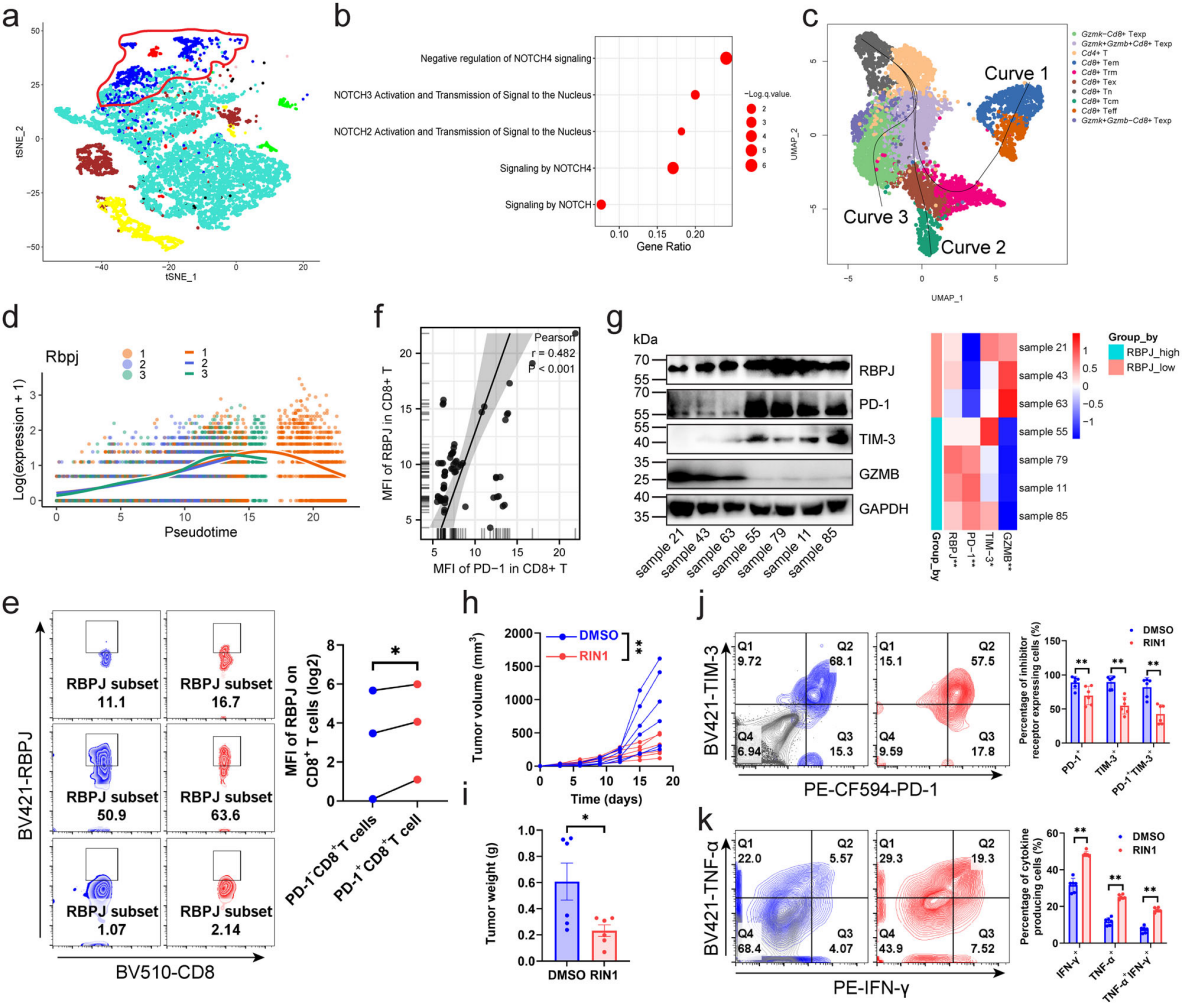
RBPJ was an immunosuppressive target in HCC. **a** TCGA database was used to construct a WGCNA network, with each color representing a cluster and each node representing a gene (*n* = 371). **b** KEGG enrichment analysis was performed to analyze the enriched pathways of genes in the blue cluster. The size of the dots indicated the significance of the enrichment. **c** UMAP plot of T cells from five mouse HCC tissues. The pseudo-time trajectories of T cells analyzed by Slingshot algorithm (*n* = 5). **d** Expression score of *Rbpj* on three trajectories of mouse infiltrating CD8^+^ T cells (*n* = 5). **e** Expression of RBPJ in PD-1^-^ CD8^+^ T cells and PD-1^+^ CD8^+^ T cells from mouse primary HCC (*n* = 3). Blue indicated PD-1^-^ CD8^+^ T cells, and red indicated PD-1^+^ CD8^+^ T cells. **f** Flow cytometry detecting the correlation between RBPJ and PD-1 in infiltrating CD8^+^ T cells from patients with HCC (*n* = 63). **g** Tumor-infiltrating CD8^+^ T cells from patients with HCC were sorted with magnetic beads and the expression of RBPJ, PD-1, TIM-3, GZMB was detected using western blot (*n* = 7). **h**, **i** Growth curves (**h**) and weight plot (**i**) showing the effects of RIN1 (50 mg/kg, *i.v*.) on the growth of subcutaneous tumors (*n* = 6). **j**, **k** Expression of inhibitory receptors (**j**, *n* = 5) and cytokines (**k**, *n* = 6) on mouse HCC infiltrating CD8^+^ T cells under RIN1 (50 mg/kg, *i.v*.). Gray indicated isotype control. Mean ± SD. Statistical significance determined by Pearson’s rank correlation coefficient (**f**) and paired two-tailed *t*-test (**e**, **g**–**k**). *P < 0.05; **P < 0.01.

Infiltration of leukocytes in tumors and their recognition of tumor-specific antigens are important indicators to measure the effect of immune checkpoint inhibitors. We analyzed the relationship between gene expression and seven steps (including step1: release of cancer cell antigens; step2: cancer antigens presentation; step3: priming and activation; step4: trafficking of leukocytes to tumors; step5: infiltration of leukocytes into tumors; step6: recognition of cancer cells by T cells; step7: killing of cancer cells) of the cancer-immune cycle in HCC by weighted correlation network analysis (WGCNA) based on the Cancer Genome Atlas (TCGA) and Tracking Tumor Immunophenotype (TIP) databases. Cluster analysis divided all genes into nine modules (Fig. 1a). The blue module exhibited negative correlation with steps 5 and 7 (“infiltration of leukocytes into tumors” and “killing of cancer cells”) (Supplementary Fig. 1). To identify the immunosuppressive targets in HCC, we focused on the blue module. KEGG pathway enrichment analysis was used to find out which signaling pathway the blue module was regulated by. The Notch signaling pathway was noted to be significantly enriched on this module (Fig. 1b). RBPJ is a key transcriptional regulator of Notch pathway, acting as a transcriptional activator when it binds to NOTCH1 and a transcriptional repressor when NOTCH1 is absent^5^. To investigate whether the genes in the blue module were dependent on RBPJ for preventing “infiltration of leukocytes into tumors” and “killing of cancer cells”, TCGA database and patient HCC samples were used to analyze the relationship between RBPJ and inhibitory receptors. RBPJ expression was significantly positively correlated with various inhibitory receptors, including PDCD1, HAVCR2, CTLA4, LAG3, and TIGIT (Supplementary Fig. 2a-c), but not with immune cell infiltration (Supplementary Fig. 2d-e). To further determine how RBPJ regulated HCC infiltrating CD8^+^ T cells, single-cell RNA-seq (scRNA-seq) was performed to analyze T cells in mouse HCC. We employed Seurat algorithm for T cell classification and marker gene identification. Ten T cell subpopulations were identified and visualized using Uniform Manifold Approximation and Projection (UMAP). Cluster-specific genes were used to annotate cell types: *Gzmk*^−^*Cd8*^+^ T_exp_ (*Gzmk*^−^*Pdcd*-1^+^*Havcr2*^+^*Ctla4*^+^*Cd8*^+^), *Gzmk*^+^*Gzmb*^+^*Cd8*^+^ T_exp_ (*Gzmk*^+^*Gzmb*^+^*Pdcd1*^+^*Havcr2*^+^*Ctla4*^+^*Cd8*^+^), *Cd4*^*+*^ T (*Cd4*^+^), *Cd8*^+^ T_em_ (*Cxcr3*^+^*Cxcr4*^+^*Cd44*^+^*Ifng*^+^*Cd8*^+^), *Cd8*^+^ T_rm_ (*Cd6*^+^*Xcl1*^+^*Capg*^+^*Cd69*^+^*Nr4a1*^+^*Nr4a2*^+^*Cd8*^+^), *Cd8*^+^ T_ex_ (*Mir155hg*^+^*Tnfrsf9*^+^*Ctla4*^+^*Pdcd1*^+^*Lag3*^+^*Tox*^+^*Gzmk*^+^*Gzmb*^+^I*fng*^+^*Cd8*^+^), *Cd8*^+^ T_n_ (*Lef1*^+^*Tcf7*^+^*Ccr7*^+^*Sell*^+^*S1pr1*^+^), *Cd8*^+^ T_cm_ (*Cd28*^+^*Cd27*^+^*Prf1*^+^), *Cd8*^+^ T_eff_ (*Ifng* ^+^*Cxcr3*^+^*Tbx21*^+^*Eomes*^+^*Gzmk*^+^*Gzmb*^+^*Cd8*^+^), and Gzmk^+^Gzmb^−^Cd8^+^ T_exp_ (Gzmk^+^Gzmb^−^*Pdcd1*^+^*Havcr2*^+^*Ctla4*^+^*Ifng*^+^*Cd8*^+^) (Fig. 1c, Supplementary Fig. 3). Pseudotime analysis inferred the existence of three trajectories of T cells. The first trajectory (Curve 1) developed from *Cd8*^+^ T_n_, *Gzmk*^+^*Gzmb*^+^*Cd8*^+^ T_exp_, *Cd8*^+^ T_rm_, *Cd8*^+^ T_eff_ to *Cd8*^+^ T_em_; the second trajectory (Curve 2) developed from *Cd8*^+^ T_n_, *Gzmk*^+^*Gzmb*^+^*Cd8*^+^ T_exp_ to *Gzmk*^−^*Cd8*^+^ T_exp_; the third trajectory (Curve 3) developed from *Cd8*^+^ T_n_, *Gzmk*^+^*Gzmb*^+^*Cd8*^+^ T_exp_, *Cd8*^+^ T_exp_ to *Cd8*^+^ T_em_ (Fig. 1c). With the extension of the second and third trajectories, the expression of *Rbpj* gradually increased. While the expression of *Rbpj* on the first trajectory had an inflection point, peaking when T cells developed to *Cd8*^+^ T_rm_, but inhibited when cells converted to *Cd8*^+^ T_eff_ (Fig. 1d). This implied that as T cells developed from naïve to precursor exhaustion or exhaustion, the expression of *Rbpj* was gradually induced, but its expression was inhibited if the cell reactivated into effector or central memory. The simultaneous change of RBPJ with T cell exhaustion mean that it was an important regulator of tumor infiltrating CD8^+^ T cells. To verify this hypothesis, we conducted the next study.

The relationship between RBPJ expression and exhausted CD8^+^ T cells was then verified using mouse and patient HCC specimens. Supplementary Table 1 presented clinical information about the patients enrolled in the study. Mouse HCC infiltrating CD8^+^ T cells were classified as PD-1-negative and PD-1-positive based on PD-1 expression. The expression of RBPJ in PD-1^+^ CD8^+^ T cells was higher than that in PD-1^−^ CD8^+^ T cells (Fig. 1e). In line with this, PD-1 in patient HCC infiltrating CD8^+^ T cells showed a strong positive correlation with RBPJ (Fig. 1f). In addition to PD-1, CD8^+^ T cells with high expression of RBPJ were also induced to express higher TIM-3, while GZMB expression was significantly inhibited in patients with HCC (Fig. 1g).

Then, we further employed RIN1, a RBPJ transcription complex inhibitor (which disrupts the interaction between RBPJ and SHARP and inhibits Notch pathway^6^), to reversely verify the role of RBPJ, and found that RIN1 inhibited PD-1 expression on CD8^+^ T cells from subcutaneous tumors of mouse with HCC (Supplementary Fig. 2f). However, the infiltration of CD4^+^/CD8^+^ T cells, NK cells and macrophages was not affected (Supplementary Fig. 2f). Meanwhile, we also found that RIN1 inhibited tumor growth (Fig. 1h-i) and improved the activity of CD8^+^ T cells (Fig. 1j-k). Compared with nude mice, the inhibitory effect of RIN1 on HCC was more pronounced in C57BL/6 J, suggesting that RIN1 has a non-negligible immunoregulatory role (Supplementary Fig. 2g).

### RBPJ-*Rps16* axis induced HCC infiltrating CD8^+^ T cell exhaustion

**Fig. 2:**
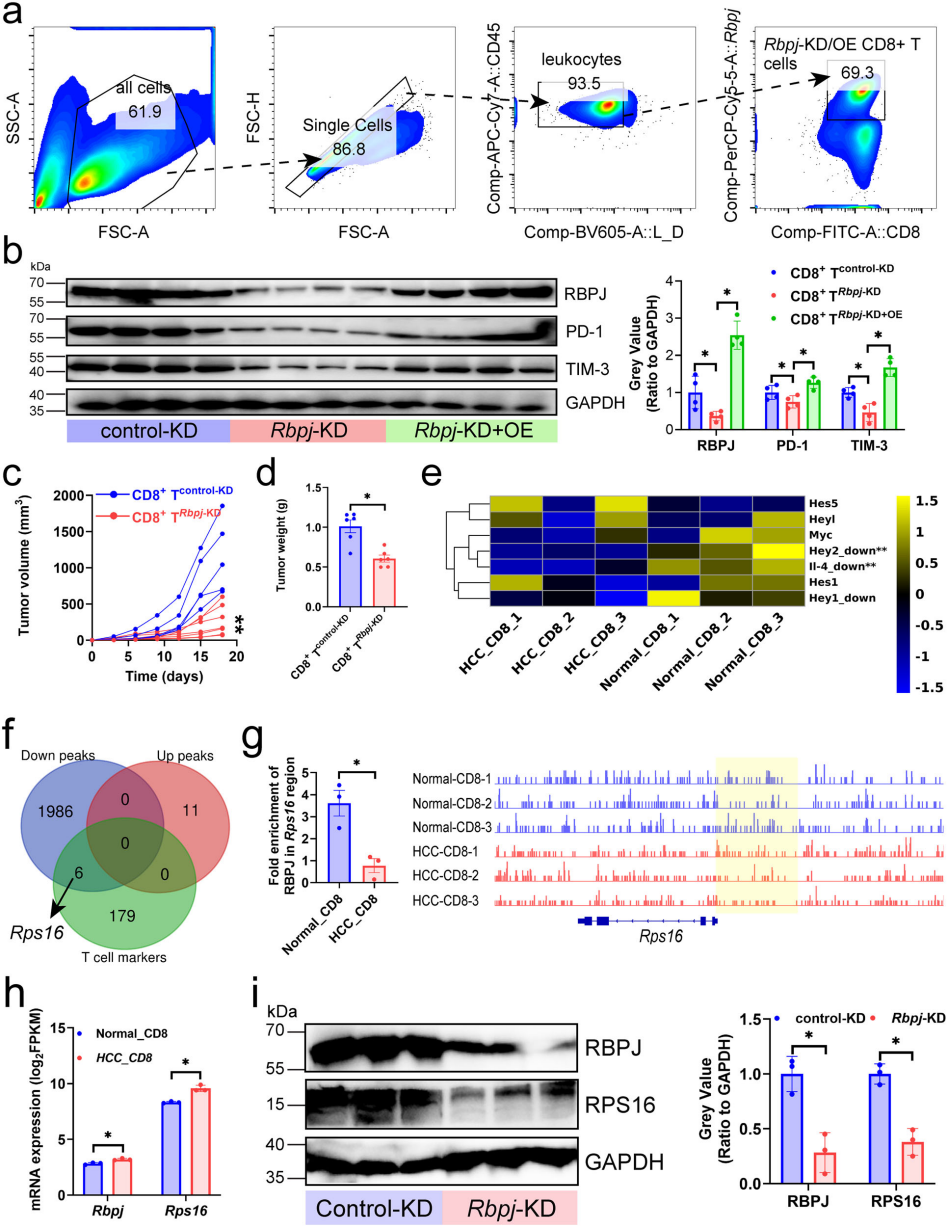
Inhibition of *Rbpj* in CD8^+^ T cells alleviated CD8^+^ T cell exhaustion. **a** Gating strategy. Mouse HCC infiltrating CD8^+^ T cells were purified by magnetic beads, and CD8^+^ T cells with overexpression or knockdown of *Rbpj* were obtained by flow cytometry. **b** Effect of knockdown and overexpression of *Rbpj* in CD8^+^ T cells (CD8^+^ T^*Rbpj*-KD^/CD8^+^ T^*Rbpj*-KD+OE^) on inhibitory receptor expression in mouse HCC infiltrating CD8^+^ T cells (*n* = 4). **c**, **d** Growth curves (**c**) and weight plot (**d**) showing the effects of CD8^+^ T^*Rbpj*-KD^/CD8^+^ T^*Rbpj*-KD+OE^ on the growth of the subcutaneous tumors (*n* = 6). **e** mRNA expression of the target genes of the Notch signaling pathway in mouse normal liver and HCC-infiltrating CD8^+^ T cells (*n* = 3). **f** Venn diagram showing the intersection of T cell-related genes, derived from the CellMarker database (http://xteam.xbio.top/CellMarker/), and the genes to which RBPJ enriched peaks belong (*n* = 3). Enrichment peaks with *P-*value < 0.05 and |log_2_(fold change)| > 1. **g** *Rps16* locus with normalized CUT&Tag signals for mouse normal liver and HCC-infiltrating CD8^+^ T cells (*n* = 3). Yellow highlighting calls attention to peaks that differ most strikingly between two groups. **h** mRNA expression of *Rbpj* and *Rps16* in mouse normal liver and HCC-infiltrating CD8^+^ T cells (*n* = 3). **i** CD8^+^ T^*Rbpj*-KD^ inhib**i**ted the protein expression of RPS16 in mouse HCC infiltrating CD8^+^ T cells (*n* = 3). Mean ± SD. Statistical significance determined by paired two-tailed *t*-test (**b**–**e**, **g**–**i**). *P < 0.05; **P < 0.01.

It seemed that RBPJ expressed in CD8^+^ T cells resulted in or have positive relationship with CD8^+^ T cell exhaustion, we further investigated the direct effect of RBPJ on CD8^+^ T cells. We knocked down and overexpressed *Rbpj* in mouse CD8^+^ T cells (CD8^+^ T^*Rbpj*-KD^/CD8^+^ T^*Rbpj*-KD+OE^) and adopted them into primary HCC mice (*i.v*.) to study whether knockdown of *Rbpj* alleviated T cell exhaustion (Fig. 2a). We found that CD8^+^ T^*Rbpj*-KD^ expressed lower PD-1 and TIM-3, and CD8^+^ T^*Rbpj*-KD+OE^ was able to rescue this negative change (Fig. 2b). In addition, CD8^+^ T^*Rbpj*-KD^ showed stronger inhibition of tumor growth (Fig. 2c-d).

We next investigated the molecular mechanism of how knockdown of *Rbpj* alleviated exhaustion of HCC-infiltrating CD8^+^ T cells in vivo. Mouse normal liver and HCC infiltrating CD8^+^ T cells were sorted out and subjected to transcriptome and Cleavage Under Targets and Tagmentation (CUT&Tag) sequencing to identify which gene promoter RBPJ acted on to drive T cell exhaustion. In the absence of Notch activation, RBPJ acts as a transcriptional repressor in complex with a growing list of co-repressors, linker proteins, and enzymes such as histone deacetylases. In the nucleus the Notch intracellular domain (NICD) displaces transcriptional repressors and forms a complex with RBPJ and Mastermind-like (MAML). MAML recruits transcriptional co-activators, such as the histone acetyltransferase p300, forming a Notch activator complex that culminates in the transcription of Notch target genes^7^. The expression of Hey2 and IL-4 (Notch target genes) in HCC infiltrating CD8^+^ T cells was significantly lower than that in normal liver, indicating that Notch signaling was inhibited in former (Fig. 2e). Given the dependence of RBPJ transcriptional activity on NICD signaling, we hypothesized that the increased expression of target genes positively regulated by RBPJ in HCC-infiltrating CD8^+^ T cells were accompanied by the absence of RBPJ enrichment peaks in their promoters. RBPJ was significantly absent from the promoter of six T cell-associated genes (*Id2*^8^, *Rel*^9^, *Rps16*^10^, *Tpt1*^11^, *Rapgef6*^12^, and *Stat3*^13^, collected from CellMarker database) (Fig. 2f), among which *Rps16* has been reported to regulate CD8^+^ T cell infiltration and function^10^. The RBPJ enrichment peaks at the *Rps16* promoter in HCC were found to be decreased (Fig. 2g) and accompanied by higher expression (Fig. 2h). Similarly, RPS16 expression on mouse HCC infiltrating CD8^+^ T cells was inhibited by *Rbpj*-KD (Fig. 2i). As a result, in HCC infiltrating CD8^+^ T cells with Notch signal-deficient, the reduction of RBPJ in the promoter region of the exhausted regulator *Rps16* enhanced the expression of latter, which drove T cell exhaustion.

### RIN1 regulated T cell components in HCC

**Fig. 3:**
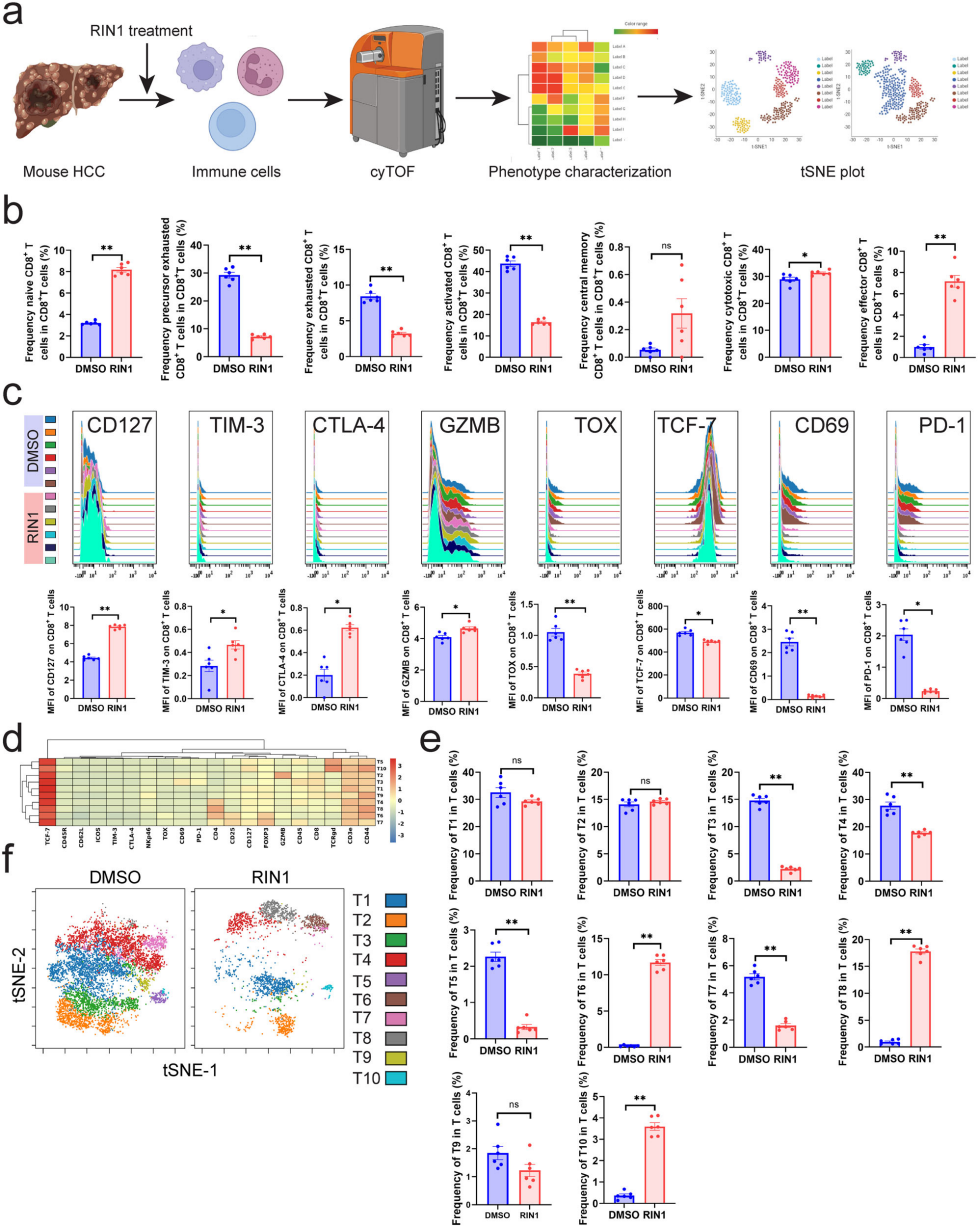
CyTOF revealed the regulatory effect of RIN1 on infiltrating T cells in mouse primary HCC. **a** Experimental design and analysis workflow of cyTOF (all the components were from BioRender (https://biorender.com/)). **b** Frequency of mouse HCC infiltrating CD8^+^ T cell subpopulations (*n* = 6). **c** Expression of selected functional markers on mouse HCC infiltrating CD8^+^ T cells (*n* = 6). **d** Heatmap of corrected median expression of ten T cell subpopulations (*n* = 6). **e** t-SNE plot of mouse HCC infiltrating T cells (*n* = 6). **f** Comparison of the proportions of ten T cell subpopulations (*n* = 6). Mean ± SD. Statistical significance determined by paired two-tailed *t*-test (**b**, **c**, **e**). *P < 0.05; **P < 0.01.

Since RIN1 (Fig. 1J-K) and inhibition of *Rbpj* (Fig. 2) were able to improve the function of HCC infiltrating CD8^+^ T cells, we used mass cytometry by the time-of-flight (cyTOF) to comprehensively analyze the remodeling effect of RIN1 on T cells in vivo. (Fig. 3a). Predefined marker modules were specifically designed to describe the frequency and function of leukocytes. To accurately characterize the leukocytes, we collected a total of 5 million mouse leukocytes. The proportion of cytotoxic (GZMB^+^)/effector (CD44^+^ CD62L^−^)/naïve (CD44^−^ CD62L^+^) CD8^+^ T cells was increased, accompanied by a decrease in precursor exhausted (PD-1^+^ TIM-3^−^)/exhausted (PD-1^+^ TIM-3^+^) CD8^+^ T cells (Fig. 3b). Consistently, RIN1 induced an increase in CD127 (used to describe activity), GZMB (marker of cytotoxicity) and a decrease in TOX, PD-1 (indicators of exhaustion) in CD8^+^ T cells (Fig. 3c). However, TIM-3, CTLA-4 (indicators of exhaustion) increased after treatment, while TCF-7 (used to mark stem cell-like phenotypes), CD69 (used to describe activity) decreased, which might be due to RIN1 destroying the activity of CD8^+^ T cells (Fig. 3c). The previous results (Figs. 1–2) reminded us that RIN1 exhibited a superior role in alleviating CD8^+^ T cell exhaustion, so we further explored its regulatory effect on various T cell subpopulations. We found that T3 (CD3e^+^ CD8a^+^ PD-1^+^, exhausted CD8^+^ T cells), T4 (CD3e^+^ CD4^+^ TCF-7^+^, stem cell-like CD4^+^ T cells), T5 (CD3e^+^ TCRgd^+^ TCF-7^+^, TCF-7^+^ γδ T cells), T7 (CD3e^+^ CD4^+^ CD25^+^ FOXP3^+^ TOX^-^, TOX^-^ Treg), T9 (CD3e^+^ CD8a^+^ CD44^+^ CD62L^-^, effector CD8^+^ T cells) decreased in RIN1-treated group with T6 (CD3e^+^ CD4^+^ CD25^+^ FOXP3^+^ TOX^+^, TOX^+^ Treg), T8 (CD3e^+^ CD4^+^CD44^+^CD62L^-^, effector CD4^+^ T cells), T10 (CD3e^+^ TCRgd^+^ TCF-7^-^, TCF-7^−^ γδ T cells) elevated (Fig. 3d-f). In short, RIN1 had an important regulatory role in preventing CD8^+^ T cell exhaustion.

### Regulatory effect of *HCC*^*RIN1-sup*^ on the transcriptional profile of CD8^+^ T cells

Subsequently, we compared the immunopotentiation of RIN1, *Rbpj* knockdown in Hepa1-6 cells (Hepa1-6^*Rbpj*-KD^) and CD8^+^ T^*Rbpj*-KD^. RIN1 showed a stronger inhibitory effect on tumor growth than Hepa1-6^*Rbpj*-KD^ (Supplementary Fig. 4a–c), which suggested that RIN1 inhibition of tumor growth did not rely solely on blocking the assembly of RBPJ transcription complex in tumor cells. We then attempted to analyze whether RIN1 could improve the anti-tumor immune response of CD8^+^ T cells in vitro. We adopted RIN1-stimulated CD8^+^ T cells in vitro (CD8^+^ T^RIN1^) into mice with HCC to evaluate their role in regulating tumor growth. In contrast to CD8^+^ T^*Rbpj*-KD^ (Fig. 2c-d), CD8^+^ T^RIN1^ promoted tumor growth, and this positive effect was inhibited by anti-PD-1 antibody, indicating that RIN1 in vitro did not simulate the protective effect of CD8^+^ T^*Rbpj*-KD^ (Supplementary Fig. 4d-e). Consistent with this, CD8^+^ T^RIN1^ was induced to express higher PD-1/TIM-3, and IFN-γ/TNF-α were inhibited (Supplementary Fig. 4f-g). The above results indicated that although *Rbpj* induced the expression of inhibitory receptors and RIN1 was able to well simulate the role of CD8^+^ T^*Rbpj*-KD^ in vivo, RIN1 enhancement of T cells does not act directly on T cells, but probably indirectly through HCC cells.

**Fig. 4:**
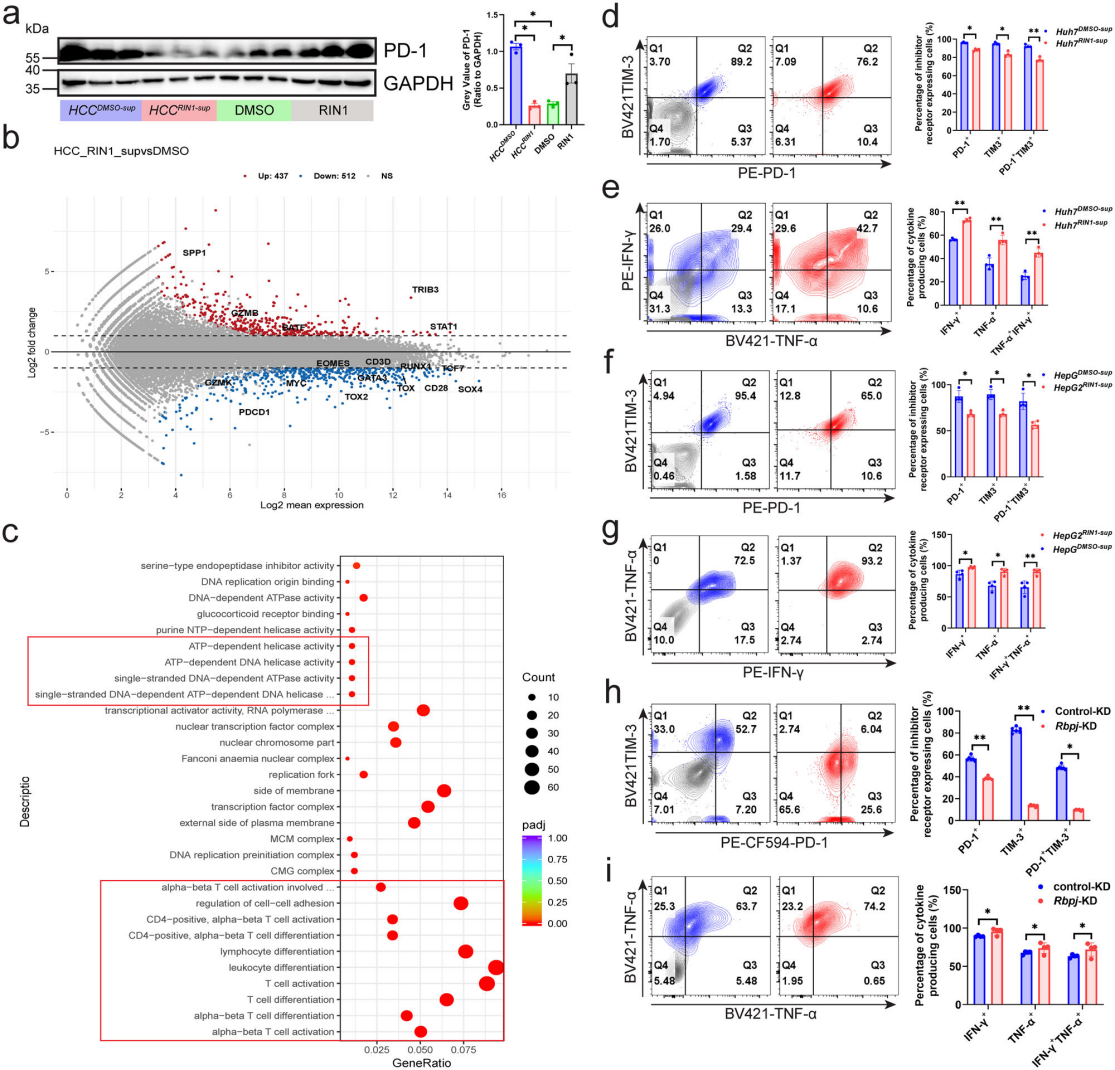
Regulatory effect of *HCC*^*RIN1-sup*^ on the transcriptional profile of CD8^+^ T cells. **a** Western blot showing PD-1 expression in mouse HCC infiltrating CD8^+^ T cells (*n* = 3). **b** MA plot of RNA-seq data from *Huh7*^*DMSO-sup*^ versus *Huh7*^*RIN1-sup*^-treated patient HCC infiltrating CD8^+^ T cells (*n* = 3). Genes with elevated expression in the *Huh7*^*RIN1-sup*^-treated CD8^+^ T cells (*P*_adj_ < 0.05 and log2(fold change) > 1) were marked in red, genes with reduced expression (*P*_adj_ < 0.05 and log2(fold change) < −1) were highlighted in blue. Selected genes were labeled. **c** GO enrichment analysis of RNA-seq data from *Huh7*^*DMSO-sup*^ versus *Huh7*^*RIN1-sup*^-treated CD8^+^ T cells (*n* = 3). **d**–**g** Expression of inhibitory receptors (**d**, **f**) and cytokines (**e**, **g**) on CD8^+^ T cells from *Huh7*^*RIN1-sup*^ (**d**, **e**) and *HepG2*^*RIN1-sup*^ (**f**, **g**) (*n* = 4). Gray indicated isotype control. **h**, **i** Expression of inhibitory receptors (**h**) and cytokines (**i**) on CD8^+^ T cells from the tumors of control-KD and *Rbpj*-KD (*n* = 7). Gray indicated isotype control. Mea*n* ± SD. Statistical significance determined by paired two-tailed *t*-test (**a**, **b**, **d**–**i**). *P < 0.05; **P < 0.01.

Thus, we used the supernatant of RIN1-treated HCC cells (*HCC*^*RIN1-sup*^) to treat T cells to observe how RIN1 indirectly acted on T cells by affecting HCC cells. Given RIN1 could still be present in the supernatant and directly affect CD8^+^ T cells one way or another and could promote T cell activation by itself, we compared the effects of DMSO, RIN1, *HCC*^*DMSO-sup*^ and *HCC*^*RIN1-sup*^ on patient HCC infiltrating CD8^+^ T cells. Consistent with Supplementary Fig. 4d-g, RIN1 induced PD-1 expression, whereas *HCC*^*RIN1-sup*^ suppressed PD-1 expression, implying that RIN1 indirectly alleviated T cell exhaustion by affecting HCC cells (Fig. 4a). In addition, PD-1 expression was induced by *HCC*^*DMSO-sup*^ compared with DMSO (Fig. 4a), which might be because certain inhibitory cytokines or metabolites secreted by HCC impair T cells^14–16^. And then we used *HCC*^*RIN1-sup*^ to stimulate CD8^+^ T cells (*HCC*^*RIN1-sup*^-*CD8*) for 48 h, and detected the alternations of their transcriptional profile. The results showed that (1) PDCD1 and transcription factors (EOMES, MYC, GATA3, TOX, TOX2, RUNX1, TCF7, SOX4) were downregulated in *HCC*^*RIN1-sup*^-CD8, which suggested that *HCC*^*RIN1-sup*^ has a positive effect on the key transcription factors of CD8^+^ T cells (Fig. 4b); (2) genes encoding downstream signaling (BATF, STAT1, and TRIB3) of IFNAR1 were upregulated, resulting in a stronger response of *HCC*^*RIN1-sup*^-CD8 to IFNα/β (Fig. 4b); (3) terms related to ATP-dependent energy metabolism and T cell activation were significantly enriched in *HCC*^*RIN1-sup*^-CD8 (Fig. 4c). These results pointed to the fact that *HCC*^*RIN1-sup*^ enhanced the function of CD8^+^ T cells. This inference was subsequently confirmed by in vitro co-culture experiments, which showed that *HCC*^*RIN1-sup*^*-CD8* expressed lower levels of PD-1/TIM3 (Fig. 4d, f) and higher levels of IFN-γ/TNF-α/Ki -67 (Fig. 4e, g, Supplementary Fig. 5a), and co-cultured HCC cells tended to be apoptotic (Supplementary Fig. 5b). In addition to inhibitors, inoculation of Hepa1-6 cells with knockdown of *Rbpj* also yielded consistent results. Infiltrating CD8^+^ T in subcutaneous tumors of *Rbpj*-KD exhibited stronger antitumor immune activity (Fig. 4h-i).

### RIN1 improved CD8^+^ T cell function by inhibiting L-kynurenine synthesis and PD-L1 expression

**Fig. 5:**
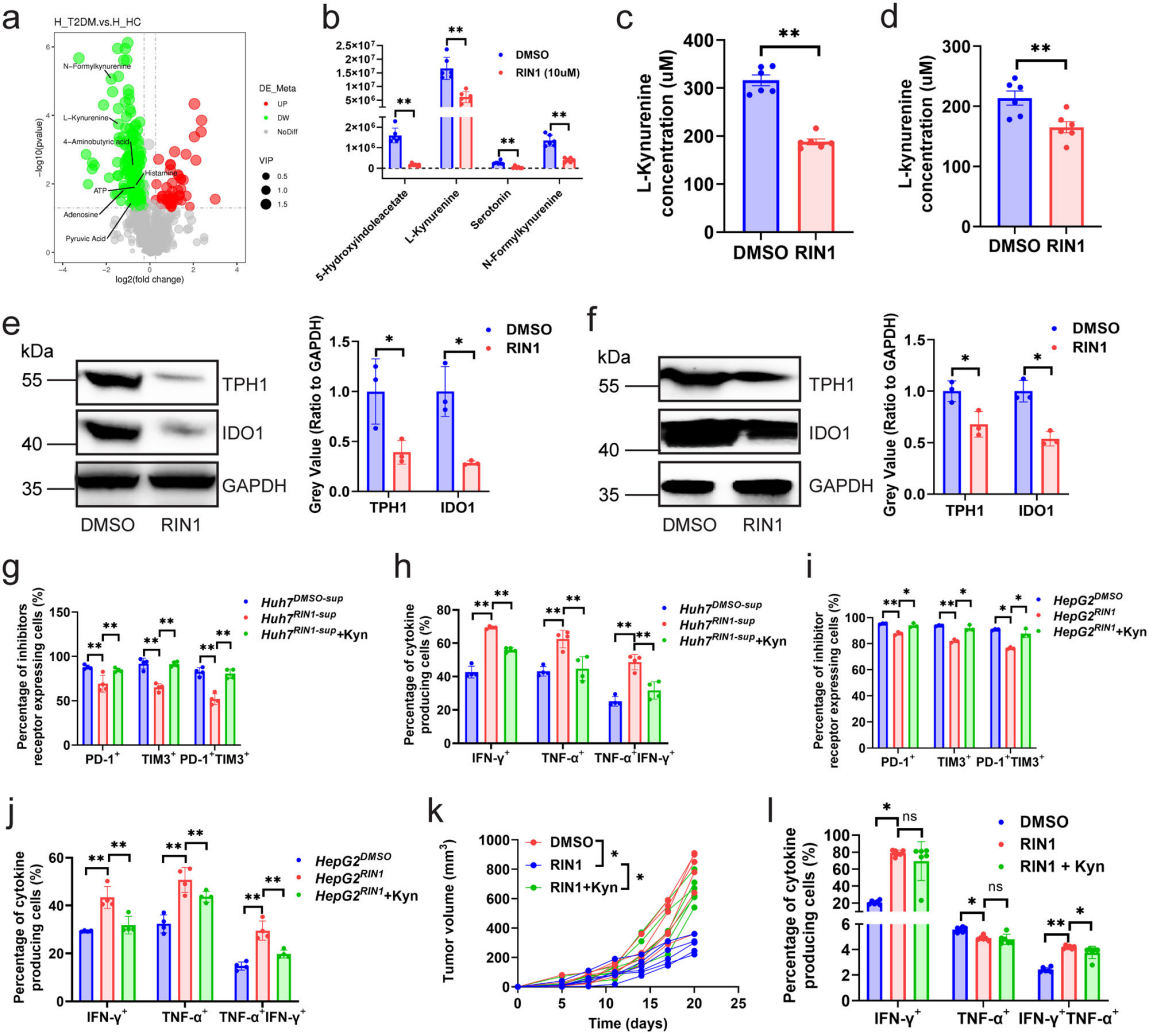
RIN1 improved CD8^+^ T cell function by inhibiting tryptophan metabolism. **a** Volcano plot of metabolome from Huh7 cells treated with DMSO and RIN1 (10 μM) (*n* = 3). Metabolites that were elevated in RIN1-treated cells (*P*_adj_ < 0.05 and log2(fold change) > 1) were marked in red, while decreased (*P*_adj_ <  0.05 and log2(fold change) < −1) were marked in blue. Selected metabolites were labeled. **b** Boxplots demonstrating the abundance of L-kynurenine, N-formylkynurenine, 4-aminobutyric acid, and serotonin in Huh7 cells (*n* = 6). **c**, **d** ELISA analysis of L-kynurenine secretion by Huh7 (**c**) and HepG2 (**d**) cells (*n* = 6). **e**, **f** Immunoblot showing the protein expression of TPH-1 and IDO1 in Huh7 (**e**) and HepG2 (**f**) cells (*n* = 3). **g**-**j** Inhibitory receptor (**g**, **i**) and cytokine (**h**, **j**) expression on patient HCC infiltrating CD8^+^ T cells from control, *HCC*^*RIN1-sup*^, and *HCC*^*RIN1-sup*^ with L-kynurenine (200 nM) groups, *HCC*^*RIN1-sup*^ included *Huh7*^*RIN1-sup*^ (**g**, **h**) and *HepG2*^*RIN1-sup*^ (**i**, **j**) (*n* = 4). **k**, **l** Tumor growth curves (**k**) and cytokine expression on CD8^+^ T cells in tumors (**l**) from control, intraperitoneal injection of RIN1 (50 mg/kg), and intraperitoneal injection of RIN1 with L-kynurenine (30 mg/kg) groups (*n* = 6). Mean ± SD. Statistical significance determined by paired two-tailed *t*-test (**a**–**l**). *P < 0.05; **P < 0.01.

Notch pathway has metabolic functions, as a downstream effector of the Notch pathway, inhibition of *Rbpj* dampens hepatic glucose production and increases hepatic lipid content^17^. To investigate how *HCC*^*RIN1-sup*^ regulated CD8^+^ T cells, we employed untargeted metabolomics to analyze metabolomic alternations in Huh7 cells treated by RIN1. Findings: (1) RIN1 inhibited tryptophan metabolism (decreased secretion of 5-hydroxyindoleacetate, serotonin, N-formylkynurenine, and L-kynurenine) (Fig. 5a, b). Cytological experiments confirmed that RIN1 did decrease L-kynurenine secreted by Huh7 and HepG2 cells (Fig. 5c, d), which was caused by the reduction of TPH-1 and IDO1 expression from the translational level (Fig. 5e, f) rather than the transcriptional level (Supplementary Fig. 6a, b). IDO1-IN-18 (IDO1 inhibitor) and Rodatristat (TPH1 inhibitor) did reduce the promotion of L-kynurenine by RBPJ (Supplementary Fig. 6c). The protein expression of IDO1 and TPH1 (Supplementary Fig. 6d) was significantly decreased in HCC with low expression of RBPJ compared to which with high expression of RBPJ. (2) L-kynurenine attenuated the activation of *HCC*^*RIN1-sup*^ on CD8^+^ T cells (Fig. 5g–j, Supplementary Fig. 7a–d), indicating that the enhanced function of *HCC*^*RIN1-sup*^-*CD8* was attributed to the decreased secretion of L-kynurenine from HCC cells. In vivo experiments also confirmed that L-kynurenine reduced the antitumor effect of RIN1, including increased tumor growth (Fig. 5k) and downregulated cytokine expression on mouse HCC infiltrating CD8^+^ T cells (Fig. 5l, Supplementary Fig. 7e). We then analyzed HCC infiltrating regulatory T cells (Tregs) under RIN1 and L-kynurenine, because it has been known that L-kynurenine also induces Tregs^18^. It could be seen that RIN1 did not influence the infiltration of Treg (Supplementary Fig. 8a, b), which was consistent with the results of Fig. 3d–f. This indicated that the regulatory effect of RIN1 on cytotoxic CD8^+^ T cells was independent of Tregs.

**Fig. 6:**
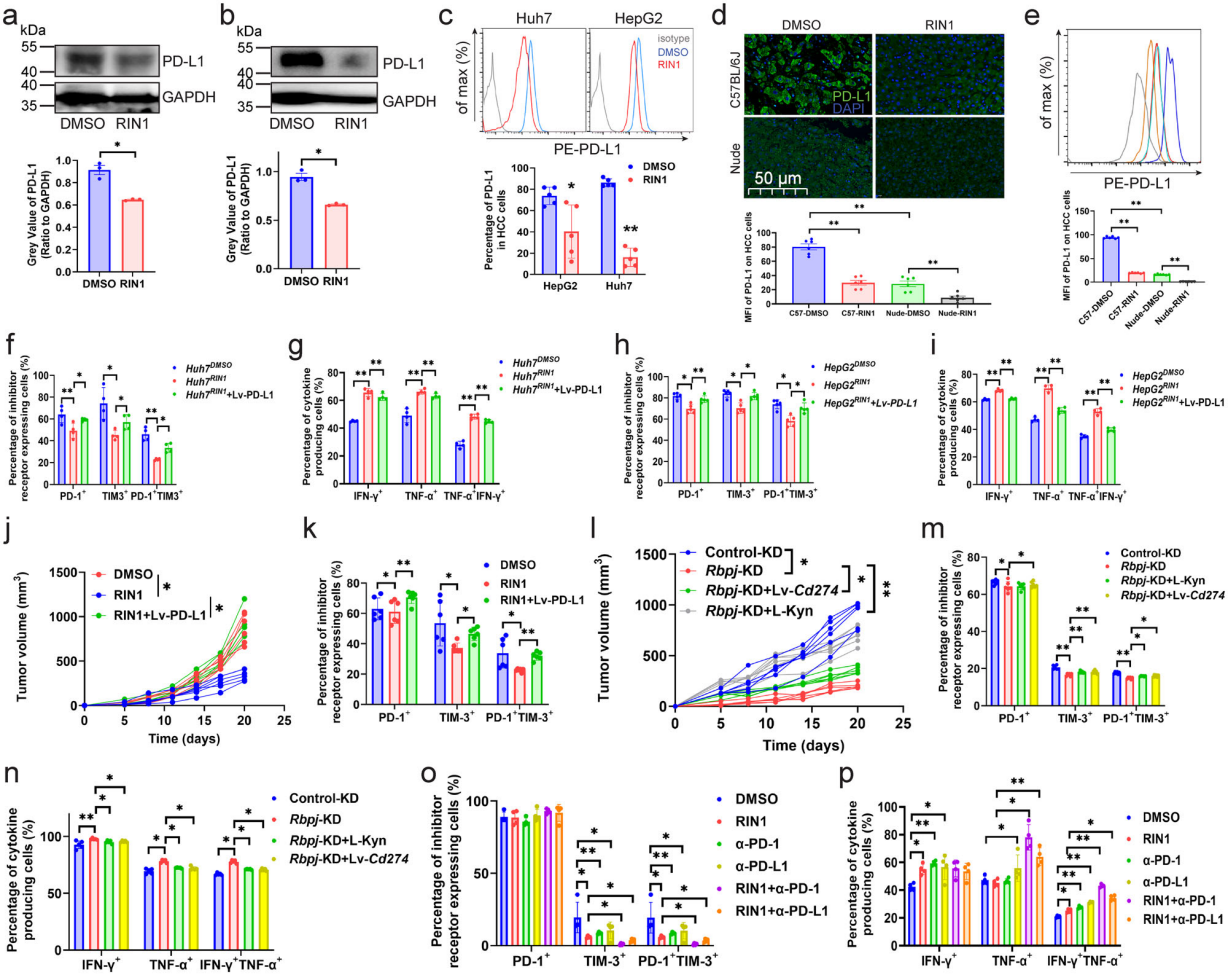
RIN1 improved CD8^+^ T cell function through the PD-1/PD-L1 axis. **a**, **b** Immunoblot showing the effect of RIN1 (10 μM) on PD-L1 protein expression in Huh7 (**a**) and HepG2 (**b**) cells (*n* = 3). **c** Flow cytometry analysis of PD-L1 protein expression on the surface of Huh7 and HepG2 cells from control and RIN1 (10 μM)-treated groups (*n* = 5). **d**, **e** Immunohistochemistry and flow cytometry showing PD-L1 protein expression in HCC cells from subcutaneous tumor models (*n* = 6). **f**–**i** Inhibitory receptor (**f**, **h**) and cytokine (**g**, **i**) expression on CD8^+^ T cells from control, *HCC*^*RIN1*^, and *HCC*^*RIN1*^ + Lv-PD-L1 groups, *HCC*^*RIN1*^ included *Huh7*^*RIN1*^ (**f**, **g**) and *HepG2*^*RIN1*^ (h-i) (*n* = 4). **j**, **k** Tumor growth curves (**j**) and inhibitory receptor expression on CD8^+^ T cells in tumors (**k**) from subcutaneous inoculation of wild-type Hepa1-6 cells, intraperitoneal injection of RIN1 (50 mg/kg) with subcutaneous inoculation of wild-type Hepa1-6 cells, and intraperitoneal injection of RIN1 with subcutaneous inoculation of Hepa1-6 cells overexpressing PD-L1 groups (*n* = 6). **l** Tumor growth curves from control-KD, *Rbpj*-KD, *Rbpj*-KD + Lv-*Cd274*, *Rbpj*-KD + L-kynurenine (30 mg/kg) groups (*n* = 6). **m**, **n** Inhibitory receptor (m, *n* = 4) and cytokine (**n**, *n* = 5) expression on CD8^+^ T cells from control-KD, *Rbpj*-KD, *Rbpj*-KD + Lv-*Cd274*, *Rbpj*-KD + L-kynurenine (30 mg/kg) groups. **o**, **p** Flow cytometry analysis of the expression of inhibitory receptors (**o**, *n* = 4) and cytokines (**p**, *n* = 6) from DMSO, intraperitoneal injection of RIN1 (50 mg/kg), intraperitoneal injection of RIN1 with anti-PD-1 (50 mg/kg)/PD-L1 (50 mg/kg) antibodies groups. Mean ± SD. Statistical significance determined by paired two-tailed *t*-test (**a**–**p**). *P < 0.05; **P < 0.01.

It is known that Notch pathway is involved in regulating the expression of PD-L1^19^, and we speculated that RBPJ may play an important role in this process. In the validation, RIN1 specifically inhibited PD-L1 expression in HCC cells (Figs. 6a-e, Supplementary Fig. 9a), but had no effect on PD-L2 and MHC-I molecules (Supplementary Fig. 9b, c). The expression of PD-L1 in HCC cells in nude mice was significantly lower than that in C57BL/6J mice (Fig. 6d, e). Previous studies have indicated that excessive secretion of IFN-γ, TNF-α, interleukin and other pro-inflammatory cytokines by T cells in the tumor microenvironment induce the expression of PD-L1 in tumor cells^14–16^. Therefore, the difference between the two might be due to the deficiency of proinflammatory cytokines caused by the absence of T cells in nude mice. To simulate the interaction between tumor cells and CD8^+^ T cells, we co-cultured RIN1-treated HCC cells (*HCC*^*RIN1*^) or *HCC*^*RIN1*^ overexpressing PD-L1 (*HCC*^*RIN1*^ + PD-L1) (Supplementary Fig. 10a, b) with patient infiltrating CD8^+^ T cells, respectively. The results showed that PD-L1 attenuated the positive regulatory effect of *HCC*^*RIN1*^ on CD8^+^ T cells (Fig. 6f–i, Supplementary Fig. 11a–d). In vivo experiments confirmed that PD-L1 also reduced the antitumor effect of RIN1, including increased tumor growth (Fig. 6j) and upregulated PD-1/TIM3 expression on CD8^+^ T cells (Fig. 6k, Supplementary Fig. 11e).

To confirm that RIN1 indeed improved the function of tumor-infiltrating CD8^+^ T cells by inhibiting PD-L1 expression and reducing L-kynurenine secretion, we performed in vivo recovery experiments. Overexpression of *Cd274* in Hepa1-6 cells (Supplementary Fig. 10c) or intraperitoneal injection of L-kynurenine also attenuated the ameliorating effect of *Rbpj*-KD on CD8^+^ T cells in vivo (Fig. 6l–n, Supplementary Fig. 11f, g). Knockdown of *Rbpj* in tumor-associated macrophages could inhibit its negative regulation of T cell proliferation^20^, therefore, we further explored whether the positive regulation of RIN1 on CD8^+^ T cells was related to tumor-associated macrophages. Depletion of macrophages (clodronate liposomes) in mice did not reduce the protective effect of RIN1 on CD8^+^ T cells (Supplementary Fig. 12a, b). In addition, RIN1 was able to enhance the efficacy of immune checkpoint inhibitors (anti-PD-1/PD-L1 antibodies) (Fig. 6o, p, Supplementary Fig. 11h, i). Taken together, these results suggested that RIN1 alleviated CD8^+^ T cell exhaustion by inhibiting PD-L1 expression and L-kynurenine synthesis in HCC cells.

### RIN1 inhibited the malignant behaviors of HCC cells by regulating glycolysis

**Fig. 7:**
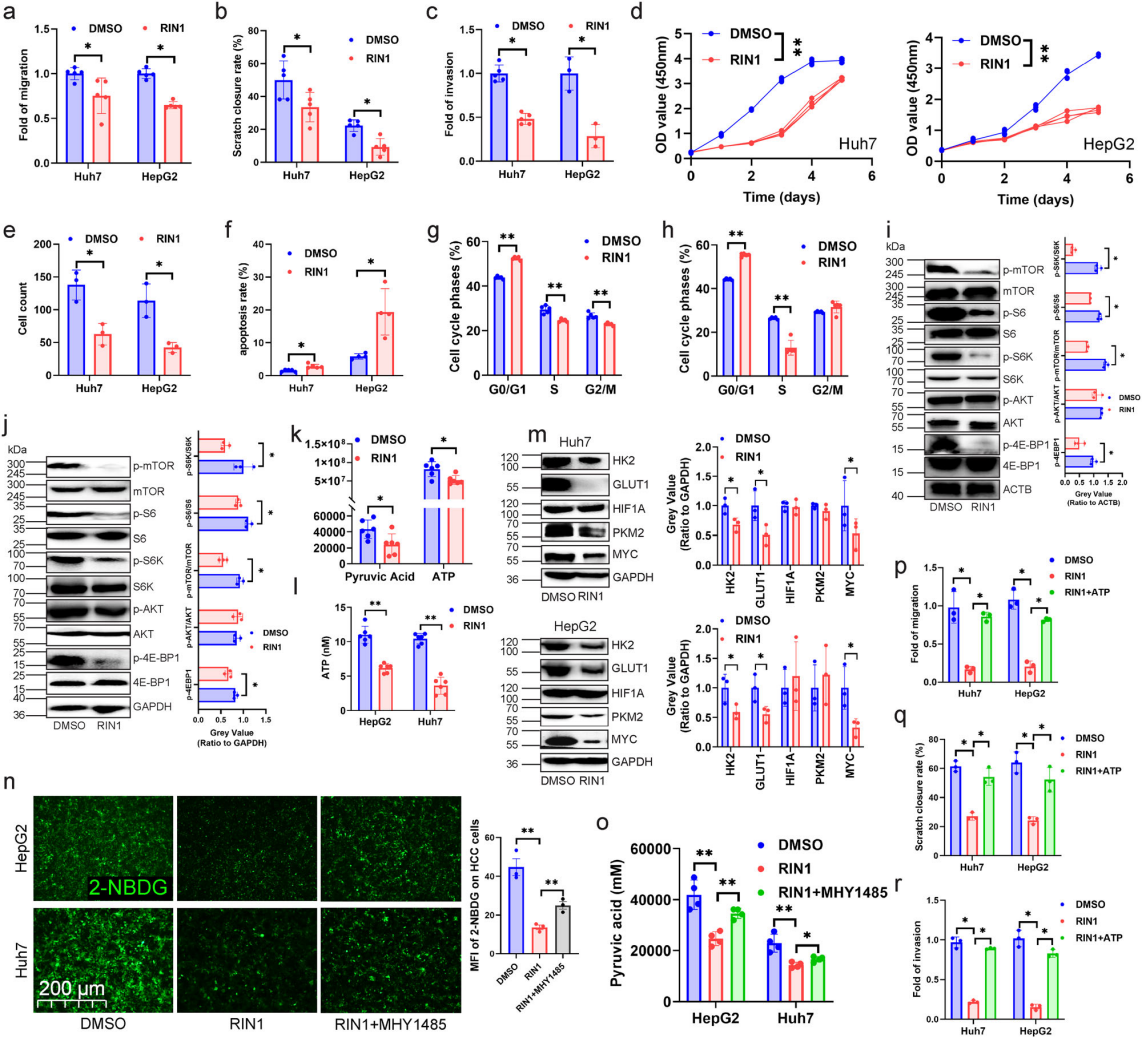
RIN1 inhibited glycolysis and malignancy of HCC cells by the mTOR pathway. **a**–**c** Transwell migration (**a**), wound healing (**b**) and transwell matrigel invasion assaies (**c**) were used to analyze cell migration and invasion in Huh7 and HepG2 cells (*n* = 5). **d** CCK-8 experiment was performed to examine cell viability of Huh7 and HepG2 cells (*n* = 3). **e** Colony forming assay evaluated colony forming capacity of Huh7 and HepG2 cells (*n* = 3). **f** Annexin-V APC/PI were used to examine apoptosis rate of Huh7 and HepG2 cells (*n* = 5). **g**, **h** PI/RNase were employed to detect cell cycle distribution of Huh7 (**g**) and HepG2 (**h**) cells (*n* = 5). **i**, **j** Immunoblot analysis of the protein expression of the mTOR pathway-related genes in Huh7 (**i**, *n* = 3) and HepG2 (j, *n* = 3) cells. **k** Untargeted metabolomics analysis demonstrating pyruvate and ATP content in Huh7 cells (*n* = 6). **l** ELISA was performed to detect ATP in HepG2 and Huh7 cells (*n* = 6). **m** Effect of RIN1 on the protein expression of the genes associated with the glycolytic in HepG2 and Huh7 cells (*n* = 3). **n**, **o** 2-NBDG fluorescence intensity (glucose uptake, **n**, *n* = 3) and pyruvate synthesis (**o**, *n* = 4) in Huh7 and HepG2 cells. **p**–**r** Transwell migration (**p**), wound healing (**q**) and transwell matrigel invasion assaies (**r**) were used to analyze cell migration and invasion of Huh7 and HepG2 cells (*n* = 3). Mean ± SD. Statistical significance determined by paired two-tailed *t*-test (**a**–**r**). *P < 0.05; **P < 0.01.

We next investigated the relationship between RBPJ expression and the malignancy of HCC cell itself, and found that RBPJ expression in HCC was higher than that in paired paracancerous tissues (Supplementary Fig. 13a, b), and RBPJ expression was higher in CNLC III + IV patients than in CNLC I + II patients (Supplementary Fig. 13c). Although RBPJ was not associated with patients’ HBV infection, tumor size, and liver cirrhosis (Supplementary Fig. 14), patients in RBPJ^high^ expression group exhibited shorter overall survival, disease-free survival outcomes, and progression free interval (Supplementary Fig. 13d), and higher pathological grade, stage (Supplementary Fig. 13e) than in RBPJ^low^ expression group. These results suggested that RBPJ was a gene that was highly expressed in HCC tissues and associated with poor prognosis. Experiments investigating the effect of RIN1 on the biological behaviors of HCC cells revealed that RIN1 inhibited the migration (Fig. 7a), scratch healing (Fig. 7b), invasion (Fig. 7c), proliferation (Fig. 7d), clone formation (Fig. 7e), apoptosis tolerance (Fig. 7f) and cell cycle (Fig. 7g, h) of HCC cells.

Using TCGA database to perform Gene Set Enrichment Analysis (GSEA) on the function of RBPJ in HCC, we found that “PI3K-AKT-MTOR-SIGNALING” was enriched in RBPJ^high^ expression group (Supplementary Fig. 15). Previous studies indicated that *Rbpj* knockout inhibited mTOR signaling in hepatocytes, as indicated by lower phosphorylation of *mTor* and *mTorc1* targets, p70 S6 kinase and 4E-BP1^17^. Cytological experiments confirmed that RIN1 significantly inhibited the phosphorylation levels of mTOR, S6K, S6, and 4E-BP1 in HCC cells, but did not affect AKT phosphorylation (Fig. 7i-j). In the recovery experiments, it was found that the inhibitory effect of RIN1 on migration, wound healing, invasion, proliferation, clone formation, apoptosis tolerance, and cell cycle of HCC cells could be attenuated by mTOR activator (MHY-1485) (Supplementary Fig. 16-17). Since mTOR pathway is involved in glucose metabolism^21^, we next investigated whether RIN1 affected the glycolysis in HCC. Both untargeted metabolomics analysis and ELISA indicated that RIN1 did inhibit the content of pyruvate and ATP in HCC cells (Fig. 7k-l). Although RIN1 did not affect PKM2 and HIF1A, it inhibited the expression of HK2, GLUT1 and MYC (Fig. 7m). In addition, MHY1485 was able to rescue the inhibitory effect of RIN1 on glucose uptake (Fig. 7n) and pyruvate synthesis (Fig. 7o) of Huh7 and HepG2 cells. When HCC cells were given ATP supplementation, the inhibitory effect of RIN1 on migration and invasion was attenuated (Fig. 7p-r). These results fully demonstrated that RIN1 inhibited glycolysis and malignant biological behaviors of HCC cells through mTOR pathway.

## Discussion

Various algorithms including CIBERSORT^22^ and TIMER^23^ for analyzing immune cell infiltration in tumor tissues are based on the expression of cell markers to score the abundance of infiltration, which have been widely used in analyzing the relationship between genes or prognostic signatures and immune cell infiltration. However, they could not characterize the genes at different steps of the process from “release of cancer cell antigens” to “killing of cancer cells”, nor could they identify hub genes that regulate the cancer-immune cycle. The weighted gene co-expression network constructed by us overcame this dilemma, which could identify highly correlated gene modules in HCC, determine the relationship between modules and seven steps of the cycle, search for hub genes that regulate different steps, and analyze the phenotype of hub genes. RBPJ was found to be related to the expression of PD-1 on CD8^+^ T cells, and the molecular mechanism of RIN1 regulating the killing ability of CD8^+^ T cells was elucidated.

Impaired function of tumor antigen-specific CD8^+^ T cells is one of the important reasons for the failure of immunotherapy. High expression of PD-L1 in tumor cells^24^, enhanced activity of tryptophan metabolizing enzymes^25^, upregulation of checkpoint genes in T cells^26^, alteration of intestinal flora^27^ and recruitment of immunosuppressive cells inhibit the activity of CD8^+^ T cells^28^. L-kynurenine is the main metabolite of tryptophan, and tryptophan 2,3-dioxygenase inhibitors can effectively reduce L-kynurenine synthesis, delay tumor growth and metastasis, which mechanistically involves aryl hydrocarbon receptor (AHR)^29^. AHR promotes Treg cell differentiation^18^, induces PD-1 expression on CD8^+^ T cells^18^, and upregulates CD39 expression on the surface of macrophages^30^, thereby inhibiting CD8^+^ T cell. Our study found that RIN1 effectively reduced L-kynurenine *via* TPH1 and IDO1 in addition to inhibiting PD-L1 expression in HCC cells. Its mechanism of alleviating CD8^+^ T cell exhaustion was unique and involved multiple pathways: (1) inhibiting the expression of transcription factors and receptors associated with exhaustion; (2) increasing the expression of genes encoding downstream of IFNAR1, enhancing CD8^+^ T cell response to IFNα/β; (3) improving the energy metabolism. These results indicated that RIN1 was a small molecule compound that enhanced the function of CD8^+^ T cells in two ways, and had promising prospects.

Active energy metabolism promotes the proliferation and metastasis of tumor cells and is considered as a malignant feature of tumor. Tumor cells use glycolysis to rapidly supply ATP for their own growth. Glycolysis inhibitors including STF-31 (a small molecule inhibitor of GLUT1)^31^, Fasentin (inhibition of GLUT1/GLUT4)^32^ and BAY-876 (an ATP inhibitor)^31^ have shown satisfactory results in the tumor-bearing mouse models. Similarly, in this study, we found that RIN1 inhibited glycolysis through mTOR pathway to reduce ATP synthesis and inhibit the growth, migration and invasion of HCC cells. This negative effect of RIN1 on HCC cells itself was also worthy of affirmation.

In conclusion, our results showed that compared with the common immune checkpoint inhibitors, RIN1 had the characteristics of inhibiting the development of HCC through multiple pathways. It not only inhibited the malignant biological behaviors of HCC cells, but also alleviated CD8^+^ T cell exhaustion. This study confirmed that RIN1 was a potential anti-HCC immunotherapy compound, but also provided a new idea for the combined strategy of targeting RBPJ treatment.

## Methods

### Ethics statement

This study protocol was reviewed and approved by the Ethics Committee of Fujian Medical University Union Hospital, approval number 2021KJCX008; and the Experimental Animal Ethics Committee of Fujian Medical University, approval number IACUC FJMU 2022-0015. The liver cancer specimens used in this study were surgically resected excess tissue, and the Ethics Committee of Fujian Medical University Union Hospital has agreed to waive the patients’ written informed consent.

### Animal

C57BL/6J female mice (6 weeks old, 18 ~ 20 g) were housed in a specific pathogen free environment with a 12/12 h day/night cycle. To construct a subcutaneous xenograft tumor model, 2 × 10^6^ Hepa1-6 cells were injected subcutaneously on the left side of mice, and the tumor volume was measured every 3 days using a vernier caliper. Tumor volume was calculated with the formula (length × width^2^/2). For induced HCC, at 8 weeks of age (six weeks after a single injection of diethylnitrosamine (DEN)), begin administration of carbon tetrachloride (CCl_4_) (0.2 ml/kg *i.p*.) twice a week for up to 14 additional weeks (dose volume: 15 mL/kg body weight). When the tumor volume reached 250 mm^3^, RIN1 50 mg/kg, Bevacizumab 100 mg/kg, *InVivo*Plus anti-mouse PD-1 50 mg/kg, *InVivo*Plus anti-mouse PD-L1 50 mg/kg, L-kynurenine 30 mg/kg and clodronate liposomes 1 mL/kg were intraperitoneally injected every 3 days for a total of seven times.

### Data sources and bbioinformatics analysis

We used Genomic Data Commons (GDC) download tool (https://portal.gdc.cancer.gov) from The Cancer Genome Atlas (TCGA, *n* = 371) to download the transcriptome data and clinical information of HCC. Fragments per Kilobase million format was used to calculate transcription spectra.

### Patient specimens

From 2019 to 2022, all HCC and paired para-cancerous tissues were obtained from Fujian Medical University Union Hospital and randomly used in this experiment. All patients were diagnosed with HCC based on tissue specimens. The patients had not undergone chemotherapy, radiotherapy, or other new adjuvant therapy prior to surgery.

### Cell culture

For the preparation of *HCC*^*RIN-sup*^, the supernatant was collected after 48 h of RIN1-treated HCC cells and the supernatant was collected by filtration through a 0.45 μm filter to remove cells and cell debris. CD8^+^ T cells were treated with cell-free supernatant for 48 h. The mouse HCC cell line Hepa1-6, human HCC cell lines HepG2 and Huh7, and HEK-293T were purchased from American type culture collection (ATCC). HEK-293T cells were utilized for lentiviral packaging. HepG2, Huh7, and Hepa1-6 were cultured in Dulbecco’s Modified Eagle Medium (DMEM) medium containing streptomycin, penicillin, 12% (v/v) fetal bovine serum (FBS) at 37°C containing 5% carbon dioxide (CO_2_). HepG2 and Huh7 were identified by STR (2020.07). HCC cells or CD8^+^ T cells were stimulated with RIN1 10 μM, Rodatristat 10 μM, IDO1-IN-18 10 μM, MHY-1485 10 μM, L-Kynurenine 200 nM and *HCC*^*RIN1-sup*^ for 48 h.

### Weighted Gene Co-expression Network analysis (WGCNA) and hub genes identification

“WGCNA”^33^ R package was employed to construct a weighted co-expression network to analyze the relationship between cancer-immune cycle and HCC gene expression data. Cancer-immune cycle score downloaded from Tracking Tumor Immunophenotype (TIP) database (http://biocc.hrbmu.edu.cn/TIP/) served as the clinical phenotype of the network. We chose fit β-value = 12 as cut off to build a scale-free network. HCC gene expression matrix was converted to a proximity matrix and then to a topology matrix. Genes were clustered using an average linkage hierarchical clustering method based on topological overlap. According to the standard of hybrid dynamic shearing tree, the minimum number of genes in each module was set to 50. The eigengenes of each module were calculated, and the cluster height was set to 0.25. Genes that could not be clustered into other modules were classified as gray module. According to the characteristic genes of each module, the correlation coefficient between each module and the cycle was calculated, then the module phenotype correlation heatmap was drawn.

### Calculation of immune cell infiltration and immunotherapy response

Single sample gene set enrichment analysis (ssGSEA) algorithm in gsva R package^34^ was performed to calculate the abundance of immune cell infiltration in HCC tissues based on the gene expression. ssGSEA was based on the specific genome of leukocytes, and HCC gene expression data was converted into the infiltrating abundance of immune cell populations, resulting in a ssGSEA score matrix, where the columns were the scores for each immune cell and the rows were patients’ IDs. “Estimate” R package^35^ calculated “ESTIMATEScore”, “StromalScore”, and “ImmuneScore” of each sample with built-in markers. Correlation coefficients > 0.5 and *P* value < 0.05 were selected as criteria for statistical significance. Tumor Immune Dysfunction and Exclusion (TIDE) (http://tide.dfci.harvard.edu/)^36^ and Submap algorithms^37^ (http://cloud.genepattern.org/gp) were employed to explore whether RBPJ could predicted the efficiency of a patient’s response to immunotherapy. TIDE algorithm was used to measure the TIDE score of each HCC specimen in TCGA database, and then Submap algorithm was performed to compare the difference in immunotherapy efficiency between RBPJ^high^ and RBPJ^low^ expression groups.

### Harvesting and processing of human liver samples

Fresh resected liver tissue samples were taken within 2 h to the laboratory to start tissue dissection and processing. First, tissue samples were thoroughly washed with phosphate buffered saline (PBS) to remove visible blood clots and to reduce blood leukocytes contamination. After tissue was minced using scalpels into ~3–5 mm diameter pieces and digested (1 mg/mL collagenase IV, 10 mg/mL Deoxyribonuclease-1 (DNase I), 10% FBS, RPMI 1640) at 37°C for 45 min using the gentle MACS Octo Dissociator with Heaters and continuous shaking. The enzymatic reaction was stopped by adding ethylenediaminetetraacetic acid (EDTA) 2 mM in PBS to a double volume of the sample. Afterward, the homogenate was filtered through a 100 mm cell strainer and centrifuged at 400 × g for 8 min at 4°C to pellet the cells and myelin. This was followed by myelin removal step by gradient centrifugation with 30% Percoll in PBS (1592 × g for 30 min at 4°C; without brakes during deceleration) using a 50 mL tube with a lid for a fixed angle rotor fitting in a centrifuge. After myelin (the top white layer) separation, the middle transparent layer without the bottom layer of red blood cells was collected and filtered once more through a 100 mm cell strainer. The single-cell suspension was washed in PBS and centrifuged at 400 × g for 8 min at 4°C to pellet the cells. Next, cells were ready for flow cytometry analysis or storing samples by freezing. For CD8^+^ T cell isolation, the human CD8 microbeads were used according to the manufacturer’s protocol, and cell purity was verified with FACSAria™ III, and sorted cells with a purity >95% were considered eligible specimens.

### Harvesting and processing of mouse liver samples

Mice were euthanized with CO_2_ and perfused with PBS through the left ventricle of the heart using a 25-G butterfly needle attached to a 50 mL syringe. The collected complete liver sample was dissected into ~1–3 mm diameter pieces using scissors and digested (0.4 mg/mL collagenase IV, 10 mg/mL DNase I, 10% FBS, RPMI 1640) at 37°C for 30 min applying continuous shaking. The enzymatic reaction was stopped by adding EDTA in PBS to a final concentration 5 mM. To homogenize the sample, it was repeatedly aspirated and ejected using a 5 mL syringe with a 20-G needle until a uniform homogenate was formed. Afterward, the homogenate was filtered through a 70 mm cell strainer and centrifuged at 400 × g for 8 min at 4°C to pellet the cells and myelin. This was followed by myelin removal step by gradient centrifugation with 30% Percoll in PBS (1592 × g for 30 min at 4°C; without brakes during deceleration) using a 50 mL tube with a lid for a fixed angle rotor fitting in a centrifuge. After myelin (the top white layer) separation, the middle transparent layer without the bottom layer of red blood cells was collected and filtered through a 70 mm cell strainer. The single-cell suspension was washed in PBS and centrifuged at 400 × g for 8 min at 4°C to pellet the cells. Cells were then ready for flow cytometry analysis. For CD8^+^ T cell isolation, the mouse CD8 microbeads were used according to the manufacturer’s protocol, and cell purity was verified with FACSAria™ III, and sorted cells with a purity >95% were considered eligible specimens.

### Plasmid, transfection and lentivirus production and infection

*Rbpj*-KD (shRNA sequences were commissioned to design and synthesized by Tsingke Biotechnology Co., Ltd., shRNA primers were shown in the supplementary material) was cloned into pLKO.1-mCherry-puro vector, the scrambled sequence from our previous publication served as a non-targeting control^38^. Open reading frames (ORF) of *Cd274* and PD-L1 (ORF sequences were commissioned to design and synthesized by Tsingke Biotechnology Co., Ltd.) were cloned into pLenti-mCherry-puro vector. The stable cell lines were obtained using lentiviral infection. psPAX2-puro, pMD2.G-puro, and pLenti-puro were co-transfected into HEK-293 cells using Lipo3000. After the medium was discarded, the viral particles were collected with RPMI1640 medium, and the viral particles were collected by filtration through a 0.45 μM filter. Polybrene was added to a final concentration of 10 μg/ml when cells were infected, cells were infected for 48 h, then replaced with fresh RPMI1640 medium and subsequent experiments were performed.

### Metal-isotope-tagged antibodies

Pre-conjugated antibodies to metal isotope were purchased from Fluidigm or commercial suppliers in purified form and conjugated in house using the Maxpar X8 chelating polymer kit according to the manufacturer’s instructions.

### Cell surface staining for mass cytometry

To avoid nonspecific binding of antibodies, the sample was incubated at 4°C for 15 min in Human TruStain FcX (Fc Receptor Blocking Solution) or TruStain FcX™ (anti-mouse CD16/32) Antibody. Without washing, the cells were spun down, resuspended in the antibody mixture in PBS, and incubated at 4°C for 30 min. To optimize antibody staining for chemokine receptors, the sample was incubated at 37°C for 10 min, and at 4°C followed 20 min. After staining the surface antibodies, we added Cell-ID Cisplatin to the sample for 3 min to discriminate viable/dead cells. Then, the sample was washed once in PBS and centri Cisplatin fuged to pellet the cells.

### Intracellular cytokine staining for mass cytometry

Cells were permeabilized using FOXP3 Fix/Perm Buffer Set according to the manufacturer’s instructions for 45 min at 4°C. Subsequently, the sample was washed once in Perm/Wash buffer and incubated in the antibody mixture in Perm/Wash buffer for 30 min at 4°C. The sample was washed once in Perm/Wash buffer and centrifuged to pellet the cells.

### Cell preparation and mass cytometry acquisition

After cell surface and intracellular antibody staining, the cells were incubated in 4% paraformaldehyde aqueous solution overnight. Prior to acquisition the cells were pelleted without washing and resuspended in up to 1 mL of diluted 1:3000 Cell-ID Intercalator-Ir + Maxpar Fix and Perm Buffer for 3 h. After the sample was washed twice in PBS and twice in ddH_2_O, diluted to 1.5 × 10^6^ cells/mL in ddH_2_O containing 10% EQ Four Element Calibration Beads and filtered through a 40 mm filter cap FACS tube. Samples were analyzed with a Helious CyTOF2. Quality control and tuning processes on the Helios CyTOF2 were performed following the guidelines for the daily instrument operation. Data were collected as fcs files.

### Removal of dead and dying cells

Leukocytes were collected and centrifuged at 300 × g for 5 min. Supernatants were removed and the cells were resuspended in 100 μL of dead cell-removal beads per 1 × 10^7^ cells as described by the manufacturer. The mixture was incubated at room temperature (RT) for 15 min and added to the MS column. The columns were then washed four times with binding buffer. Live cells were collected from the flow-through.

### Preprocessing of cytometry data

Raw mass cytometry data were normalized using the MATLAB version of the Normalizer tool^39^. Cells were assigned by manually gating on Event length and DNA (^191^Ir and ^193^Ir) channels, followed by the dead cell discrimination analyzing ^195^Pt expression using FlowJo Software. Doublets were excluded using Gaussian discrimination channels. Next, data were concatenated and de-barcoded using Boolean gating in FlowJo software. The normalized data containing living cells from every individual sample were manually exported from FlowJo Software and imported into R studio of R using the R packages “flowCore”^40^ and “flowWorkspaceData”^41^ (R Foundation for Statistical Computing). Before automated high-dimensional data analysis, the mass cytometry data were transformed with a cofactor in the range of 5 and 60 using an inverse hyperbolic sine function^42^. For flow cytometry data, the compensation matrix was corrected using FlowJo software. After live, single, CD3 positive and compensated cells were exported and imported into R Studio. Before automated high-dimensional data analysis, flow cytometry data were transformed using an inverse hyperbolic sine function with a cofactor in the range of between 300 and 600. Additionally, all cytometry data were normalized between 0 and 1 to the 99-999^th^ percentile of the merged sample in each batch.

### Automated population identification

To identify T cell populations accurately, we first carried out a step of FlowSOM clustering to generate a starting point of 100 nodes, on pre-processed and combined mass/or flow cytometry datasets^43,44^. This was then followed by expert-guided manual meta-clustering using parameters. The respective k-value was manually chosen (in the range of between 20 and 30); identified clusters were annotated and merged based on a similarity of antigen expression in order to uphold the biological relevance of the dataset. Manually-annotated clusters were used to calculate the relative frequencies of T cell populations. Heatmaps display median expression levels of all markers per merged population and plotted using the R package “pheatmap”. From mass cytometry datasets, we pre-selected major populations and performed additional FlowSOM analysis to identify smaller cell subsets. We calculated the median antigen expression among selected cell types using the R package “dplyr”. For data visualization, we applied various dimensionality reduction techniques. For a complex overview of the compartment, we used t-Distributed Stochastic Neighbor Embedding (t-SNE)^45^. To create a t-SNE of isolated T cells, we pooled equally proportioned 120,000 T cells from the datasets.

### Plate clone formation assay

The HepG2 and Huh7 cells were digested and then resuspended in serum-free medium, and the cells were seeded into a 6-well culture plate at a density of 1 × 10^3^ cells per well. Fourteen days later, the cells were continually cultured. Every 3 days, cells and clones were observed microscopically and sub-cultured. After colony formation was completed, the colonies formed by cells were photographed under a microscope and washed three times with PBS. Then, add 1 mL of crystal violet staining solution to each well and stain for 20 min. Finally, the six-well plate that formed the clones was scanned.

### Cell proliferation assay

Cell proliferation was tested using Cell Counting Kit 8. HepG2 and Huh7 cells (1000 cells per well plate) were plated into 96-well plates with RIN1 concentration of 10 nM for 48 h.

### Cell cycle detection

Flow cytometry was used to detect cell cycle of HCC cells. HepG2 and Huh7 cells (1  × 10^5^ cells per well plate) were plated into 6-well plates with RIN1 concentration of 10 nM for 48 h. The cells were fixed overnight with 70% ethanol. The cells were resuspended in 250 μL of PI/RNase at a concentration of 1 × 10^6^ cells/mL, mixed, and incubated for 20 min at 4 °C. Relative light units were detected by FACSCelesta™ Flow Cytometer within 1 h.

### Apoptosis detection

Flow cytometry was performed to examine apoptosis of HCC cells. HepG2 and Huh7 cells (1 × 10^5^ cells per well plate) were plated into 6-well plates with RIN1 concentration of 10 nM for 48 h. The cells were resuspended in 250 μL of binding buffer at a concentration of 1 × 10^6^ cells/mL, and then 5 μL of Annexin V-APC and PI were added, mixed, and incubated for 20 min at 4 °C. Relative light units were detected using FACSCelesta™ Flow Cytometer within 1 h.

### Invasion and migration assays

We used transwell plates to assay cell invasion and migration. In the migration assay, we starved the cells in a serum-free medium for 12 h at 37 °C with 5% CO_2_. We then added 700 µL of DMEM with 20% FBS to the lower well and 500 µL of serum-free medium, including 1 × 10^6^ cells to the upper transwell inserts. After culturing for 48 h at 37 °C with 5% CO_2_, we counted the number of cells that adhered to the lower surface of the insert membrane. We performed the invasion assay in the same way, except that the transwell insert membrane was coated with Matrigel.

### Cell scratch test assay

Once cultured, the cells reached close to 100% confluence, the cell monolayer was mechanically scratched. Scratch healing was observed at 0, 12, 24, and 48 h under a microscope at ×40 magnification (white light bright field). We used ImageJ software to calculate the migration area on the scratch, using the following formula: Scratch area rate (%) = 12, 24, and 48 h after migration scratch area/initial scratch area ×100%.

### Quantitation of glucose uptake

Cells were grown to 75% confluency on 96-well plates. The glucose analog 2-NBDG (0.15 mg/mL) were added to the growth medium and incubated for 10 min at 37 °C. Excess of 2-NBDG was removed by rinsing with PBS. Plates were sealed with optical film and photographs were taken with the EVOS FLoid imaging system.

### Quantification of pyruvic acid

Centrifugation at 15,000 rpm at 4 °C for 10 min, 20 μL of the supernatant was applied to a 96 well plate. Approximately 30 min after crushing, 43 μL of distilled water and 66 μL of 0.25 g/L DNPH in 1 M HCl were added to the supernatant in the 96 well plate and the plate was rolled gently for 10 min at 37 °C. After 10 min, 66 μL of 1.5 M NaOH was added. The absorbance at 515 nm was measured by the microplate reader. Standards were prepared from a 40 mM sodium pyruvate solution, ranging in concentration from 0.04 to 6 mM.

### Multiplex immunohistochemistry

Tissues were fixed with 4% paraformaldehyde overnight at 4 °C, dehydrated in different concentrations of ethanol and embedded in paraffin, and cut to 5 μm with a microtome. Sections were placed in Improved Citrate Antigen Retrieval Solution and microwaved for 30 min to achieve antigen retrieval. Sections were blocked with 5% Bovine serum albumin (BSA) for 1 h and incubated with primary antibody overnight. After three washes with PBS, sections were incubated with secondary antibodies. Photographs were taken with the Vectra3 Automated Quantitative Pathology Imaging system.

### Flow cytometry

To validate surface marker expression, the cells were directly stained with the indicated fluorochrome-conjugated antibodies for 30 min in Cell Staining Buffer at 4 °C and analyzed by flow cytometry. The cells were then washed in PBS two times, resuspended in Cell Staining Buffer and analyzed by flow cytometry. Samples were acquired and recorded in a FACSCelesta™ Flow Cytometer, and data were analyzed with FlowJo software. Gating strategies were provided in Supplementary Figs. 18–19.

### Western blot

Cells and tissues were lysed with RIPA Lysis Buffer, centrifuged at 12,000 rpm at 4°C for 30 min. Total protein concentration in supernatant was measured by BCA Protein Quantification Kit. Equal amounts of protein were loaded on 12% polyacrylamide gel, separated, and electroporated onto polyvinylidene difuoride membranes. Proteins on the membrane were blocked with QuickBlock™ Blocking Buffer for Western Blot for 15 min. Proteins were incubated with primary antibodies at 4°C overnight followed by secondary antibodies for 1 h at RT. Visualized with SuperSignal West Dura, imaged with ChemiDoc MP Imaging System.

### Real‑time quantitative PCR

Total RNA was extracted and purified with FastPure Plant Total RNA Isolation Kit and reverse transcription was employed using HiScript II Q Select RT SuperMix for qPCR. RT-qPCR was performed on Applied Biosystems 7500 and 7500 Fast Real-Time PCR Systems using ChamQ SYBR qPCR Master Mix. PCR cycling conditions were 95 °C for 30 s, followed by 40 cycles of 95 °C for 10 s, 63 °C for 10 s and 72 °C for 30 s. The cDNA melting curve was set as usual. Relative gene expression was calculated using 2^−ΔΔCT^ method, calibrated against GAPDH or beta Actin.

### Detection of L-kynurenine content

The content of L-kynurenine secreted by Huh7 and HepG2 cells was detected using Kynurenine ELISA kit. Dilute the standard. Add 40 uL of sample or standard to the well and incubate at 37 °C for 30 min. Wash three times with PBS, add 50 uL of enzyme-labeled antibody, incubate at RT for 30 min, add 50 uL of chromogenic solution and 50 uL of stop solution. The absorbance value of each sample at a wavelength of 450 nm was measured with Multiskan™ FC System.

### Detection of ATP content

ATP content was detected with ATP Assay Kit. Add 100 uL of ATP detection working solution to the well, leave it at RT for 5 min, and add 20 uL of sample or standard to the well. The relative light units of each sample were detected with Multiskan™ FC System.

### Transcriptome sequencing

Total RNA was extracted and purified with FastPure Plant Total RNA Isolation Kit according to the manufacturer’s protocol. RNA-seq libraries were constructed with VAHTS® mRNA-seq V3 Library Prep Kit for Illumina. Sequencing was performed using Illumina NovaSeq 6000 platform (provided by Novogene Co., Ltd.), and the sequencing depth of each sample was 6 G bases.

### Untargeted metabolomics by Liquid Chromatography (LC)-MS/MS

The metabolites in Huh7 cells were extracted. LC-MS/MS analysis was performed using Vanquish UHPLC system and Orbitrap Q Exactive HF-X mass spectrometer in both positive and negative modes (provided by Novogene Co., Ltd.).

### Single-cell RNA-Seq library preparation

Single-cell suspensions were prepared from biopsies as described above. Bulk population cells were directly subjected to the 10× mass genomics chip, targeting 10,000 simultaneously captured live events. Each cell was uniquely barcoded during the cDNA library generation by Single Cell 3′ Reagent Kits v2 per the manufacturer instructions and subsequently sequenced on an Illumina HiSeq 2500 at the CCHMC DNA Sequencing and Genotyping Core.

### Single-cell RNA-Seq data analysis

All single-cell RNA-Seq data were processed using Cell Ranger version 3.0.2 and the mm10 reference. The UMI count matrix was imported to Seurat^46^ for postprocessing and downstream analysis. For each sample, doublets were first filtered out using Scrublet^47^. Then, cells either with high mitochondrial reads, a low number of detected genes, or excess UMI counts were discarded. The UMI count matrix was normalized and scaled following the standard Seurat pipeline by adjusting for mitochondrial read counts and UMI counts. Preliminary clustering was performed using the top 20 principal components to detect major cell types first, and this information was projected onto UMAP^45^. Also, marker genes were called for cell type confirmation, detecting genes exclusively expressed in distinct populations. To do so, we performed a series of pairwise differential expression analyses against each of all the other cell clusters using the “FindMarkers” function in Seurat with the parameters log_2_FC > 0.25 and minimal proportion of cells expressing a gene (min.pct) >0.1, and we selected genes that were commonly highly expressed in each cluster. The proportion of each cell type was measured for each sample and was compared between the different disease statuses using a two-tailed *t*-test to examine the change by the groups. Pseudotime analysis was performed using Slingshot for all sample integrations to map each cell from different groups onto a common pseudotime axis, which corresponded to the mouse T cell differential trajectory^48^. Cell densities along the pseudotime were visualized and compared across the different statuses using a density plot, and a two-tailed *t*-test was performed to test the significance.

### Cleavage Under Targets and Tagmentation (CUT&Tag)

CUT&Tag was performed based on a protocol published by Kaya-Okur et.al.^49^. In brief, cells were harvested, counted (50,000 cells) and centrifuged for 3 min at 600 × g at RT. Cells were washed twice in 1.5 mL Wash Buffer (20 mM N-2-hydroxyethylpiperazine-N-2-ethane sulfonic acid (HEPES) pH 7.5; 150 mM NaCl; 0.5 mM Spermidine; 1 × Protease inhibitor cocktail, EDTA free). 10 μL concanavalin A coated magnetic beads were added per sample and incubated at RT for 15 min. The supernatant was removed and bead-bound cells were resuspended in 50 μL Dig-wash Buffer (20 mM HEPES pH 7.5; 150 mM NaCl; 0.5 mM Spermidine; 1 × Protease inhibitor cocktail; 0.05% Digitonin) containing 2 mM EDTA. The primary antibody of RBPJ was diluted 1:50 in 50 μL of Dig-Wash buffer and then incubated on a rotator overnight at 4 °C. The primary antibody was removed and an appropriate secondary antibody was diluted 1:100 in 100 μL of Dig-Wash buffer and cells were incubated at RT for 30 min. Cells were washed using 1 mL Dig-Wash buffer to remove unbound antibodies. A 1:200 dilution of pAG-Tn5 was prepared in Dig-300 Buffer (0.05% Digitonin, 20 mM HEPES, pH 7.5, 300 mM NaCl, 0.5 mM Spermidine, 1 × Protease inhibitor cocktail) and incubated at RT for 1 h. Cells were washed twice with 1 mL Dig-300 Buffer to remove unbound pAG-Tn5. Then, cells were resuspended in 50 μL Tagmentation buffer (10 mM MgCl_2_ in Dig-wash Buffer) and incubated at 37 °C for 1 h. Next, 1 μL of 10% sodium dodecyl sulfate (SDS) was added to 50 μL of sample and incubated at 55 °C for 10 min to stop tagmentation. To extract the DNA, 1.5 × Ampure XP beads were added to each tube. The final DNA products were eluted with 20 μL Millipore water and prepared to amplify libraries. PCR cycling conditions: 72 °C for 5 min; 98 °C for 30 s; 12 cycles of 98 °C for 10 s and 63 °C for 30 s; final extension at 72 °C for 1 min. The final libraries were purified by adding 1.1 × Ampure XP beads and eluted in 30 μL 10 mM Tris pH 8.0. The size distribution of libraries was determined by Agilent 4200 TapeStation analysis. Libraries were sequenced on Illumina Novaseq 6000 (150-bp paired ends).

### Statistics and reproducibility

All data analysis was done with GraphPad Prism V.8 or SPSS V.19, and data were presented as mean ± standard deviation (*χ* ± *s*). When comparing two samples, the paired two-tailed *t*-test was used if the data were normally distributed and the variances were homogeneous. If the data were normally distributed with unequal variance, Wilcoxon rank sum test was used. *χ*^2^-test was used to analyze the relationship between RBPJ and clinical indicators. Pearson analysis was employed to analyze the correlation between RBPJ and immune checkpoints or abundance of leukocytes. Kaplan–Meier analysis was performed for the survival analysis. Flow cytometry data was analyzed using FlowJo software V10. *P* < 0.05 was set as the criterion for statistical significance.

### Reporting summary

Further information on research design is available in the Nature Portfolio Reporting Summary linked to this article.

### Supplementary information

Supplementary Information

Description of Additional Supplementary Files

Supplementary Data 1

Supplementary Data 2

Reporting Summary

## Data Availability

Raw data of RNA-seq of patient HCC infiltrating CD8^+^ T cells treated with *Huh7*^*RIN1-sup*^ (PRJNA881457), transcriptome and CUT&Tag sequencing of infiltrating CD8^+^ T cells from mouse normal liver and primary HCC (PRJNA904833), single-cell RNA-seq (PRJNA880758) are available at the SRA repository. All source data underlying the graphs and charts presented in this study are shown in Supplementary Data 1. All blots accompanied by size markers in every figure panel are shown in Supplementary Data 2.
