# Author Correction: Targeted inhibition of RBPJ transcription complex alleviates the exhaustion of CD8^+^ T cells in hepatocellular carcinoma

**DOI:** 10.1038/s42003-023-05465-y

**Published:** 2023-10-26

**Authors:** Banglun Pan, Zengbin Wang, Xiaoxia Zhang, Shuling Shen, Xiaoling Ke, Jiacheng Qiu, Yuxin Yao, Xiaoxuan Wu, Xiaoqian Wang, Nanhong Tang

**Affiliations:** 1 Department of Hepatobiliary Surgery and Fujian Institute of Hepatobiliary Surgery, Fujian Medical University Union Hospital, Fuzhou, China; 2 Cancer Center of Fujian Medical University, Fujian Medical University Union Hospital, Fuzhou, China; 3 Key Laboratory of Ministry of Education for Gastrointestinal Cancer, Fujian Medical University, Fuzhou, China

**Keywords:** Hepatocellular carcinoma, Immunosuppression, Cancer microenvironment

Correction to: *Communications Biology* 10.1038/s42003-023-04521-x, published online 30 January 2023.

In the original version of the Supplementary Information, Supplementary Fig. 16e showed a duplicate image between the DMSO and RIN1 experimental treatments at 0 days. This error has now been corrected in the Supplementary Information, by replacing the image for the RIN1 treatment at 0 days. No changes have been made to the PDF or HTML versions of the article.

### Supplementary information

Updated Supplementary Information

