# Supplementary material for: Targeted inhibition of RBPJ transcription complex alleviates the exhaustion of CD8+ T cells in hepatocellular carcinoma
Source: Commun Biol. 2023 Jan 30;6:123. doi: 10.1038/s42003-023-04521-x (PMC9887061; doi:10.1038/s42003-023-04521-x)
Supplement: Supplementary file 1 — Supplementary Information [file 42003_2023_4521_MOESM1_ESM.pdf]

Supplemental Information for

Targeted inhibition of RBPJ transcription complex alleviates the exhaustion of CD8$^+$ T cells in hepatocellular carcinoma

Banglun Pan, Zengbin Wang, Xiaoxia Zhang, Shuling Shen, Xiaoling Ke, Jiacheng Qiu, Yuxin Yao, Xiaoxuan Wu, Xiaoqian Wang, Nanhong Tang

Table of contents

Supplemental Figures.. 2
Supplemental Table 19
Supplemental Materials.. 21

Supplemental Figures

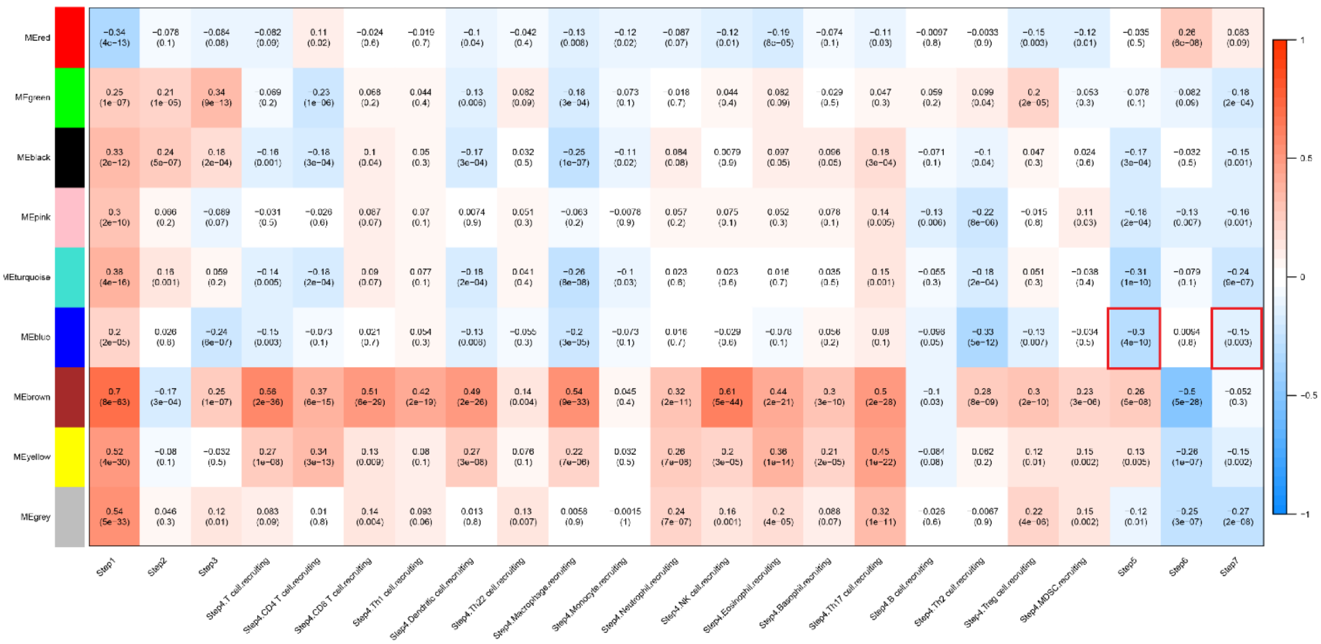

step1: Release of cancer cell antigens step2: Cancer antigens presentation step3: Priming and activation step4: Trafficking of immune cells to tumors step5: Infiltration of immune cells into tumors step6: Recognition of cancer cells by T cells step7: Killing of cancer cells

Supplementary Figure 1. Heatmap showing the correlation of each cluster with cancer-immunity cycle in TCGA-LIHC database. Red indicated a positive correlation, while blue indicated a negative correlation (*n* = 371).

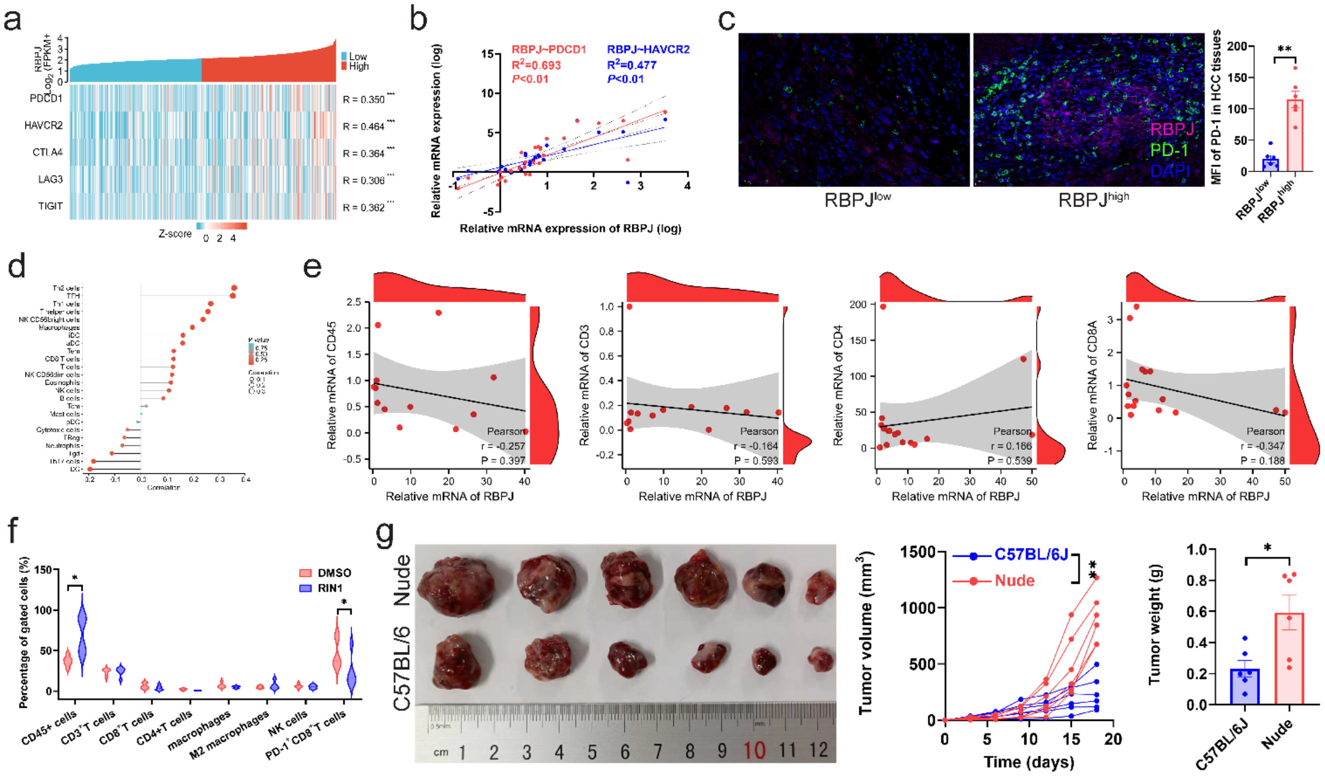

Supplementary Figure 2. RBPJ was an immunosuppressive target in HCC. a-b TCGA database (a, *n* = 371) and HCC specimens from patients (b, *n* = 28) were employed to examine the correlation of the expression of inhibitory receptors with RBPJ. **c** Immunohistochemistry showing the expression of RBPJ and PD-1 in HCC from patients (*n* = 6). Green represented PD-1, purple represented RBPJ, and blue represented DAPI. **d** Correlation of the abundance of 24 kinds of leukocytes with RBPJ expression in TCGA database (*n* = 371). **e** Correlation of the abundance of leukocytes with RBPJ expression in HCC from patients (*n* = 16). Leukocytes were represented by different biomarkers: CD45~leukocytes, CD3~T cells, CD4~CD4$^+$ T cells, CD8A~CD8$^+$ T cells. **f** Effect of RIN1 (50 mg/kg, *i.v.*) on leukocyte infiltration in mouse subcutaneous tumor (*n* = 6). **g** Subcutaneous HCC xenografts from C57BL/6J mice and nude mice treated with RIN1 (50 mg/kg, *i.v.*), including graphs of subcutaneous tumors, growth curves and weight (*n* = 6). Mean ± SD. Statistical significance determined by Pearson's rank correlation coefficient (a-b, d-e) and paired two-tailed *t*-test (c, f-g). *P < 0.05; **P < 0.01.

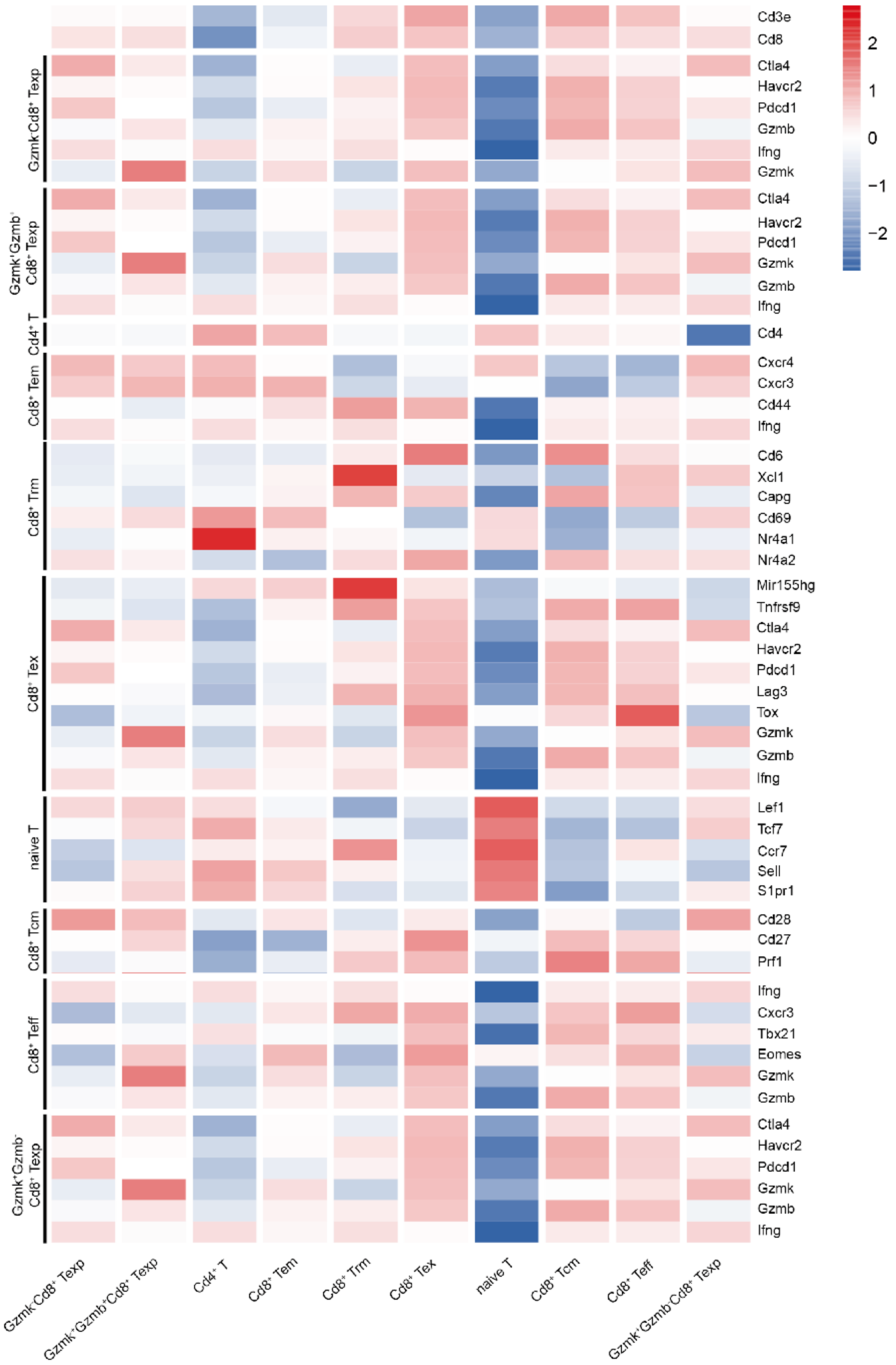

Supplementary Figure 3. Heatmap from single-cell RNA-seq showing the expression of indicated marker genes in different T cell subpopulations from C57BL/6J with HCC (*n* = 5).

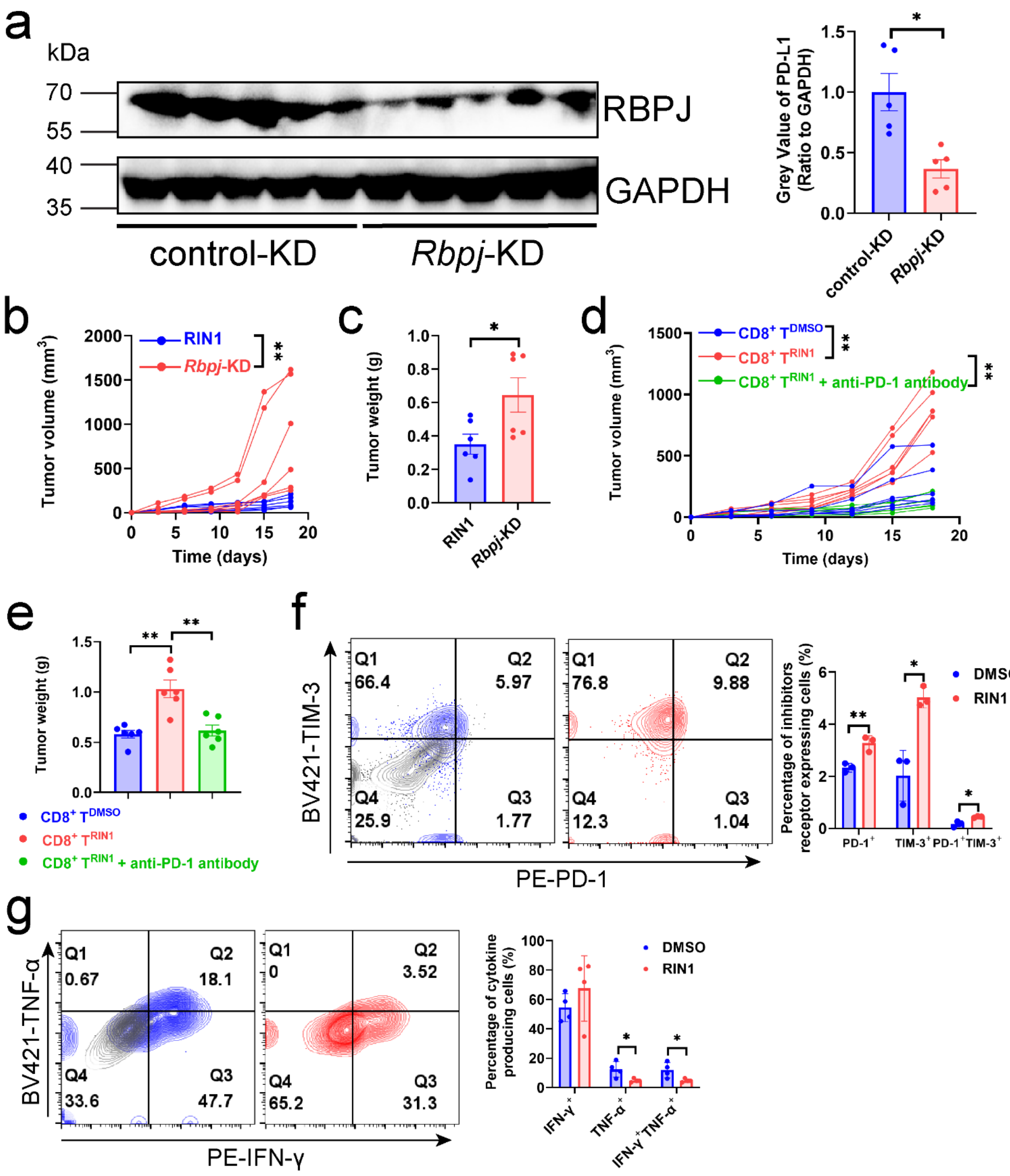

Supplementary Figure 4. RIN1 treatment *in vitro* did not simulate the immunopotentiation of *Rbpj* knockdown in CD8+ T cells. a Western blot demonstrating the efficiency of *Rbpj* knockdown in Hepa1-6 cells (*n* = 5). **b-c** Growth curves (b) and weight plot (c) showing the effects of RIN1 (50 mg/kg, *i.v.*) and *Rbpj* knockdown in Hepa1-6 cells on the growth of subcutaneous tumors (*n* = 6). **d-e** RIN1-treated CD8+ T cells *in vitro* (CD8+ T^{RIN1}, 10 μM) were adopted into HCC mice (*i.v.*), and the mice were treated with anti-PD-1 antibody (50 mg/kg, *i.v.*).

Growth curves (d) and weight plot (e) demonstrating the immunomodulatory effects of CD8$^+$ T^{RIN1} on subcutaneous tumor growth (*n* = 6). **f-g** Inhibitor receptor (f) and cytokine (g) expression of patient HCC infiltrating CD8$^+$ T cells treated with DMSO and RIN1 (10 μM) *in vitro* (*n* = 3). Gray indicated isotype control. Mean ± SD. Statistical significance determined by paired two-tailed *t*-test (a-g). **P* < 0.05; ***P* < 0.01.

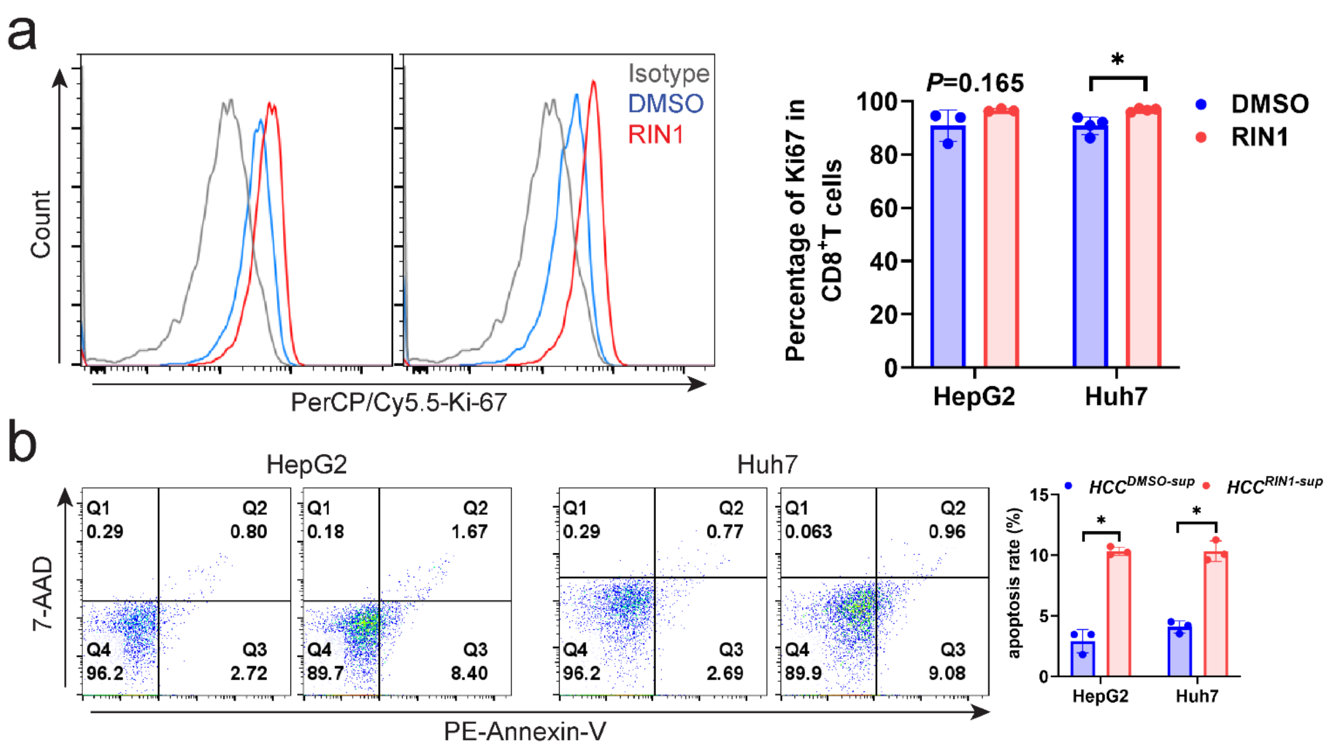

Supplementary Figure 5. Effects of *HCC$^{RIN1\text{-}sup}$* on proliferation and cytotoxicity of patient HCC infiltrating CD8$^+$ T cells. a Ki-67 expression of CD8$^+$ T cells from *HCC$^{DMSO\text{-}sup}$* and *HCC$^{RIN1\text{-}sup}$* groups (*n* = 4). **b** Killing of CD8$^+$ T cells against HepG2 and Huh7 cells (*n* = 3). Mean ± SD. Statistical significance determined by paired two-tailed *t*-test (a-b). **P* < 0.05.

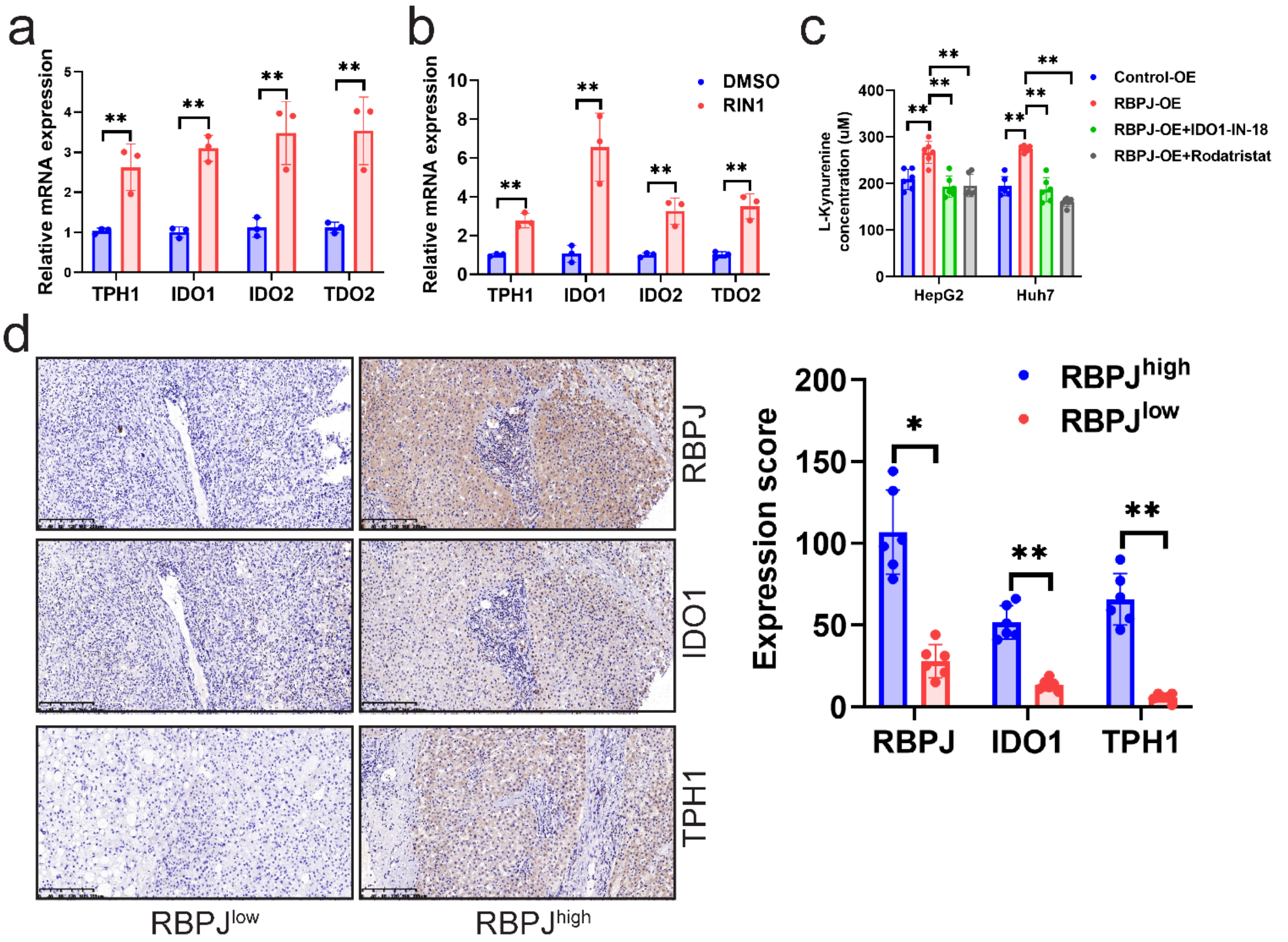

Supplementary Figure 6. RBPJ regulated the protein expression of IDO1 and TPH1 from the translational level rather than the transcriptional level. a-b RT-qPCR showing mRNA expression of TPH1, IDO1, IDO2, and TDO2 in HepG2 (a) and Huh7 (b) cells under RIN1 (10 μM) (*n* = 3). c ELISA analysis of L-kynurenine secretion by HepG2 and Huh7 cells from control-OE, RBPJ-OE, RBPJ-OE+IDO1-IN-18 (10μM), and RBPJ-OE+Rodatristat (10μM) groups (*n* = 6). **d** Immunohistochemistry demonstrating the protein expression of RBPJ, IDO1 and TPH1 in HCC (*n* = 6). Mean ± SD. Statistical significance determined by paired two-tailed *t*-test (a-c). **P* < 0.05; ***P* < 0.01.

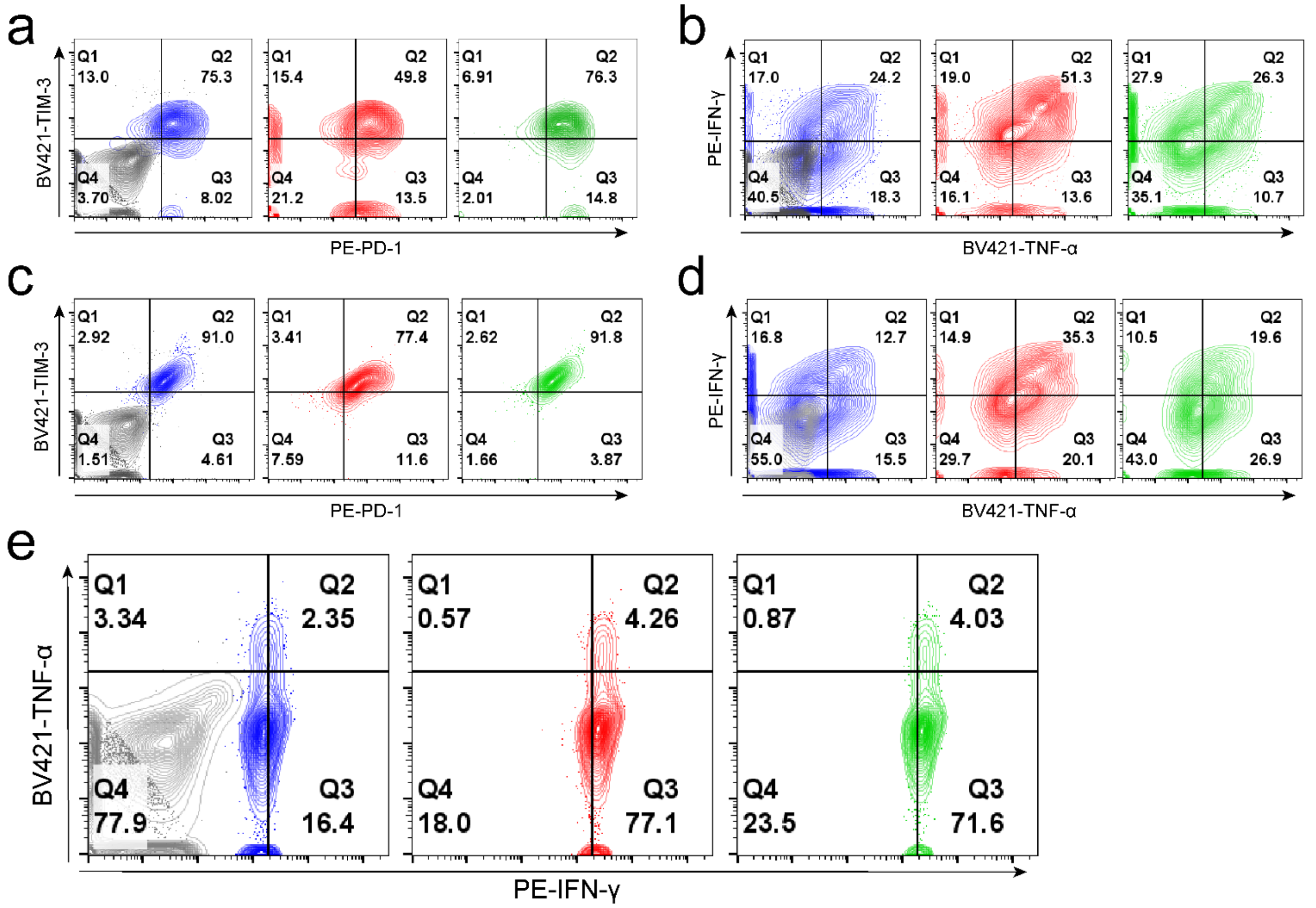

Supplementary Figure 7. RIN1 improved CD8^{+} T cell function by inhibiting tryptophan metabolism. a-d Inhibitory receptor (a, c) and cytokine (b, d) expression on patient HCC infiltrating CD8^{+} T cells from control (blue), *HCC$^{RIN1\text{-}sup}$* (red), and *HCC$^{RIN1\text{-}sup}$* with L-kynurenine (30 mg/kg) (green) groups, *HCC$^{RIN1\text{-}sup}$* included *Huh7$^{RIN1\text{-}sup}$* (a-b) and *HepG2$^{RIN1\text{-}sup}$* (c-d) (n = 4). Gray indicated isotype control. **e** Cytokine expression on CD8^{+} T cells in tumors from control (blue), intraperitoneal injection of RIN1 (50 mg/kg) (red), and intraperitoneal injection of RIN1 with L-kynurenine (30 mg/kg) (green) groups (n = 6). Gray indicated isotype control.

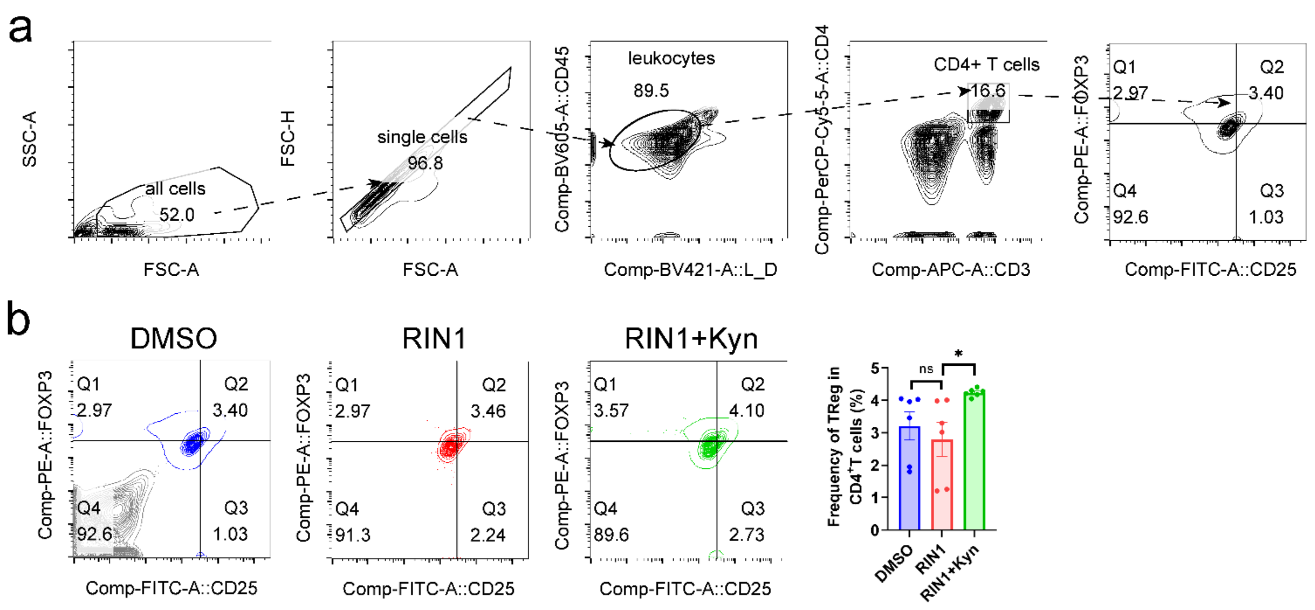

Supplementary Figure 8. RIN1 did not inhibit the infiltration of Treg in HCC. a Gating strategy. **b** Effect of RIN1 (50 mg/kg) and L-kynurenine (30 mg/kg) on the proportion of mouse HCC infiltrating Tregs was analyzed by flow cytometry (*n* = 6). Gray indicated isotype control. Mean ± SD. Statistical significance determined by paired two-tailed *t*-test (b). **P* < 0.05.

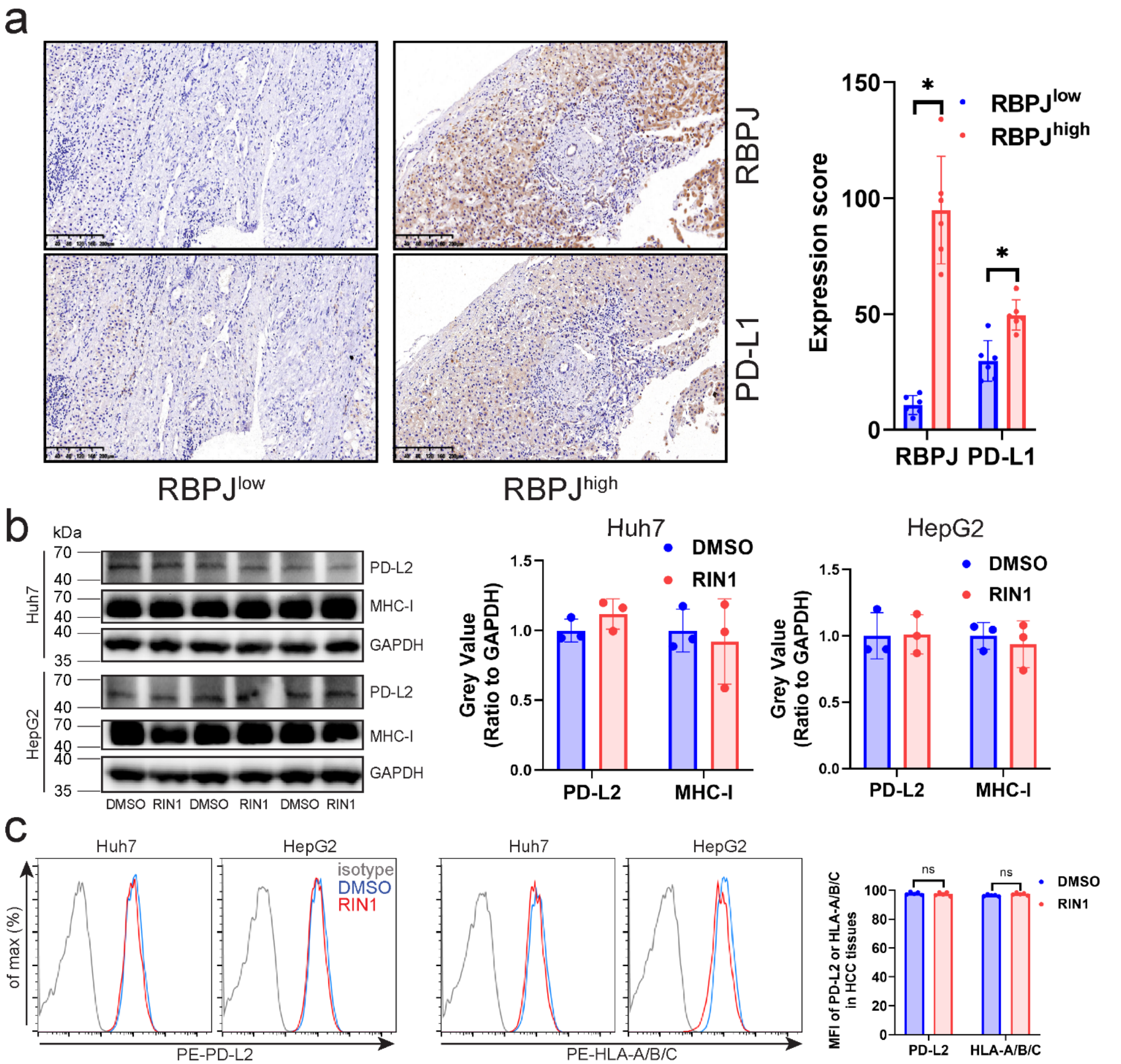

Supplementary Figure 9. RIN1 specifically inhibited PD-L1 protein expression in HCC. a Immunohistochemistry demonstrating the protein expression of RBPJ, PD-L1 in patient HCC tissues (*n* = 6). **b** Immunoblot showing PD-L2 and MHC-I molecules protein expression in Huh7 and HepG2 cells (*n* = 3). **c** Flow cytometry analysis of PD-L2 and HLA-A/B/C protein expression on the surface of Huh7 and HepG2 cells (*n* = 5). Mean ± SD. Statistical significance determined by paired two-tailed *t*-test (a-c).

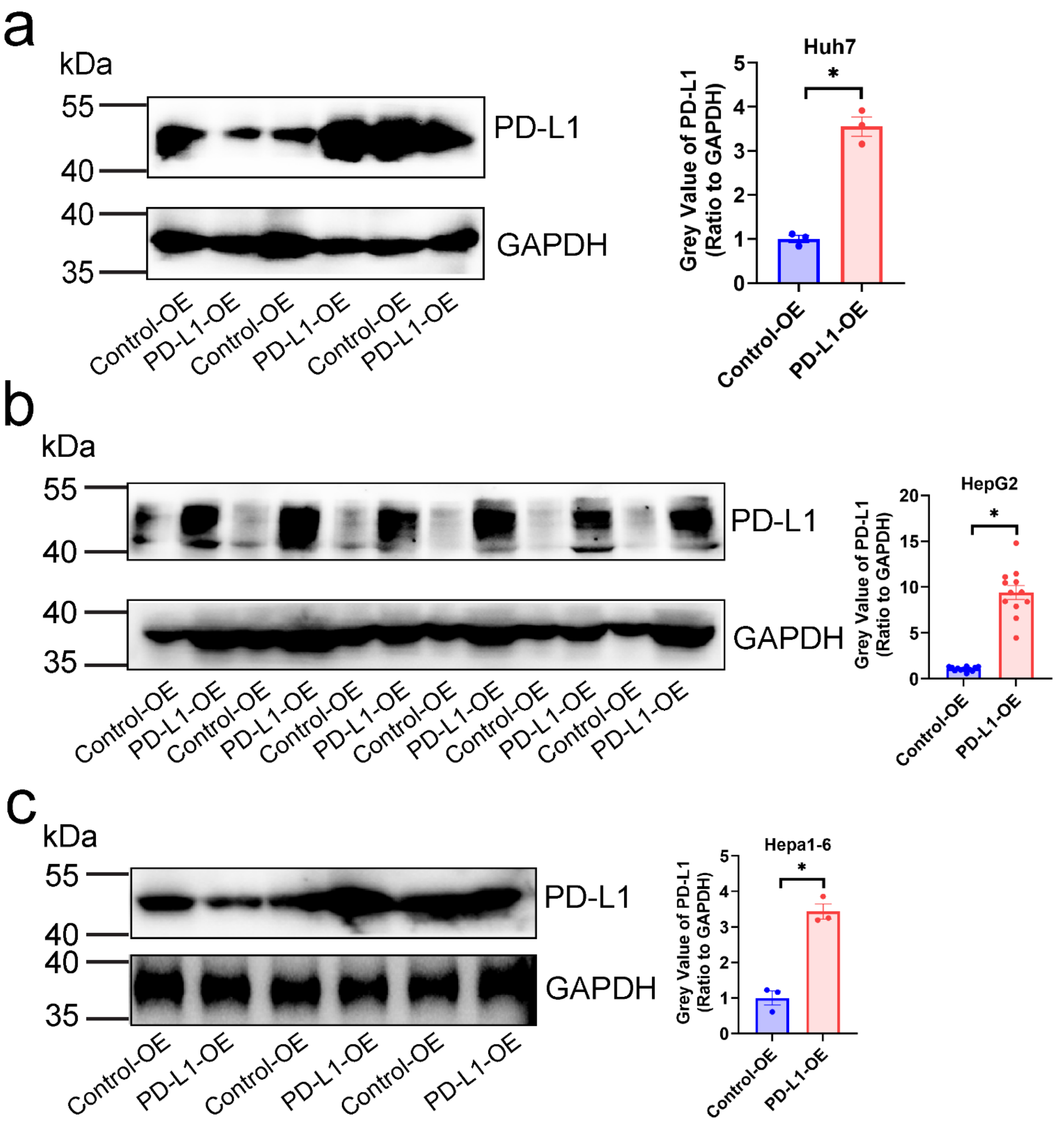

Supplementary Figure 10. PD-L1 was forced to be expressed ectopic on HCC cell lines. a-c Western blot demonstrated the expression of PD-L1 in Huh7 (a), HepG2 (b), and Hepa1-6 (c) cells ($n = 3$). Mean ± SD. Statistical significance determined by paired two-tailed t-test (a-c). *$P < 0.05$.

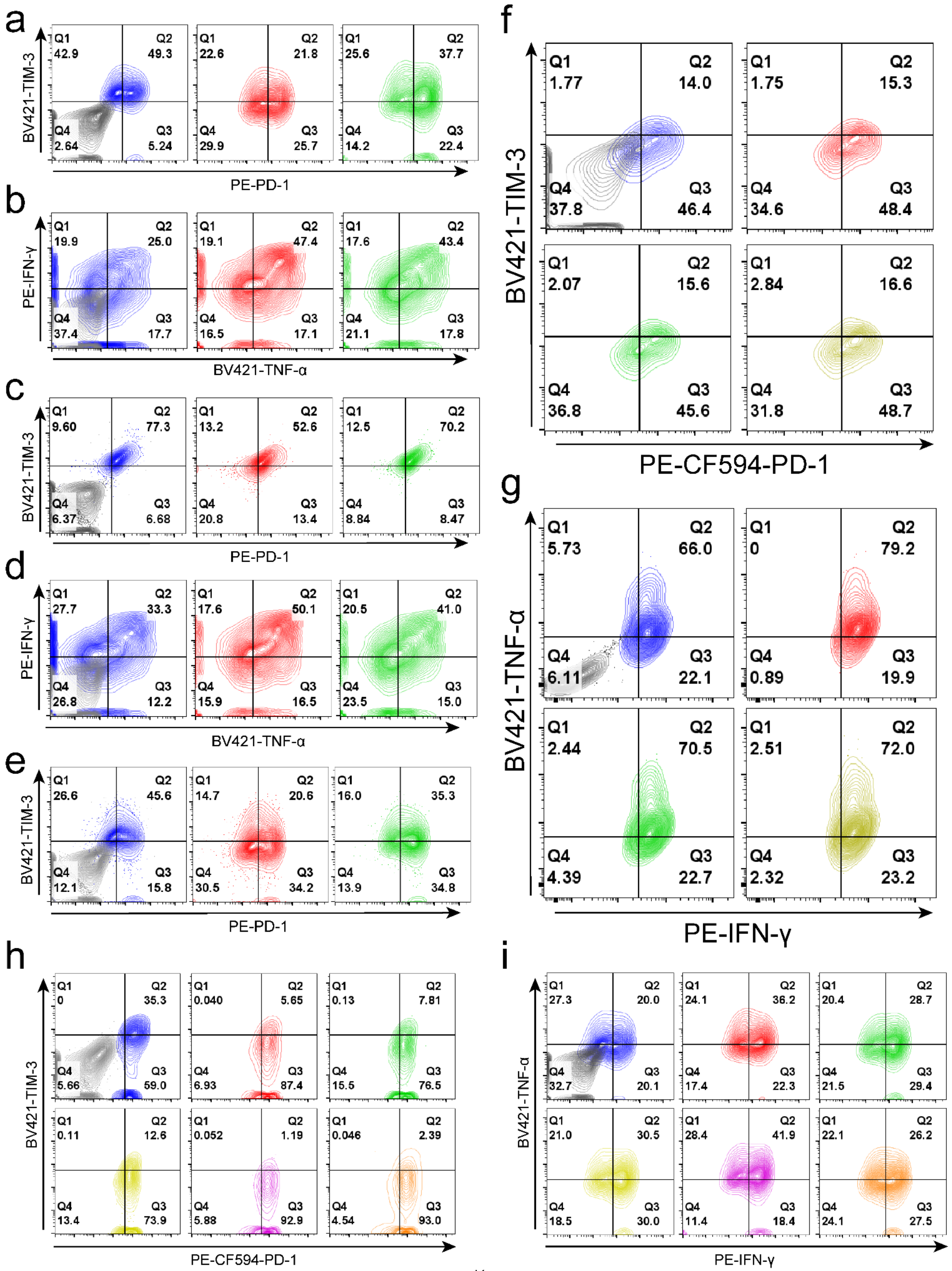
a
BV421-TIM-3
PE-PD-1
Q1 42.9 Q2 49.3 Q4 2.64 Q3 5.24
Q1 22.6 Q2 21.8 Q4 29.9 Q3 25.7
Q1 25.6 Q2 37.7 Q4 14.2 Q3 22.4
b
PE-IFN-γ
BV421-TNF-α
Q1 19.9 Q2 25.0 Q4 37.4 Q3 17.7
Q1 19.1 Q2 47.4 Q4 16.5 Q3 17.1
Q1 17.6 Q2 43.4 Q4 21.1 Q3 17.8
c
BV421-TIM-3
PE-PD-1
Q1 9.60 Q2 77.3 Q4 6.37 Q3 6.68
Q1 13.2 Q2 52.6 Q4 20.8 Q3 13.4
Q1 12.5 Q2 70.2 Q4 8.84 Q3 8.47
d
PE-IFN-γ
BV421-TNF-α
Q1 27.7 Q2 33.3 Q4 26.8 Q3 12.2
Q1 17.6 Q2 50.1 Q4 15.9 Q3 16.5
Q1 20.5 Q2 41.0 Q4 23.5 Q3 15.0
e
BV421-TIM-3
PE-PD-1
Q1 26.6 Q2 45.6 Q4 12.1 Q3 15.8
Q1 14.7 Q2 20.6 Q4 30.5 Q3 34.2
Q1 16.0 Q2 35.3 Q4 13.9 Q3 34.8
f
BV421-TIM-3
PE-CF594-PD-1
Q1 1.77 Q2 14.0 Q4 37.8 Q3 46.4
Q1 1.75 Q2 15.3 Q4 34.6 Q3 48.4
Q1 2.07 Q2 15.6 Q4 36.8 Q3 45.6
Q1 2.84 Q2 16.6 Q4 31.8 Q3 48.7
g
BV421-TNF-α
PE-IFN-γ
Q1 5.73 Q2 66.0 Q4 6.11 Q3 22.1
Q1 0 Q2 79.2 Q4 0.89 Q3 19.9
Q1 2.44 Q2 70.5 Q4 4.39 Q3 22.7
Q1 2.51 Q2 72.0 Q4 2.32 Q3 23.2
h
BV421-TIM-3
PE-CF594-PD-1
Q1 0 Q2 35.3 Q4 5.66 Q3 59.0
Q1 0.040 Q2 5.65 Q4 6.93 Q3 87.4
Q1 0.13 Q2 7.81 Q4 15.5 Q3 76.5
Q1 0.11 Q2 12.6 Q4 13.4 Q3 73.9
Q1 0.052 Q2 1.19 Q4 5.88 Q3 92.9
Q1 0.046 Q2 2.39 Q4 4.54 Q3 93.0
i
BV421-TNF-α
PE-IFN-γ
Q1 27.3 Q2 20.0 Q4 32.7 Q3 20.1
Q1 24.1 Q2 36.2 Q4 17.4 Q3 22.3
Q1 20.4 Q2 28.7 Q4 21.5 Q3 29.4
Q1 21.0 Q2 30.5 Q4 18.5 Q3 30.0
Q1 28.4 Q2 41.9 Q4 11.4 Q3 18.4
Q1 22.1 Q2 26.2 Q4 24.1 Q3 27.5

Supplementary Figure 11. RIN1 improved CD8^{+} T cell function by inhibiting L-kynurenine synthesis and PD-L1 expression. a-d Inhibitory receptor (a, c) and cytokine (b, d) expression on CD8^{+} T cells from control (blue), *HCC^{RIN1}* (red), and *HCC^{RIN1}*+Lv-PD-L1 (green) groups, *HCC^{RIN1}* included *Huh7^{RIN1}* (a-b) and *HepG2^{RIN1}* (c-d) (n = 4). Gray indicated isotype control. **e** Inhibitory receptor expression on CD8^{+} T cells in tumors from subcutaneous inoculation of wild-type Hepa1-6 cells (blue), intraperitoneal injection of RIN1 (50mg/kg) with subcutaneous inoculation of wild-type Hepa1-6 cells (red), and intraperitoneal injection of RIN1 with subcutaneous inoculation of Hepa1-6 cells overexpressing PD-L1 groups (green) (n = 6). Gray indicated isotype control. **f-g** Inhibitory receptor (f) and cytokine (g) expression on CD8^{+} T cells from control-KD (blue), *Rbpj*-KD (red), *Rbpj*-KD+Lv-*Cd274* (green), *Rbpj*-KD+L-kynurenine (30 mg/kg) (yellow) groups (n = 6). **h-i** Flow cytometry analysis of the expression of inhibitory receptors (h, n = 4) and cytokines (i, n = 6) from DMSO (blue), intraperitoneal injection of RIN1 (50mg/kg) (red), intraperitoneal injection of anti-PD-1 (50 mg/kg) (green)/PD-L1 (50 mg/kg) (yellow) antibodies, intraperitoneal injection of RIN1 with anti-PD-1 (purple)/PD-L1 (orange) antibodies groups. Gray indicated isotype control.

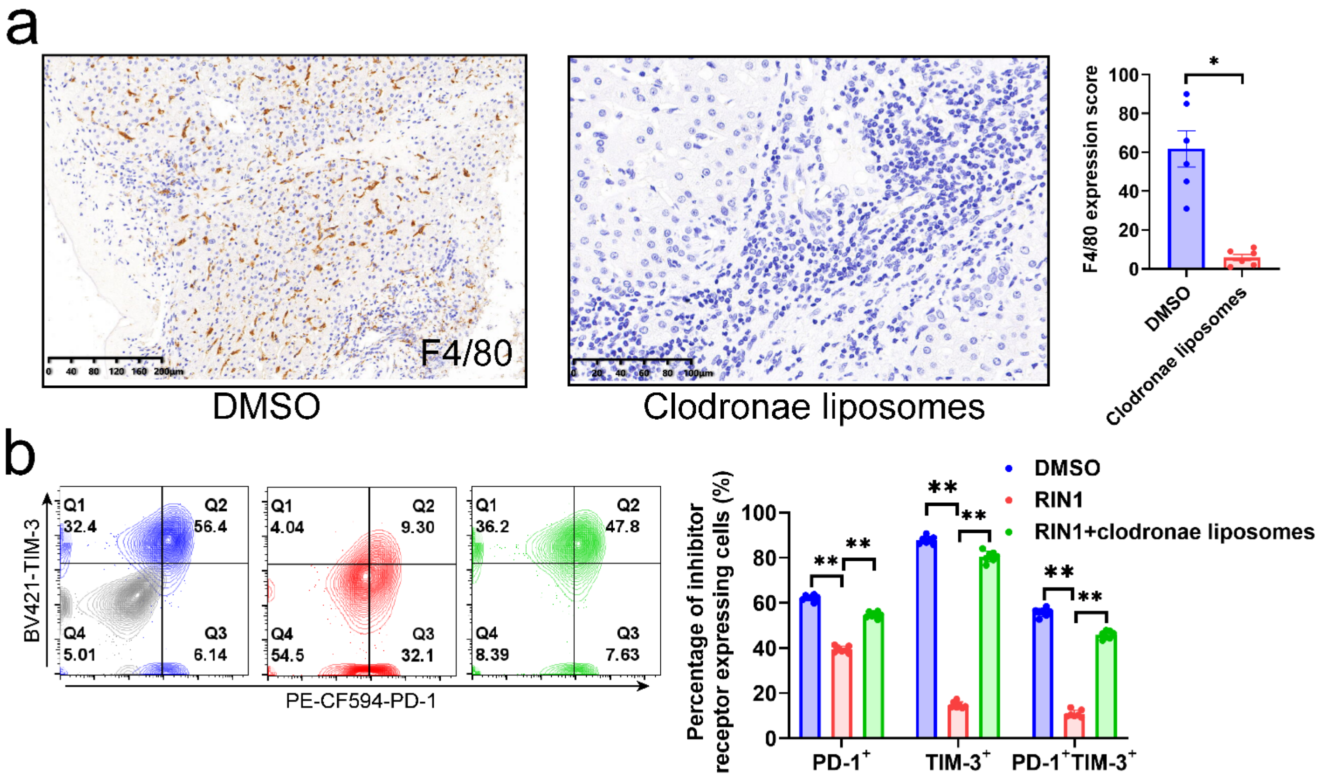

Supplementary Figure 12. The positive regulatory effect of RIN1 on mouse HCC-infiltrating CD8^{+} T cells was independent of macrophages. a Immunohistochemistry showed the expression of F4/80 in mouse HCC tissues (n = 6). **b** Inhibitory receptor expression on mouse HCC infiltrating CD8^{+} T cells from DMSO, RIN1 (50 mg/kg), RIN1+clodronae liposomes (1 mL/kg) groups (n = 5). Gray indicated isotype control. Mean ± SD. Statistical significance determined by paired two-tailed t-test (a-b). *P < 0.05; **P < 0.01.

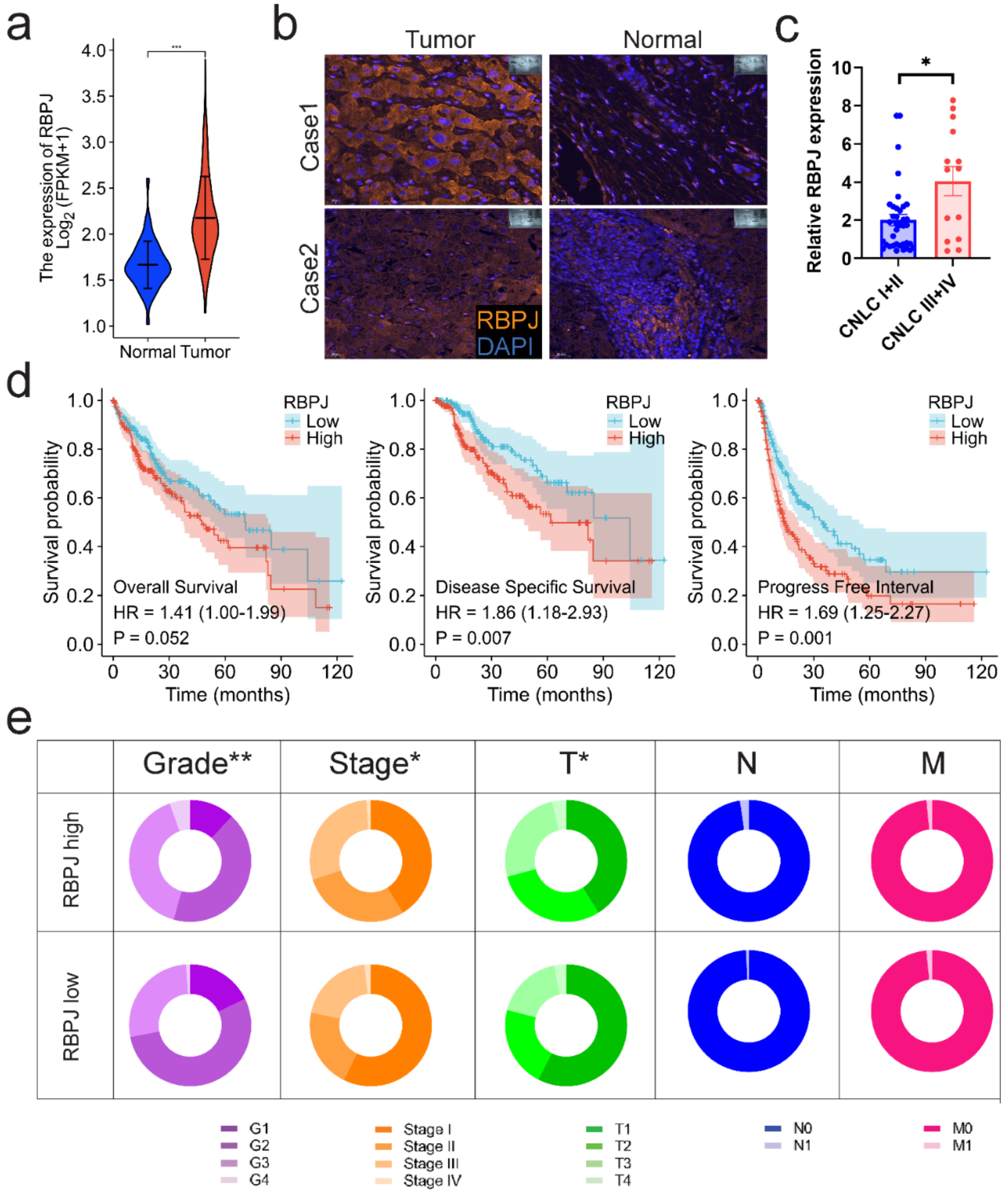

Supplementary Figure 13. RBPJ was highly expressed in HCC and associated with poor prognosis. a-b HCC gene expression data from TCGA database (normal, *n* = 50; tumor, *n* = 371) (a) and patient HCC tissues (normal, *n* = 6; tumor, *n* = 6) (b) were used to analyze the expression of RBPJ in HCC and paired para-cancerous tissues. **c** RT-qPCR analysis of mRNA expression of RBPJ in different China liver cancer staging (CNLC) grade. CNLC I+II was divided into low-grade group (*n* = 37), and CNLC III+IV was divided into high-grade group (*n* = 14). **d** Kaplan-Meier curves showing overall survival, disease specific survival, and progress free interval between RBPJ^{high} and RBPJ^{low} expression groups (*n* = 371). **e** Circular pie chart demonstrating the proportion difference of clinical indices (including pathological grade, stage, tumor, node, and metastasis) from RBPJ^{high} and RBPJ^{low} expression groups in TCGA database (*n* = 371). Mean ± SD. Statistical significance determined by paired two-tailed *t*-test (a, c), log-rank test (d), and χ2 test (e). **P* < 0.05; ***P* < 0.01; ****P* < 0.001.

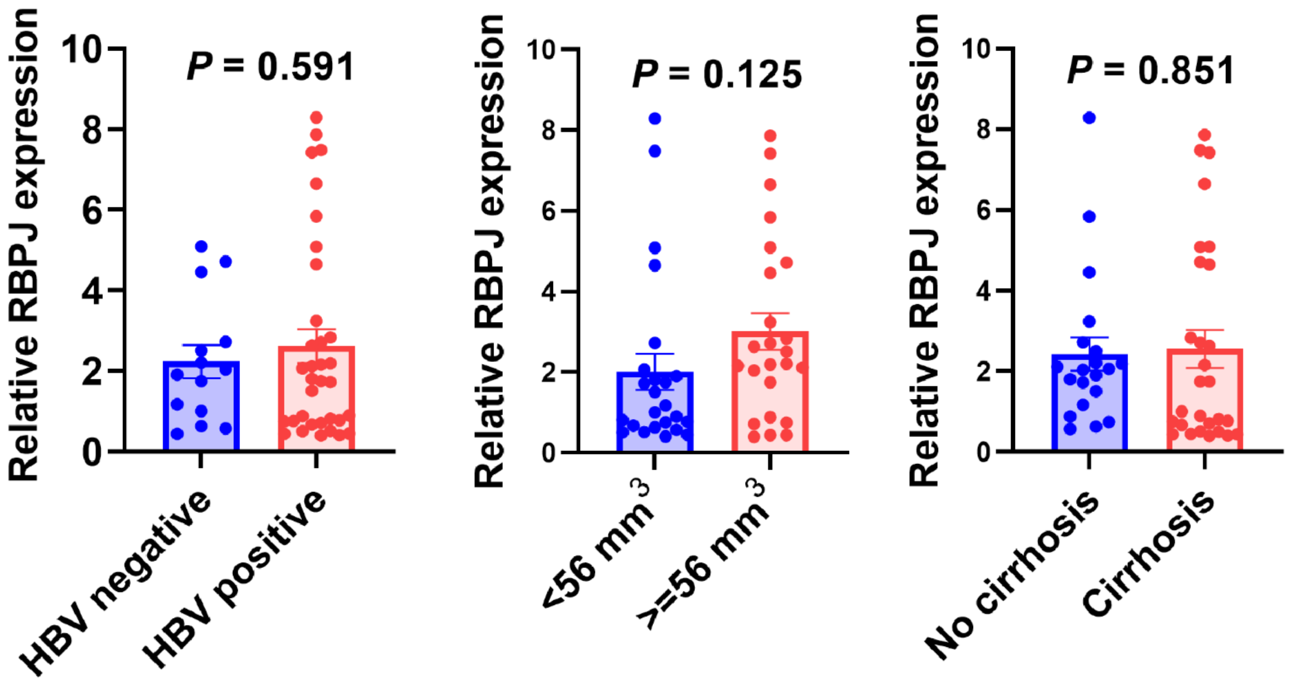

Supplementary Figure 14. RT-qPCR analysis of the relationship between RBPJ mRNA expression and clinical parameters in patient HCC tissues. Clinical parameters included HBV infection (HBV negative, n = 14; HBV positive, n = 34), tumor size (< 56 mm^3, n = 24; > 56 mm^3, n = 24), and liver cirrhosis (no cirrhosis, n = 20; cirrhosis, n = 28). Mean ± SD. Statistical significance determined by paired two-tailed t-test.

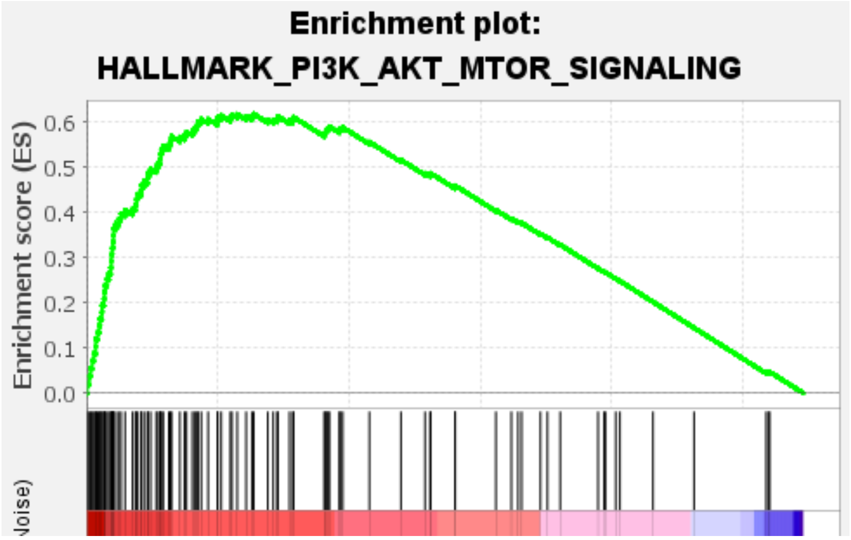

Supplementary Figure 15. HCC gene expression data from TCGA database was grouped according to RBPJ expression and subjected to GSEA analysis (n = 371). Enrichment plot showing the enriched pathways in the RBPJ^{high} expression group.

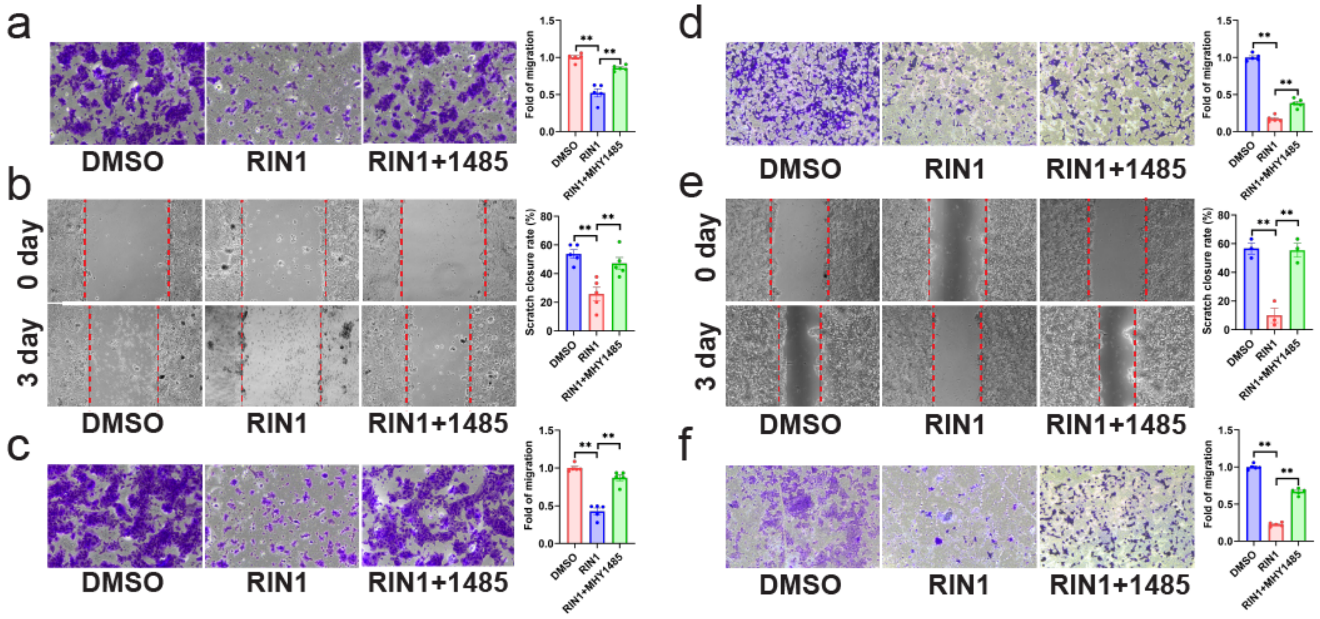

Supplementary Figure 16. MHY1485 attenuated the inhibitory effect of RIN1 on migration and invasion of HCC cells. Transwell migration (**a**, **d**), wound healing (**b**, **e**) and transwell matrigel invasion assaies (**c**, **f**) were used to analyze the migration and invasion of Huh7 (**a**-**c**) and HepG2 (**d**-**e**) cells from DMSO, RIN1 (10 μM), RIN1 with MHY1485 (10 μM)-treated groups (n = 5). Mean ± SD. Statistical significance determined by paired two-tailed t-test (a-f). *P < 0.05; **P < 0.01.

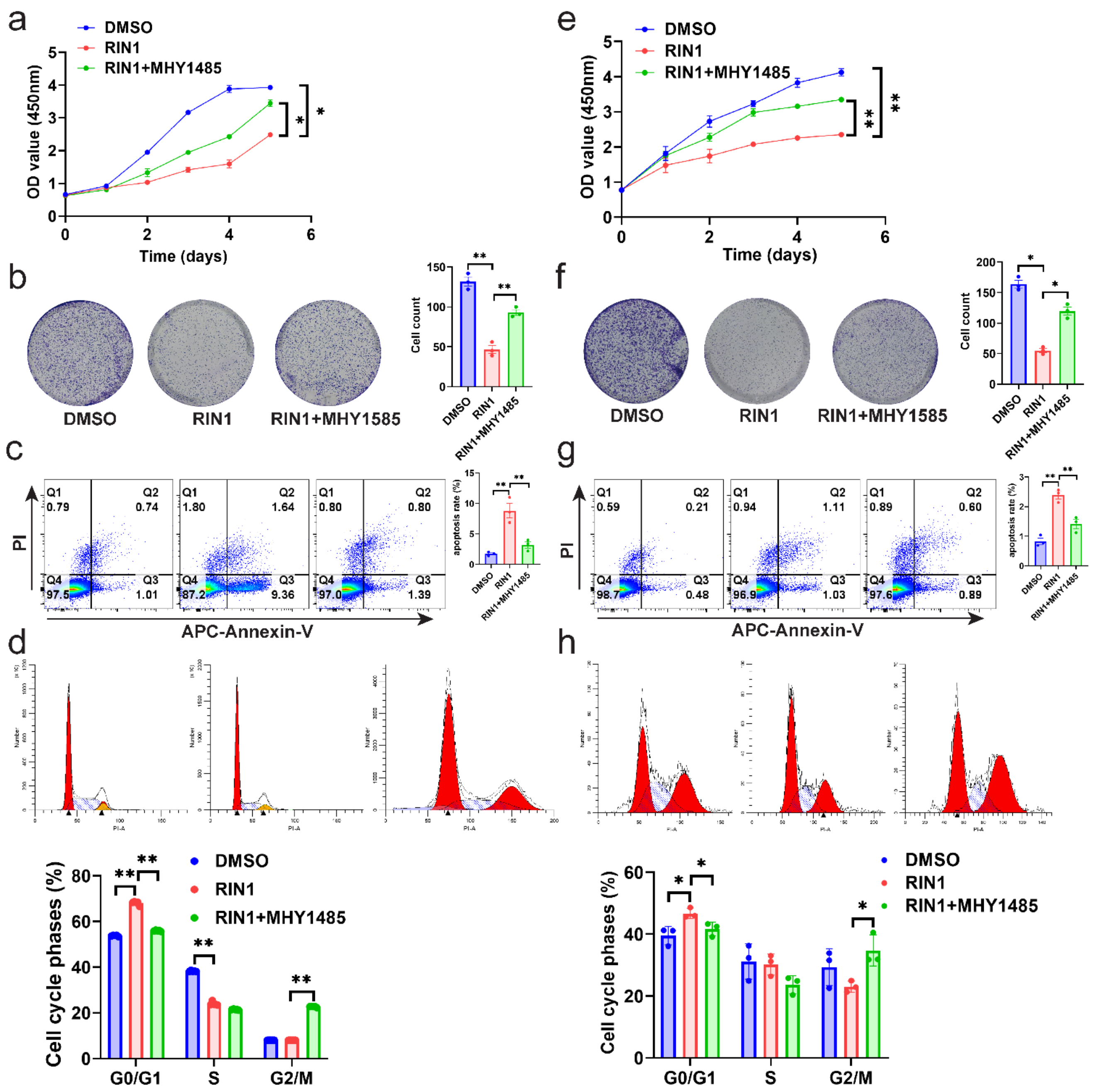

Supplementary Figure 17. MHY1485 attenuated the inhibitory effect of RIN1 on proliferation, apoptosis tolerance and cell cycle of HCC cell. a, **e** CCK8 assay was performed to examine cell viability of Huh7 (a) and HepG2 (e) cells (n = 3). **b**, **f** Colony forming assay was used to evaluate colony forming capacity of Huh7 (b) and HepG2 (f) cells (n = 3). **c**, **g** Annexin-V APC/PI showing apoptosis rate of Huh7 (c) and HepG2 (g) cells (n = 3). **d**, **h** PI/RNase were employed to detect cell cycle distribution of Huh7 (d) and HepG2 (h) cells (n = 3). Mean ± SD. Statistical significance determined by paired two-tailed t-test (a-h). *P < 0.05; **P < 0.01.

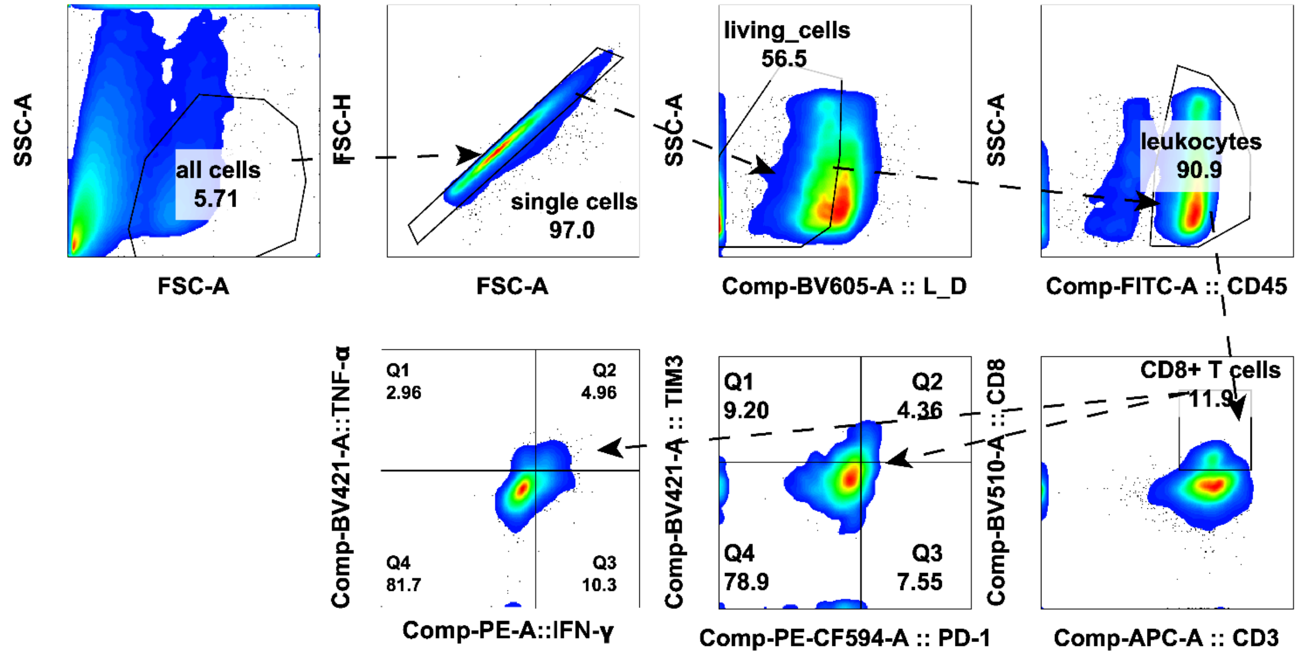

Supplementary Figure 18. The flow cytometry gating strategy to identify tumor infiltrating CD8^{+} T cells in mouse.

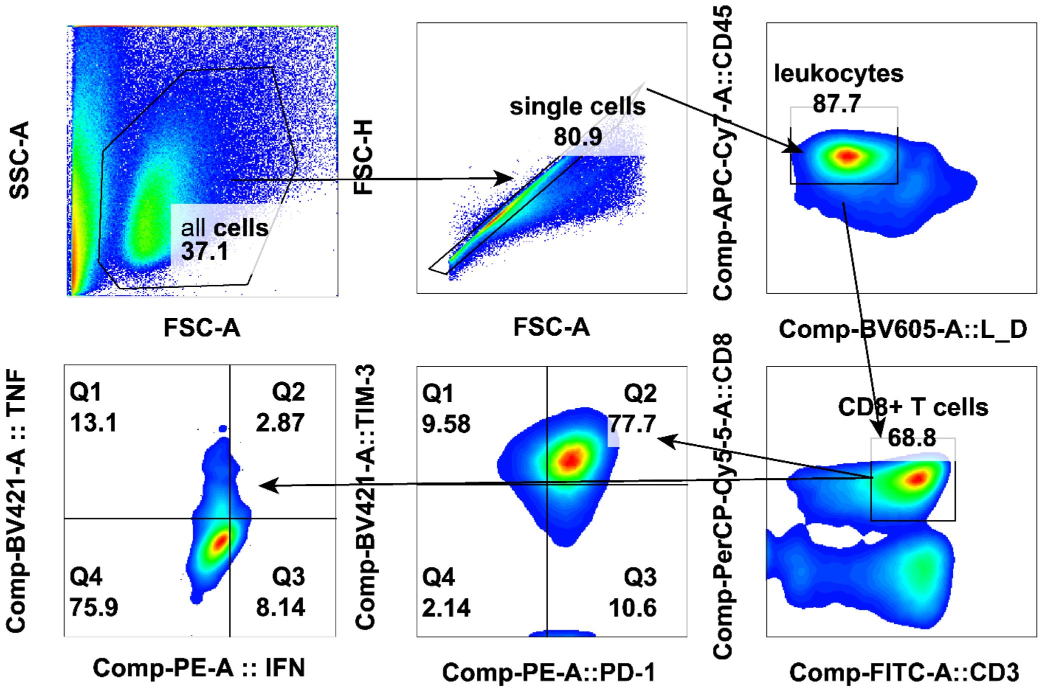

Supplementary Figure 19. The flow cytometry gating strategy to identify tumor infiltrating CD8^{+} T cells in human.

Supplementary Table

Supplementary Table 1. Clinical information of patients with HCC and the expression of PD-1 and RBPJ on their tumor-infiltrating CD8$^+$ T cells.

HCC sample	HBV infection	Pathology type	Fatty liver	T	N	MFI of PD-1 in CD8$^+$ T	MFI of RBPJ in CD8$^+$ T
HCC_244	HBV negative	Diffuse	NAFL	T1+2	N0	6.91	5.9
HCC_182	HBV negative	Diffuse	NAFL	T1+2	N0	6.96	5.93
HCC_44	HBV negative	Massive	NAFL	T3+4	N0	7.03	5.61
HCC_121	HBV negative	Nodular	AFL	T1+2	N0	7.12	5.61
HCC_24	HBV negative	Nodular	AFL	T1+2	N0	10.1	5.65
HCC_277	HBV negative	Nodular	AFL	T1+2	N0	10.1	5.99
HCC_316	HBV negative	Nodular	AFL	T1+2	N0	10.1	6.18
HCC_33	HBV negative	Nodular	AFL	T1+2	N0	15.4	6.19
HCC_261	HBV positive	Nodular	NAFL	T1+2	N0	15.5	6.24
HCC_88	HBV positive	Nodular	AFL+NAFL	T1+2	N0	15.8	6.26
HCC_59	HBV positive	Nodular	AFL	T1+2	N0	7.83	6.27
HCC_258	HBV positive	Nodular	NAFL	T1+2	N0	7.87	6.31
HCC_266	HBV positive	Diffuse	NAFL	T1+2	N0	7.9	6.32
HCC_286	HBV positive	Nodular	NAFL	T1+2	N0	8.14	6.32
HCC_94	HBV positive	Nodular	NAFL	T1+2	N0	8.98	6.37
HCC_199	HBV positive	Diffuse	NAFL	T1+2	N0	9	6.4
HCC_290	HBV positive	Nodular	NAFL	T1+2	N0	9.31	6.44
HCC_265	HBV positive	Nodular	NAFL	T1+2	N0	9.34	6.44
HCC_49	HBV positive	Diffuse	AFL+NAFL	T1+2	N0	9.59	7.21
HCC_233	HBV positive	Nodular	AFL	T1+2	N0	9.59	7.39
HCC_73	HBV positive	Diffuse	AFL+NAFL	T1+2	N0	9.99	7.45
HCC_77	HBV positive	Diffuse	AFL+NAFL	T1+2	N0	10.2	7.65
HCC_111	HBV positive	Diffuse	AFL	T1+2	N0	10.3	7.79
HCC_265	HBV positive	Diffuse	NAFL	T1+2	N0	10.6	7.06
HCC_212	HBV positive	Nodular	NAFL	T1+2	N0	10.9	7.17
HCC_179	HBV positive	Nodular	AFL	T1+2	N0	11	7.31
HCC_21	HBV positive	Nodular	NAFL	T1+2	N1	11	7.34
HCC_288	HBV positive	Diffuse	AFL	T1+2	N0	11.1	7.4
HCC_112	HBV positive	Nodular	AFL	T1+2	N0	11.5	7.48
HCC_127	HBV positive	Diffuse	AFL	T1+2	N0	11.8	7.55
HCC_212	HBV positive	Nodular	AFL	T1+2	N0	9.29	7.58
HCC_158	HBV positive	Diffuse	AFL	T1+2	N0	9.35	7.71
HCC_162	HBV positive	Nodular	NAFL	T1+2	N0	9.78	8.02
HCC_160	HBV positive	Diffuse	NAFL	T1+2	N0	9.96	8.29
HCC_55	HBV positive	Diffuse	NAFL	T1+2	N0	10.1	8.54
HCC_53	HBV positive	Nodular	NAFL	T1+2	N0	6.18	8.86

HCC_88	HBV positive	Diffuse	NAFL	T1+2	N0	6.22	13.3
HCC_156	HBV positive	Nodular	NAFL	T1+2	N0	6.34	13.5
HCC_96	HBV positive	Nodular	NAFL	T1+2	N0	14	13.6
HCC_78	HBV positive	Massive	none	T3+4	N0	14.5	13.8
HCC_98	HBV positive	Nodular	AFL	T1+2	N0	14.6	13.9
HCC_316	HBV positive	Massive	none	T3+4	N0	14.7	10.8
HCC_283	HBV positive	Diffuse	none	T1+2	N0	15.2	11.1
HCC_298	HBV positive	Nodular	AFL	T1+2	N0	4.29	11.8
HCC_287	HBV positive	Nodular	none	T1+2	N0	8.77	12.5
HCC_252	HBV positive	Nodular	AFL	T1+2	N1	8.85	12.5
HCC_147	HBV positive	Nodular	none	T1+2	N0	8.86	13
HCC_102	HBV positive	Diffuse	AFL	T1+2	N0	17.3	14.1
HCC_172	HBV positive	Diffuse	none	T1+2	N0	19.1	16.8
HCC_213	HBV positive	Diffuse	AFL	T1+2	N0	21.8	21.9
HCC_89	HBV positive	Nodular	AFL+NAFL	T1+2	N0	7	12.2
HCC_167	HBV positive	Diffuse	AFL+NAFL	T1+2	N0	7.06	12.6
HCC_143	HBV positive	Diffuse	AFL	T1+2	N0	7.12	12.8
HCC_277	HBV positive	Diffuse	AFL+NAFL	T1+2	N0	5.67	6.35
HCC_250	HBV positive	Diffuse	AFL+NAFL	T1+2	N0	5.89	6.37
HCC_181	HBV positive	Massive	NAFL	T3+4	N1	6.51	6.42
HCC_24	HBV positive	Nodular	NAFL	T1+2	N1	6.8	6.47
HCC_112	HBV positive	Nodular	NAFL	T1+2	N0	6.93	6.48
HCC_302	HBV positive	Nodular	NAFL	T1+2	N0	6.63	5.51
HCC_272	HBV positive	Diffuse	AFL	T1+2	N0	6.7	5.94
HCC_291	HBV positive	Diffuse	none	T1+2	N0	6.76	6.03
HCC_312	HBV positive	Massive	none	T3+4	N0	6.79	5.94
HCC_220	HBV positive	Diffuse	AFL	T1+2	N0	6.87	5.97

Abbreviations: AFLD: alcoholic fatty liver disease, HBV: hepatitis B virus, HCC: hepatocellular carcinoma, MFI: mean fluorescence intensity, NAFLD: non-alcoholic fatty liver disease.

Supplemental Materials

Reagent/Resource	Reference or Source	Identifier or Catalog Number
**Experimental Models**		
Hepa1-6 cells (M. musculus)	Shanghai Institute of Biochemistry and Cell Biology	Cat# SCSP-512
HepG2 cells (H. sapiens)	Shanghai Institute of Biochemistry and Cell Biology	Cat# SCSP-510
Huh7 cells (H. sapiens)	Shanghai Institute of Biochemistry and Cell Biology	Cat# SCSP-526
C57BL/6 mice	Vital River Laboratory Animal Technology Co. Ltd	N/A
Nude mice	Vital River Laboratory Animal Technology Co. Ltd	N/A
**Recombinant DNA**		
pCDNA3.1(+)	Thermo Fisher Scientific	Cat# V79020
pLKO.1	Addgene	Cat# 8453
pLenti	Addgene	Cat# 39481
psPAX2	Addgene	Cat# 12260
pMD2.G	Addgene	Cat# 12259
**Antibodies**		
PerCP/Cyanine5.5 anti-human Ki-67 Antibody	Biolegend	Cat# 350519
PE anti-human CD274 (B7-H1, PD-L1) Antibody	Biolegend	Cat# 329705
PE anti-human CD273 (B7-DC, PD-L2) Antibody	Biolegend	Cat# 329605
PE anti-human HLA-A,B,C Antibody	Biolegend	Cat# 311405
FITC anti-mouse CD45 Antibody	Biolegend	Cat# 157213
FITC anti-mouse CD3 Antibody	Biolegend	Cat# 100203
APC anti-mouse CD3 Antibody	Biolegend	Cat# 100235
APC anti-mouse CD8 Antibody	Biolegend	Cat# 100711
PerCP/Cyanine5.5 anti-mouse CD4 Antibody	Biolegend	Cat# 100431
Brilliant Violet 510™ anti-mouse CD8a Antibody	Biolegend	Cat# 100751

Brilliant Violet 650™ anti-mouse F4/80 Antibody	Biolegend	Cat# 123149
APC anti-mouse CD206 (MMR) Antibody	Biolegend	Cat# 141707
PE anti-mouse NK-1.1 Antibody	Biolegend	Cat# 156503
PE/Dazzle™ 594 anti-mouse CD279 (PD-1) Antibody	Biolegend	Cat# 135227
Brilliant Violet 421™ anti-mouse CD366 (Tim-3) Antibody	Biolegend	Cat# 134019
PE anti-mouse IFN-γ Antibody	Biolegend	Cat# 505807
Brilliant Violet 421™ anti-mouse TNF-α Antibody	Biolegend	Cat# 506327
APC/Cyanine7 anti-human CD45 Antibody	Biolegend	Cat# 368515
FITC anti-human CD3 Antibody	Biolegend	Cat# 317305
FITC anti-human CD8	Biolegend	Cat# 980908
PerCP/Cyanine5.5 anti-human CD8	Biolegend	Cat# 980918
PE anti-human CD279 (PD-1) Antibody	Biolegend	Cat# 329905
Brilliant Violet 421™ anti-human CD366 (Tim-3) Antibody	Biolegend	Cat# 345007
PE anti-human IFN-γ Antibody	Biolegend	Cat# 502508
Brilliant Violet 421™ anti-human TNF-α Antibody	Biolegend	Cat# 502931
PE anti-mouse FOXP3 Antibody	Biolegend	Cat# 126403
FITC anti-mouse CD25 Antibody	Biolegend	Cat# 101907
Brilliant Violet 605™ anti-mouse CD45 Antibody	Biolegend	Cat# 103139
Anti-RBPJK antibody	Abcam	Cat# ab180588
RBPSUH (D10A4) XP® Rabbit mAb	Cell Signaling Technology	Cat# 5313T
Anti-PD1 antibody	Abcam	Cat# ab237728
Anti-CD8 alpha antibody	Abcam	Cat# ab237709
Anti-mTOR antibody	Abcam	Cat# ab134903
Anti-mTOR (phospho S2448) antibody	Abcam	Cat# ab177734
Anti-S6K1 antibody	Abcam	Cat# ab32529
Anti-S6K1 (phospho T389 + T412) antibody	Abcam	Cat# ab60948
Anti-eIF4EBP1 antibody	Abcam	Cat# ab32024

Anti-eIF4EBP1 (phospho T37) antibody	Abcam	Cat# ab75767
Anti-AKT1 antibody	Abcam	Cat# ab108202
Anti-AKT1 (phospho S473) antibody	Abcam	Cat# ab81283
Anti-RPS6 (phospho S240 + S244) antibody	Abcam	Cat# ab225676
Anti-RPS6 antibody	Abcam	Cat# ab225676
Anti-RPS16 antibody	Abcam	Cat# ab177951
Anti-Hexokinase II antibody	Abcam	Cat# ab209847
Anti-Glucose Transporter GLUT1 antibody	Abcam	Cat# ab115730
Anti-PKM2 antibody	Abcam	Cat# ab85555
Anti-c-Myc antibody	Abcam	Cat# ab32072
Anti-PD-L1 antibody	Abcam	Cat# ab205921
Anti-PD-L2 antibody	Abcam	Cat# ab288298
Anti-HLA Class 1 ABC antibody	Abcam	Cat# ab225636
Anti-Tryptophan Hydroxylase/TPH antibody	Abcam	Cat# ab52954
Anti-Indoleamine 2, 3-dioxygenase antibody	Abcam	Cat# ab211017
Anti-GAPDH antibody	Abcam	Cat# ab8254
Anti-beta Actin antibody	Abcam	Cat# ab8226
Anti-F4/80 antibody	Abcam	Cat# 111101
Goat Anti-Mouse IgG H&L (FITC)	Abcam	Cat# ab6785
Goat Anti-Rabbit IgG H&L (Cy5 ®) preadsorbed	Abcam	Cat# ab6564
89Y anti-mouse CD45	Biolegend	Cat# 103101
115In anti-mouse CD3e	Biolegend	Cat# 152302
142Nd anti-mouse CD127	Biolegend	Cat# 135002
143Nd anti-mouse TIM-3	Biolegend	Cat# 165402
144Nd anti-mouse CD44	Biolegend	Cat# 156002
145Nd anti-mouse CTLA-4	Biolegend	Cat# 106202
150Nd anti-mouse CD25	Biolegend	Cat# 101902
151Eu anti-mouse GZMB	Biolegend	Cat# 396402
152Sm anti-mouse CD19	Biolegend	Cat# 115502
153Eu anti-mouse CD62L	Biolegend	Cat# 104402
157Gd anti-mouse CD335	Biolegend	Cat# 137625
160Gd anti-mouse CD45R	Biolegend	Cat# 103201
162Dy anti-mouse FOXP3	Biolegend	Cat# 320001
167Er anti-mouse F4/80	Biolegend	Cat# 123143

171Yb anti-mouse CD69	Biolegend	Cat# 104533
172Yb anti-mouse PD-1	Biolegend	Cat# 135202
173Yb anti-mouse ICOS	Biolegend	Cat# 313502
158Gd anti-mouse IgM	Biolegend	Cat# 406527
197Au anti-mouse CD4	Biolegend	Cat# 100505
198Pt anti-mouse CD8a	Biolegend	Cat# 100755
209Bi anti-mouse CD11b	Biolegend	Cat# 101201
165Ho anti-mouse TCRgd	Biolegend	Cat# 107515
164Dy anti-mouse TCF-7	Biolegend	Cat# 655202
156Gd anti-mouse TOX	Biolegend	Cat# 682601
141Pr anti-mouse CD163	Biolegend	Cat# 156702
155Gd anti-mouse CD80	Biolegend	Cat# 104735
**Oligonucleotides and other sequence-based reagents**		
Has-RBPJ-F	5'-AACAAATGGAACGCGATGGTT-3'	122
Has-RBPJ-R	5'-GGCTGTGCAATAGTTCTTTCCTT-3'	
Mmu-*Rbpj*-F	5'-AGTTGCACAGAAGTCTTACGG-3'	156
Mmu-*Rbpj*-R	5'-CCTATTCCAATAAACGCACAGGG-3'	
Mmu-*Rps16*-F	5'-CAGGTCTTCGGACGCAAGAAA-3'	102
Mmu-*Rps16*-R	5'-CGGCTCGATCATCTCCAGG-3'	
Has-PDCD1-F	5'-CCAGGATGGTTCTTAGACTCCC-3'	137
Has-PDCD1-R	5'-TTTAGCACGAAGCTCTCCGAT-3'	
Has-HAVCR2-F	5'-TTGGACATCCAGATACTGGCT-3'	86
Has-HAVCR2-R	5'-CACTGTCTGCTAGAGTCACATTC-3'	
Has-CD45-F	5'-ACCACAAGTTTACTAACGCAAGT-3'	126
Has-CD45-R	5'-TTTGAGGGGGATTCCAGGTAAT-3'	
Has-CD3G-F	5'-TGGCCCAGTCAATCAAAGGAA-3'	76
Has-CD3G-R	5'-CAAGTCAGAAGTACCGAACCATC-3'	
Has-CD4-F	5'-TGCCTCAGTATGCTGGCTCT-3'	193
Has-CD4-R	5'-GAGACCTTTGCCTCCTTGTTC-3'	
Has-CD8A-F	5'-ATGGCCTTACCAGTGACCG-3'	104
Has-CD8A-R	5'-AGGTTCCAGGTCCGATCCAG-3'	
Has-CD206-F	5'-TCCGGGTGCTGTTCTCCTA-3'	211
Has-CD206-R	5'-CCAGTCTGTTTTTGATGGCACT-3'	
Has-CD335-F	5'-TGGACCCGAAGTGATCTCG-3'	84

Has-CD335-R	5'-TCCTTGAGCAGTAAGAACATGC-3'	
Has-HK2-F	5'-GAGCCACCACTCACCCTACT-3'	249
Has-HK2-R	5'-CCAGGCATTCGGCAATGTG-3'	
Has-GLUT1-F	5'-GGCCAAGAGTGTGCTAAAGAA-3'	201
Has-GLUT1-R	5'-GGCCAAGAGTGTGCTAAAGAA-3'	
Has-PKM2-F	5'-ATGTCGAAGCCCCATAGTGAA-3'	118
Has-PKM2-R	5'-TGGGTGGTGAATCAATGTCCA-3'	
Has-HIF1A-F	5'-GAACGTCGAAAAGAAAAGTCTCG-3'	124
Has-HIF1A-R	5'-CCTTATCAAGATGCGAACTCACA-3'	
Has-MYC-F	5'-GGCTCCTGGCAAAAGGTCA-3'	119
Has-MYC-R	5'-GGCTCCTGGCAAAAGGTCA-3'	
Has-TPH1-F	5'-ACGTCGAAAGTATTTTGCGGA-3'	102
Has-TPH1-R	5'-ACGGTTCCCCAGGTCTTAATC-3'	
Has-IDO1-F	5'-GCCAGCTTCGAGAAAGAGTTG-3'	96
Has-IDO1-R	5'-ATCCCAGAACTAGACGTGCAA-3'	
Has-IDO2-F	5'-CCACAGACCGAATGTGAAGAC-3'	137
Has-IDO2-R	5'-TGTTGGCAATTTCCATCCAAGG-3'	
Has-TDO2-F	5'-AAGGTTGTTTCTCGGATGCAC-3'	81
Has-TDO2-R	5'-TGTCATCGTCTCCAGAATGGAA-3'	
Has-GAPDH-F	5'-GGAGCGAGATCCCTCCAAAAT-3'	197
Has-GAPDH-R	5'-GGCTGTTGTCATACTTCTCATGG-3'	
Has-beta Actin-F	5'-CATGTACGTTGCTATCCAGGC-3'	250
Has-beta Actin-R	5'-CTCCTTAATGTCACGCACGAT-3'	
Mmu-*Gapdh*-F	5'-AGGTCGGTGTGAACGGATTTG-3'	95
Mmu-*Gapdh*-R	5'-GGGGTCGTTGATGGCAACA-3'	
Mmu-*beta Actin*-F	5'-GGCTGTATTCCCCTCCATCG-3'	154
Mmu-*beta Actin*-R	5'-CCAGTTGGTAACAATGCCATGT-3'	
Rbpj-KD sense	5'-CACCGCCATTTCCACAAGCCAATAGCGAACTATTGGCTTGTGGAAATGGC -3'	N/A
Rbpj-KD anti-sense	3'-CGGTAAAGGTGTTCGGTTATCGCTTGATAACCGAACACCTTTACCGAAAA -5'	

**Chemicals, Enzymes and other reagents**		
RBPJ Inhibitor-1 (RIN1)	MedChemExpress	Cat# HY-137471
Bevacizumab	MedChemExpress	Cat# HY-P9906
Clodronate liposomes	Yeasen	Cat# 40338ES05
Diethylnitrosamine (DEN)	Merck	Cat# 442687
Carbon tetrachloride (CCl_4)	Macklin	Cat# 56-23-5
Olive oil	MedChemExpress	Cat# HY-Y1888
Fetal bovine serum (FBS)	Thermo Fisher Scientific	Cat# 10082147
L-Kynurenine	MedChemExpress	Cat# HY-104026
IDO1-IN-18	MedChemExpress	Cat# HY-144651
Rodatristat	MedChemExpress	Cat# HY-120083
MHY1485	MedChemExpress	Cat# HY-B0795
CD8 (TIL) MicroBeads, human	Miltenyi	Cat# 130-045-201
CD8 (TIL) MicroBeads, mouse	Miltenyi	Cat# 130-116-478
*InVivo*Plus anti-mouse PD-1 (CD279)	Bioxcell	Cat# BP0146
*InVivo*MAb anti-mouse PD-L1 (B7-H1)	Bioxcell	Cat# BE0101
Dulbecco's Modified Eagle Medium (DMEM)	Thermo Fisher Scientific	Cat# 11965092
Advanced RPMI 1640 Medium	Thermo Fisher Scientific	Cat# 12633012
Streptomycin, penicillin	Thermo Fisher Scientific	Cat# 15070063
PI/RNase	BD biosciences	Cat# 550825
PI	BD biosciences	Cat# 550825
Lipo3000	Thermo Fisher Scientific	Cat# L3000001
Leukocyte Activation Cocktail, with BD GolgiPlug™	BD biosciences	Cat# 550583
Fixable Viability Stain 575V (FVS575V)	BD biosciences	Cat# 565694
Fixable Viability Stain 450V (FVS450V)	BD biosciences	Cat# 562247
Annexin V-APC	BD biosciences	Cat# 561012
Improved Citrate Antigen Retrieval	Beyotime	Cat# P0083

Solution		
Percoll	Solarbio	Cat# P8370
Polybrene	Yeasen	Cat# 40804ES76
Cell Staining Buffer	Biolegend	Cat# 420201
RIPA Lysis Buffer	Beyotime	Cat# P0013B
QuickBlock™ Blocking Buffer for Western Blot	Beyotime	Cat# P0252
SuperSignal West Dura	Thermo Fisher Scientific	Cat# 34076
FastPure Plant Total RNA Isolation Kit	Vazyme	Cat# RC401
HiScript II Q Select RT SuperMix for qPCR	Vazyme	Cat# R232-01
ChamQ SYBR qPCR Master Mix	Vazyme	Cat# Q321-02
Kynurenine ELISA kit	Abcam	Cat# ab287800
ATP Assay Kit	Beyotime	Cat# S0026
VAHTS® mRNA-seq V3 Library Prep Kit for Illumina	Vazyme	Cat# NR611-01
Cell Counting Kit 8	Abcam	Cat# ab228554
BCA Protein Quantification Kit	Vazyme	Cat# E112-01
Deoxyribonuclease-1 (DNase I)	Thermo Fisher Scientific	Cat# 18047019
Collagenase IV	Corning	Cat# 354245
Ethylenediaminetetraacetic acid (EDTA)	Thermo Fisher Scientific	Cat# 17892
Phosphate Buffered Saline (PBS)	Solarbio	Cat# P1020
Cytofix/Cytoperm and PermWash Buffer	BD Biosciences	Cat# 554723
Foxp3 staining kit	Biolegend	Cat# 421403
16% paraformaldehyde aqueous solution	Electron Microscopy Sciences/ LucernaChem	Cat#15710
2-methylbutane	Sigma Aldrich	Cat# M32631
Triton X-100	Solarbio	Cat# T8200
Dimethyl sulfoxide (DMSO)	Fischer Bioreagents	Cat# BP231-1
Matrigel	Corning	Cat# 354277
Trizol	Thermo Fisher Scientific	Cat# 15596026

Hanks' Balanced Salt Solution (HBSS)	ThermoFisher Scientific	Cat# 14175095
Crystal violet	Solarbio	Cat# C8470
MS column	Miltenyi Biotec	Cat# 130-122-727
Protease inhibitor mixture	Solarbio	Cat# P6730
Bovine serum albumin (BSA)	Solarbio	Cat# A8010
180 kDa Prestained Protein Marker	Vazyme	Cat# MP102-01
GoldBand Plus 3-color High Range Protein Marker(25-300 KDa)	Yeasen	Cat# 20347ES72
Dead Cell Removal Kit	Miltenyi	Cat# 130-090-101
Hyperactive Universal CUT&Tag Assay Kit for Illumina	Vazyme	Cat# TD903-01
EQ Four Element Calibration	Fluidigm	Cat# 201078
Antibody Stabilizer	Candor Bioscience	Cat# 131050
Ethanol	Sigma	Cat# 493511
Bambanker	LubioScience GmbH	Cat# 523303 (BB02)
Cell-ID™ Intercalator-Ir	Fluidigm	Cat# 201192B
Cell-ID Cisplatin	Fluidigm	Cat# 201064
Cryo Embedding Medium	Medite	Cat# 41-3011-00
Human TruStain FcX™ (Fc Receptor Blocking Solution)	Biolegend	Cat# 422302
TruStain FcX™ (anti-mouse CD16/32) Antibody	Biolegend	Cat# 101319
Indium (115-In)	Trace Sciences International	N/A
Iridium (191-Ir, 193-Ir)	Fluidigm	Cat# 201192A
Isoflurane	Minrad	N/A
Maxpar X8 Multimetal Labeling Kit	Fluidigm	Cat# 201300
Maxpar Fix and Perm Buffer	Fluidigm	Cat# 201067
2-NBDG Glucose Uptake Assay Kit	Abcam	Cat# ab287845
Pyruvate Assay Kit	Abcam	Cat# ab65342
**Software**		

GraphPad Prism (version 8.0)	GraphPad Software Inc	https://www.graphpad.com/ scientificsoftware/prism/
FlowJo (version7.6.1)	FlowJo, LLC	https://www.flowjo.com/ solutions/flowjo
Cytobank	Beckman, LLC	https://premium.cytobank.cn/cytobank/experiments
SPSS V.19	IBM, LLC	https://www.ibm.com/cn-zh/spss
R version 3.5	R Development Core Team	https://www.r-project.org/
R Studio	RStudio Team, 2015	https://www.rstudio.com/
ImageJ	National Institute of Health (NIH)	https://imagej.nih.gov/ij/
