# Supplementary material for: Targeted inhibition of RBPJ transcription complex alleviates the exhaustion of CD8+ T cells in hepatocellular carcinoma
Source: Commun Biol. 2023 Jan 30;6:123. doi: 10.1038/s42003-023-04521-x (PMC9887061; doi:10.1038/s42003-023-04521-x)
Supplement: Supplementary file 2 — Description of Additional Supplementary Files [file 42003_2023_4521_MOESM2_ESM.pdf]

Description of Additional Supplementary Files

File name: Supplementary Data 1
Description: All source data underlying the graphs and charts.

File name: Supplementary Data 2
Description: All blots accompanied by size markers in every figure panel.
