# Supplementary figures and images for: Targeted inhibition of RBPJ transcription complex alleviates the exhaustion of CD8+ T cells in hepatocellular carcinoma
Source: Commun Biol. 2023 Jan 30;6:123. doi: 10.1038/s42003-023-04521-x (PMC9887061; doi:10.1038/s42003-023-04521-x)

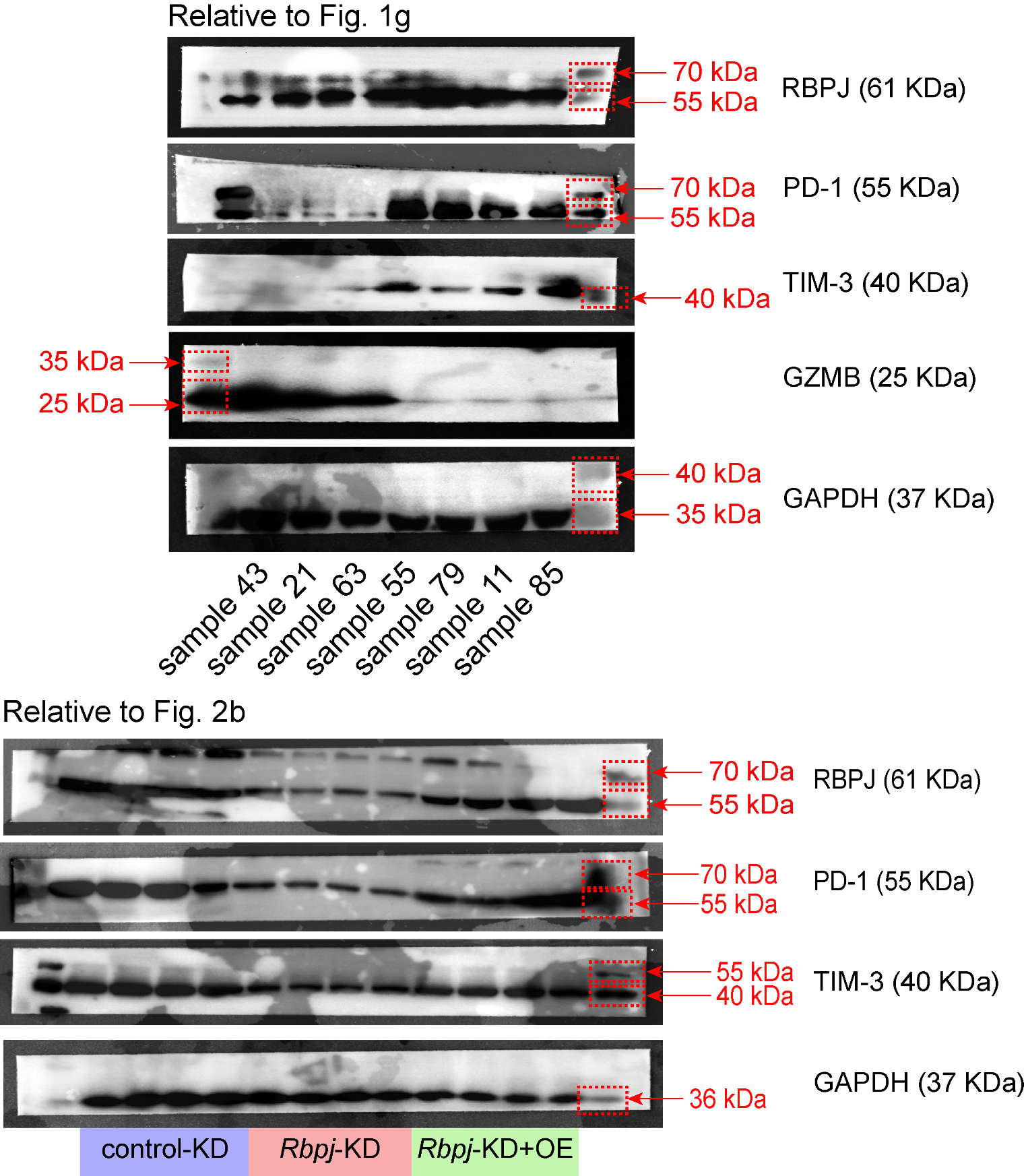


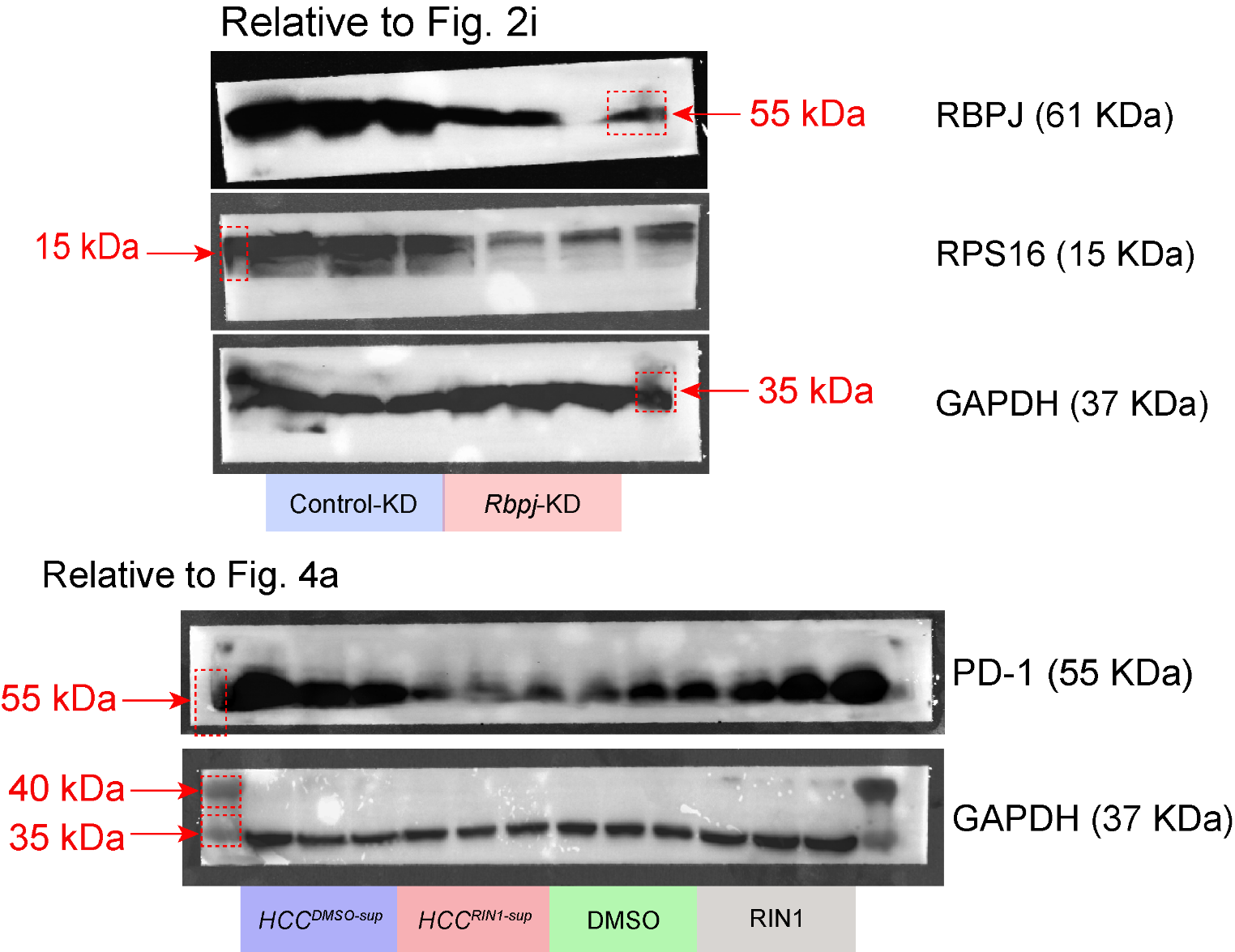


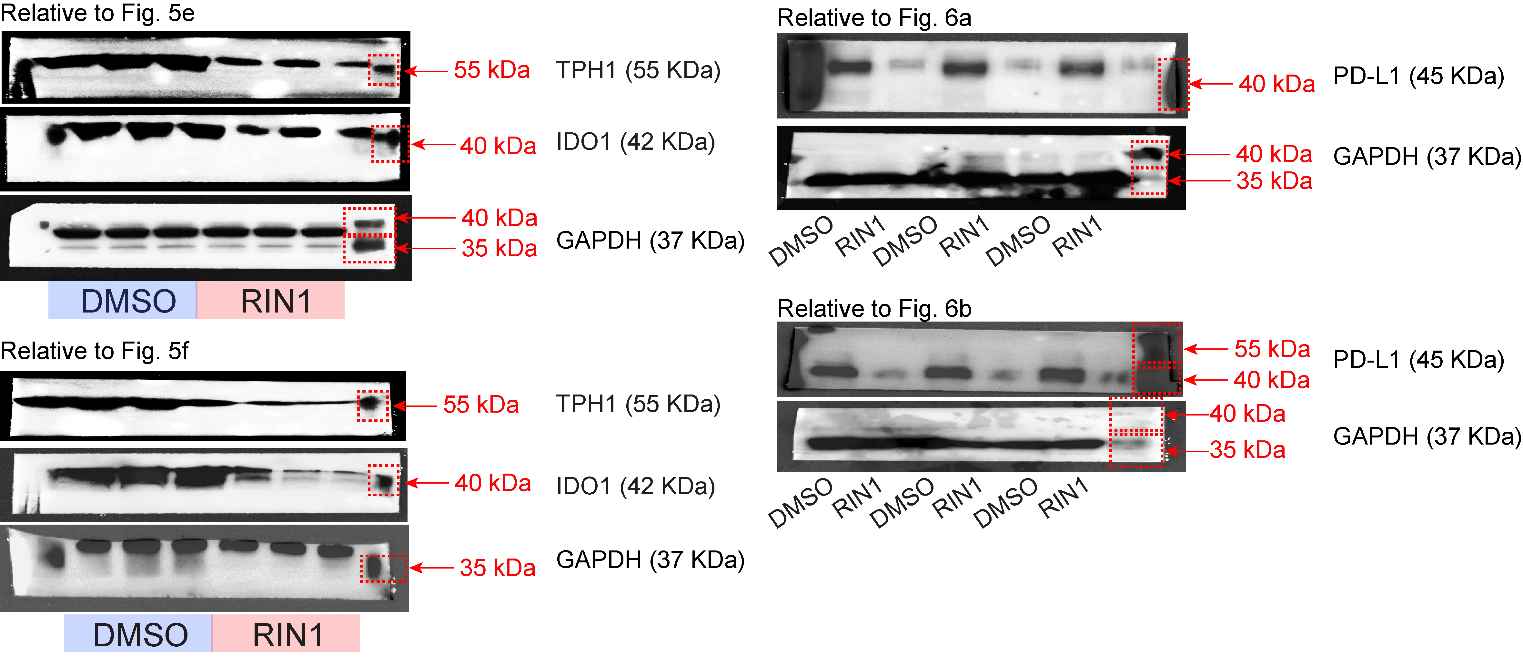


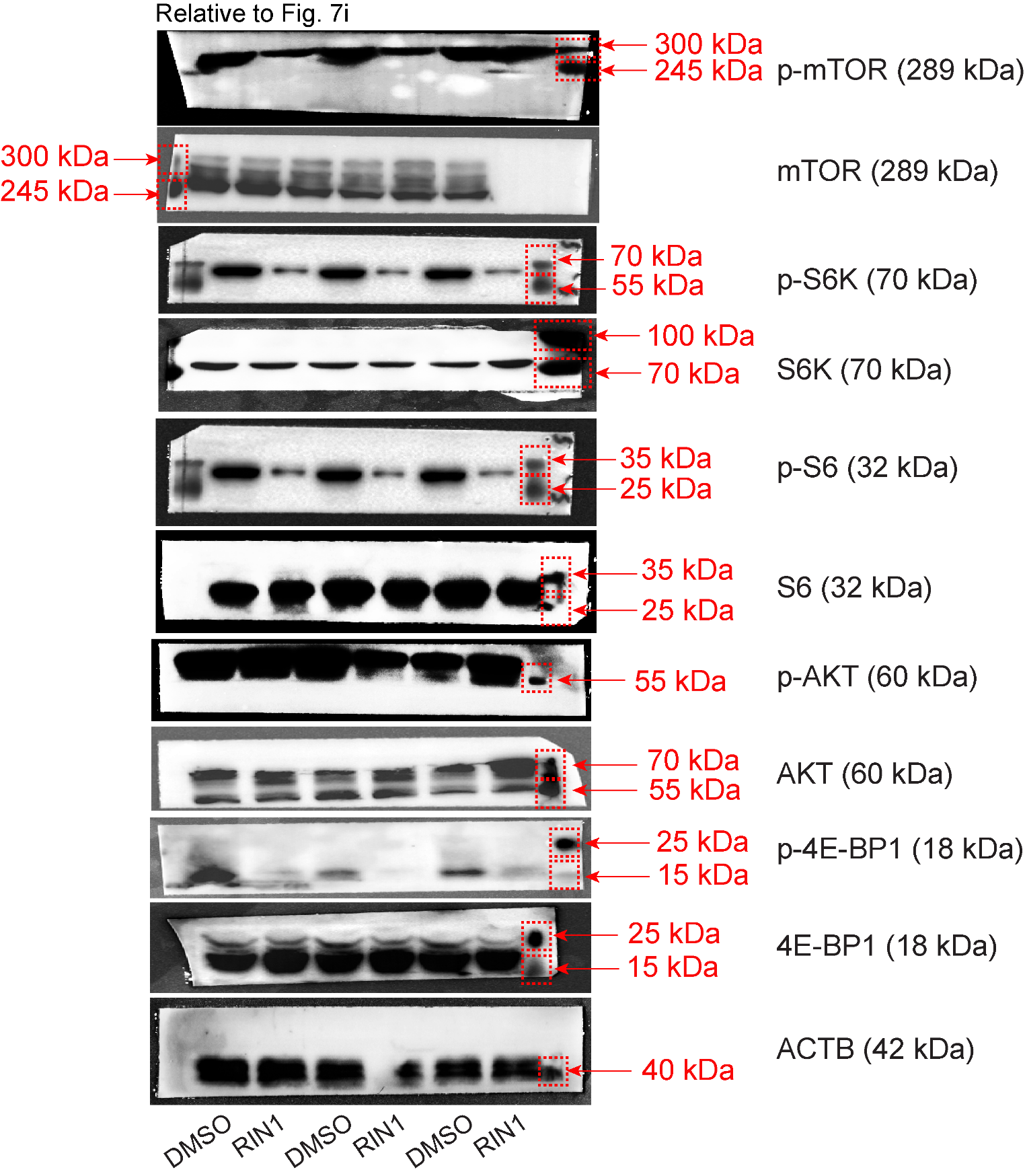


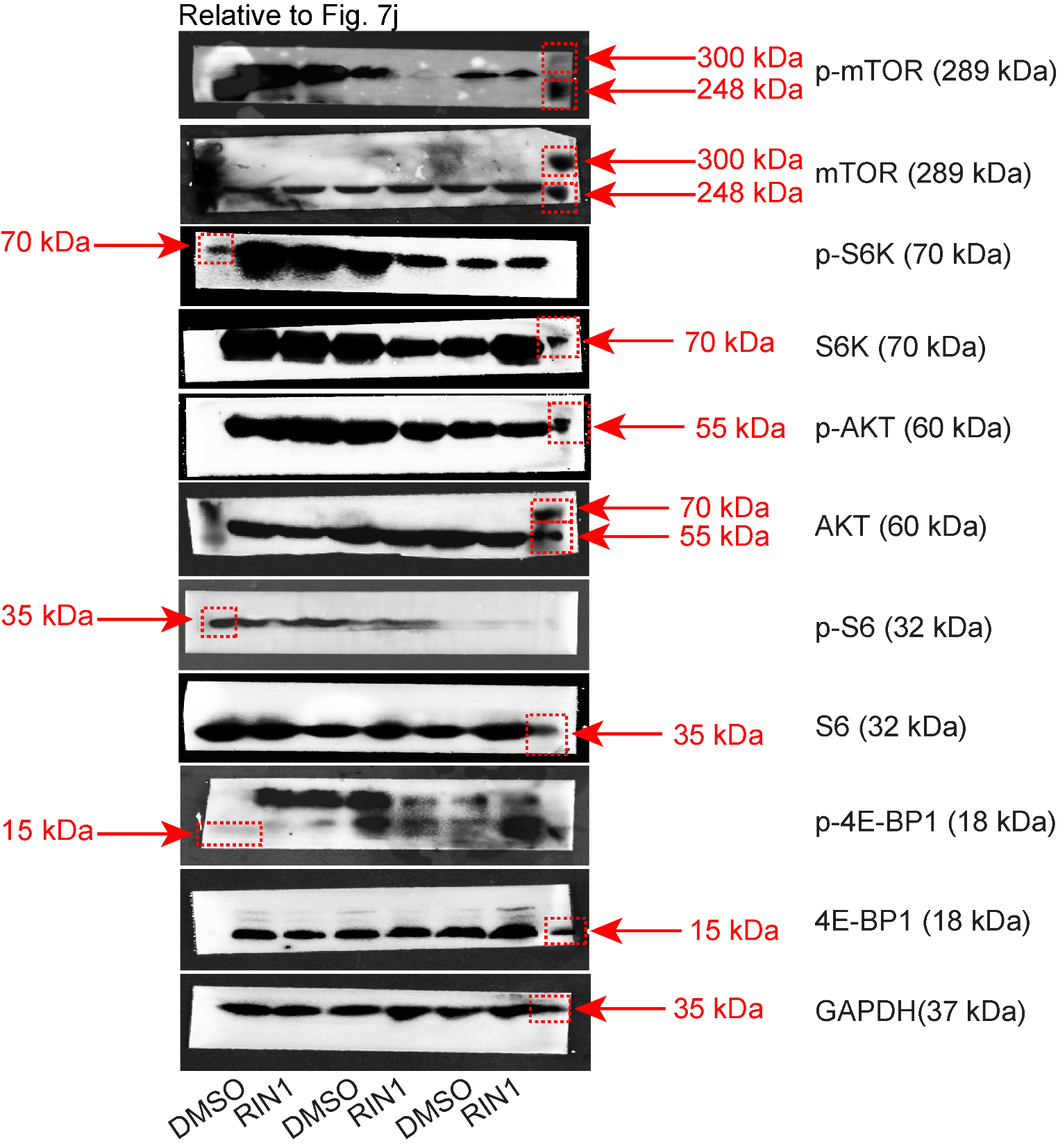


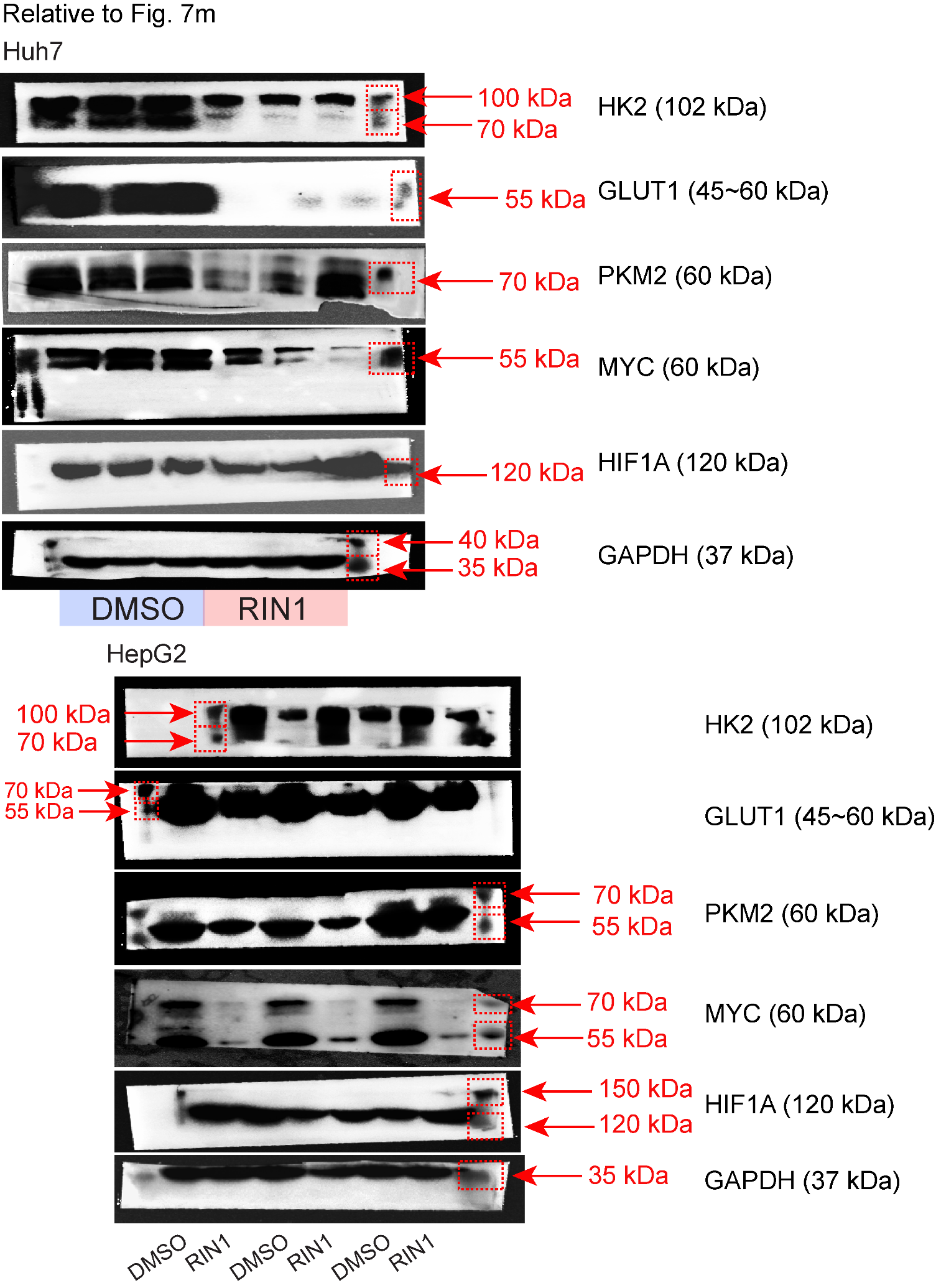

Supplement: Supplementary file 4 — Supplementary Data 2 [file 42003_2023_4521_MOESM4_ESM.docx]
