# Supplementary material for: Targeted inhibition of RBPJ transcription complex alleviates the exhaustion of CD8+ T cells in hepatocellular carcinoma
Source: Commun Biol. 2023 Jan 30;6:123. doi: 10.1038/s42003-023-04521-x (PMC9887061; doi:10.1038/s42003-023-04521-x)
Supplement: Supplementary file 5 — Reporting Summary [file 42003_2023_4521_MOESM5_ESM.pdf]

nature portfolio

Corresponding author(s): Nanhong Tang
Last updated by author(s): 2022-12-23

Reporting Summary

Nature Portfolio wishes to improve the reproducibility of the work that we publish. This form provides structure for consistency and transparency in reporting. For further information on Nature Portfolio policies, see our Editorial Policies and the Editorial Policy Checklist.

Statistics

For all statistical analyses, confirm that the following items are present in the figure legend, table legend, main text, or Methods section.

n/a	Confirmed	
☐	☑	The exact sample size (*n*) for each experimental group/condition, given as a discrete number and unit of measurement
☐	☑	A statement on whether measurements were taken from distinct samples or whether the same sample was measured repeatedly
☐	☑	The statistical test(s) used AND whether they are one- or two-sided<br>*Only common tests should be described solely by name; describe more complex techniques in the Methods section.*
☐	☑	A description of all covariates tested
☐	☑	A description of any assumptions or corrections, such as tests of normality and adjustment for multiple comparisons
☐	☑	A full description of the statistical parameters including central tendency (e.g. means) or other basic estimates (e.g. regression coefficient) AND variation (e.g. standard deviation) or associated estimates of uncertainty (e.g. confidence intervals)
☐	☑	For null hypothesis testing, the test statistic (e.g. *F*, *t*, *r*) with confidence intervals, effect sizes, degrees of freedom and *P* value noted<br>*Give P values as exact values whenever suitable.*
☐	☑	For Bayesian analysis, information on the choice of priors and Markov chain Monte Carlo settings
☐	☑	For hierarchical and complex designs, identification of the appropriate level for tests and full reporting of outcomes
☐	☑	Estimates of effect sizes (e.g. Cohen's *d*, Pearson's *r*), indicating how they were calculated

Our web collection on statistics for biologists contains articles on many of the points above.

Software and code

Policy information about availability of computer code

Data collection	NA
Data analysis	NA

For manuscripts utilizing custom algorithms or software that are central to the research but not yet described in published literature, software must be made available to editors and reviewers. We strongly encourage code deposition in a community repository (e.g. GitHub). See the Nature Portfolio guidelines for submitting code & software for further information.

Data

Policy information about availability of data

All manuscripts must include a data availability statement. This statement should provide the following information, where applicable:

- Accession codes, unique identifiers, or web links for publicly available datasets
- A description of any restrictions on data availability
- For clinical datasets or third party data, please ensure that the statement adheres to our policy

Raw data of RNA-seq of patient HCC infiltrating CD8+ T cells treated with Huh7RIN1-sup (PRJNA881457; https://www.ncbi.nlm.nih.gov/sra/?term=PRJNA881457), transcriptome and CUT&Tag sequencing of infiltrating CD8+ T cells from mouse normal liver and primary HCC (PRJNA904833, https://www.ncbi.nlm.nih.gov/bioproject/?term=PRJNA904833), single-cell RNA-seq (PRJNA880758; https://www.ncbi.nlm.nih.gov/sra/?term=PRJNA880758) are available at the SRA repository.

Human research participants

Policy information about studies involving human research participants and Sex and Gender in Research.

Reporting on sex and gender	This study did not involve sex and gender.
Population characteristics	From 2019 to 2022, all HCC and paired paracancerous tissues were obtained from Fujian Medical University Union Hospital and randomly used in this experiment (n = 28). All patients were diagnosed with HCC based on tissue specimens.
Recruitment	All patients were diagnosed with HCC based on tissue specimens.
Ethics oversight	This study protocol was reviewed and approved by the Ethics Committee of Fujian Medical University Union Hospital, approval number 2021KJCX008; and the Experimental Animal Ethics Committee of Fujian Medical University, approval number IACUC FJMU 2022-0015.

Note that full information on the approval of the study protocol must also be provided in the manuscript.

Field-specific reporting

Please select the one below that is the best fit for your research. If you are not sure, read the appropriate sections before making your selection.

☑ Life sciences ☐ Behavioural & social sciences ☐ Ecological, evolutionary & environmental sciences

For a reference copy of the document with all sections, see nature.com/documents/nr-reporting-summary-flat.pdf

Life sciences study design

All studies must disclose on these points even when the disclosure is negative.

Sample size	No less than three samples in each group for cell experiments and no less than six samples for animal experiments.
Data exclusions	No data were excluded from this analysis.
Replication	Data were obtained from three independent experiments,.
Randomization	All HCC and paired paracancerous tissues were obtained from Fujian Medical University Union Hospital and randomly used in this experiment (n = 28). All patients were diagnosed with HCC based on tissue specimens. The patients had not undergone chemotherapy, radiotherapy, or other new adjuvant therapy prior to surgery.
Blinding	Animals included in this study were randomly divided into control group and treatment group, and patients were divided into high and low expression groups according to the expression of target gene RBPJ.

Behavioural & social sciences study design

All studies must disclose on these points even when the disclosure is negative.

Study description	N/A
Research sample	N/A
Sampling strategy	N/A

Data collection	N/A
Timing	N/A
Data exclusions	N/A
Non-participation	N/A
Randomization	N/A

Ecological, evolutionary & environmental sciences study design

All studies must disclose on these points even when the disclosure is negative.

Study description	N/A
Research sample	N/A
Sampling strategy	N/A
Data collection	N/A
Timing and spatial scale	N/A
Data exclusions	N/A
Reproducibility	N/A
Randomization	N/A
Blinding	N/A

Did the study involve field work? ☐ Yes ☑ No

Field work, collection and transport

Field conditions	N/A
Location	N/A
Access & import/export	N/A
Disturbance	N/A

Reporting for specific materials, systems and methods

We require information from authors about some types of materials, experimental systems and methods used in many studies. Here, indicate whether each material, system or method listed is relevant to your study. If you are not sure if a list item applies to your research, read the appropriate section before selecting a response.

Materials & experimental systems

n/a	Involved in the study
☐	☑ Antibodies
☐	☑ Eukaryotic cell lines
☑	☐ Palaeontology and archaeology
☐	☑ Animals and other organisms
☑	☐ Clinical data
☑	☐ Dual use research of concern

Methods

n/a	Involved in the study
☐	☑ ChIP-seq
☐	☑ Flow cytometry
☑	☐ MRI-based neuroimaging

Antibodies

Antibodies used

We listed the specific information of the antibodies in the supplementary material section.

Validation

We confirmed the species and application of primary antibodies by Western blot and flow cytometry.

Eukaryotic cell lines

Policy information about cell lines and Sex and Gender in Research

Cell line source(s)

Hepatocellular carcinoma cell lines were purchased from Shanghai Institute of Biochemistry and Cell Biology. Primary T cells were isolated from C57BL/6J mice or healthy volunteers in our laboratory.

Authentication

Hepa1-6 cells（Cat# SCSP-512）；HepG2 cells（SCSP-510）；Huh-7 cells（SCSP-526）

Mycoplasma contamination

All cells in this study were randomly tested and found to be free of mycoplasma contamination.

Commonly misidentified lines
(See ICLAC register)

To study the killing effect of T cells on HCC, we selected three HCC cell lines, including HepG2, Huh-7 and Hepa1-6.

Palaeontology and Archaeology

Specimen provenance

N/A

Specimen deposition

N/A

Dating methods

N/A

☐ Tick this box to confirm that the raw and calibrated dates are available in the paper or in Supplementary Information.

Ethics oversight

N/A

Note that full information on the approval of the study protocol must also be provided in the manuscript.

Animals and other research organisms

Policy information about studies involving animals; ARRIVE guidelines recommended for reporting animal research, and Sex and Gender in Research

Laboratory animals

C57BL/6J and NOD/SCID were purchased from Vital River Laboratory Animal Technology Co. Ltd.

Wild animals	NA
Reporting on sex	Female C57BL/6J and NOD/SCID were used in this study.
Field-collected samples	Female mice (6 weeks old, 18~20 g) were housed in a SPF environment with a 12/12 hours day/night cycle.
Ethics oversight	This study protocol was reviewed and approved by the Experimental Animal Ethics Committee of Fujian Medical University, approval number IACUC FJMU 2022-0015.

Note that full information on the approval of the study protocol must also be provided in the manuscript.

Clinical data

Policy information about clinical studies

All manuscripts should comply with the ICMJE guidelines for publication of clinical research and a completed CONSORT checklist must be included with all submissions.

Clinical trial registration	N/A
Study protocol	N/A
Data collection	N/A
Outcomes	N/A

Dual use research of concern

Policy information about dual use research of concern

Hazards

Could the accidental, deliberate or reckless misuse of agents or technologies generated in the work, or the application of information presented in the manuscript, pose a threat to:

No	Yes	
☑	☐	Public health
☑	☐	National security
☑	☐	Crops and/or livestock
☑	☐	Ecosystems
☑	☐	Any other significant area

Experiments of concern

Does the work involve any of these experiments of concern:

No	Yes	
☑	☐	Demonstrate how to render a vaccine ineffective
☑	☐	Confer resistance to therapeutically useful antibiotics or antiviral agents
☑	☐	Enhance the virulence of a pathogen or render a nonpathogen virulent
☑	☐	Increase transmissibility of a pathogen
☑	☐	Alter the host range of a pathogen
☑	☐	Enable evasion of diagnostic/detection modalities
☑	☐	Enable the weaponization of a biological agent or toxin
☑	☐	Any other potentially harmful combination of experiments and agents

ChIP-seq

Data deposition

- [x] Confirm that both raw and final processed data have been deposited in a public database such as GEO.
- [x] Confirm that you have deposited or provided access to graph files (e.g. BED files) for the called peaks.

Data access links<br>*May remain private before publication.*	CUT&Tag sequencing of infiltrating CD8+ T cells from mouse normal liver and primary HCC (PRJNA904833, https://www.ncbi.nlm.nih.gov/bioproject/?term=PRJNA904833)are available at the SRA repository.
Files in database submission	Files included Normal_CD8_CUT&Tag_1, Normal_CD8_CUT&Tag_2, Normal_CD8_CUT&Tag_3, HCC_CD8_CUT&Tag_1, HCC_CD8_CUT&Tag_2, HCC_CD8_CUT&Tag_3.
Genome browser session (e.g. UCSC)	UCSC

Methodology

Replicates	The number of replicates is 3.
Sequencing depth	6G
Antibodies	RBPSUH (D10A4) XP® Rabbit mAb #5313 Cell signaling
Peak calling parameters	Some parameters to consider: * Adjust the sequence tags to better represent the original DNA fragment (by 'shifting tags in the 3 prime direction' or by 'extending tags' to the estimated length of the original fragment length) * Background model used * Use of strand dependent bimodality * fragment length, read length, replicates, duplication, total read count.
Data quality	Quality control is qualified.
Software	We Called ChIP-seq peaks using MACS2.

Flow Cytometry

Plots

Confirm that:

- [x] The axis labels state the marker and fluorochrome used (e.g. CD4-FITC).
- [x] The axis scales are clearly visible. Include numbers along axes only for bottom left plot of group (a 'group' is an analysis of identical markers).
- [x] All plots are contour plots with outliers or pseudocolor plots.
- [x] A numerical value for number of cells or percentage (with statistics) is provided.

Methodology

Sample preparation	Tumors were removed and minced and enzymatically digested with Tumor Dissociation Kit. The digested tissues were sieved through a 70um filter. Tumor-infiltrating lymphocytes were purified with CD45 (TIL) MicroBeads.
Instrument	Relative light units were detected using FACSCelesta™ Flow Cytometer (BD biosciences).
Software	Flowjo was used to analyze the results of fluorescence flow cytometry, and R software and CytoBank were used to analyze the results of mass spectrometry flow cytometry.
Cell population abundance	For both human and mouse specimens, CD8+T cells constitute 5% to 15% of leukocytes.
Gating strategy	FSC-A/SSC-A gate determined cell populations, FSC-A/FSC-H gate removed adherent cells, BV605-FVS575V-A/SSC-A gate determined viable cells, FITC-A/SSC-A gate determined leukocytes, APC-A/SSC-A gate determined T cells, and BV510-A/SSC-A gate identified CD8+T cells.PD-1 and TIM-3 were labeled with PE-CF594 and BV421, and IFN-γ and TNF-α were labeled with PE and BV421.

- [x] Tick this box to confirm that a figure exemplifying the gating strategy is provided in the Supplementary Information.

Magnetic resonance imaging

Experimental design

Design type	N/A

Design specifications | N/A

Behavioral performance measures | N/A

Acquisition

Imaging type(s) | N/A

Field strength | N/A

Sequence & imaging parameters | N/A

Area of acquisition | N/A

Diffusion MRI ☐ Used ☑ Not used

Preprocessing

Preprocessing software | N/A

Normalization | N/A

Normalization template | N/A

Noise and artifact removal | N/A

Volume censoring | N/A

Statistical modeling & inference

Model type and settings | N/A

Effect(s) tested | N/A

Specify type of analysis: ☐ Whole brain ☐ ROI-based ☐ Both

Statistic type for inference
(See Eklund et al. 2016) | N/A

Correction | N/A

Models & analysis

n/a	Involved in the study
☑	☐ Functional and/or effective connectivity
☑	☐ Graph analysis
☑	☐ Multivariate modeling or predictive analysis

Functional and/or effective connectivity | N/A

Graph analysis | N/A

Multivariate modeling and predictive analysis | N/A
